# The Association of Polymorphisms in Circadian Clock and Lipid Metabolism Genes With 2^nd^ Trimester Lipid Levels and Preterm Birth

**DOI:** 10.3389/fgene.2019.00540

**Published:** 2019-06-13

**Authors:** Ursa Kovac, Elizabeth A. Jasper, Caitlin J. Smith, Rebecca J. Baer, Bruce Bedell, Brittney M. Donovan, Nancy Weathers, Ursula Prosenc Zmrzljak, Laura L. Jelliffe-Pawlowski, Damjana Rozman, Kelli K. Ryckman

**Affiliations:** ^1^Centre for Functional Genomics and Bio-Chips, Institute of Biochemistry, Faculty of Medicine, University of Ljubljana, Ljubljana, Slovenia; ^2^Department of Epidemiology, The University of Iowa, Iowa City, IA, United States; ^3^Department of Pediatrics, University of California, San Diego, San Diego, CA, United States; ^4^California Preterm Birth Initiative, University of California, San Francisco, San Francisco, CA, United States; ^5^Department of Epidemiology and Biostatistics, University of California, San Francisco, San Francisco, CA, United States

**Keywords:** circadian clock, single nucleotide polymorphism, lipid metabolism, preterm birth, *PER3*, triglycerides

## Abstract

Deregulation of the circadian system in humans and animals can lead to various adverse reproductive outcomes due to genetic mutations and environmental factors. In addition to the clock, lipid metabolism may also play an important role in influencing reproductive outcomes. Despite the importance of the circadian clock and lipid metabolism in regulating birth timing few studies have examined the relationship between circadian genetics with lipid levels during pregnancy and their relationship with preterm birth (PTB). In this study we aimed to determine if single nucleotide polymorphisms (SNPs) in genes from the circadian clock and lipid metabolism influence 2^nd^ trimester maternal lipid levels and if this is associated with an increased risk for PTB. We genotyped 72 SNPs across 40 genes previously associated with various metabolic abnormalities on 930 women with 2^nd^ trimester serum lipid measurements. SNPs were analyzed for their relationship to levels of total cholesterol, high density lipoprotein (HDL), low density lipoprotein (LDL) and triglycerides (TG) using linear regression. SNPs were also evaluated for their relationship to PTB using logistic regression. Five SNPs in four genes met statistical significance after Bonferroni correction (*p* < 1.8 × 10^-4^) with one or more lipid levels. Of these, four SNPs were in lipid related metabolism genes: rs7412 in *APOE* with total cholesterol, HDL and LDL, rs646776 and rs599839 in C*ELSR2-PSRC1-SORT1* gene cluster with total cholesterol, HDL and LDL and rs738409 in *PNPLA3* with HDL and TG and one was in a circadian clock gene: rs228669 in *PER3* with TG. Of these SNPs only *PER3* rs228669 was marginally associated with PTB (*p* = 0.02). In addition, *PER3* rs228669 acts as an effect modifier on the relationship between TG and PTB.

## Introduction

Preterm birth (PTB) is a common and multifactorial condition in which lifestyle and clinical factors including hypertension, diabetes, and nutrition interact with environmental, genetic and epigenetic factors (Bick, 2012). The World Health Organization defines PTB as birth before 37 completed weeks of gestation. Annually, it affects almost 15 million pregnancies worldwide, and is the leading cause of death in children younger than 5 years of age (Blencowe et al., 2013).

The level of maternal blood lipids is important for maintaining a healthy pregnancy with normal fetal development. In pregnancy, multiple physiological changes occur that contribute to the alterations in lipid profiles of healthy, gestating women. The changes in lipid physiology throughout the course of pregnancy allow for proper nutrients for the fetus and reflect increasing insulin resistance in the mother. Several studies show that excessive changes in lipid levels are associated with increased risk for PTB (Mudd et al., 2012; Bream et al., 2013). Recently a meta-analysis demonstrated that elevated total cholesterol, triglycerides (TG) and low concentration of high-density lipoprotein (HDL) were associated with an increased risk of PTB (Jiang et al., 2017). It is still unclear whether the lipid levels directly affect PTB or if PTB is influenced indirectly through changes in lipid levels that are a result of the pregnancy. Causality of lipid exposures can begin to be addressed by examining PTB risk in relation to genetic predisposition toward certain lipid profiles.

Genome-wide association studies (GWAS) have individually investigated the genetic contribution to adult lipid levels and PTB (Teslovich et al., 2010; Willer et al., 2013; Zhang et al., 2017). There are numerous GWAS and candidate gene studies that have identified single nucleotide polymorphisms (SNPs) associated with various components of lipid metabolism (Chasman et al., 2008, 2009; Aulchenko et al., 2009; Sabatti et al., 2009; Angelakopoulou et al., 2012; Manning et al., 2012; Klarin et al., 2018). The circadian clock plays an important role in different parts of lipid metabolism. Disruption of the core molecular clock can lead to abnormal energy balance and dysregulation of lipids (Turek et al., 2005; Scott et al., 2008; Marcheva et al., 2010; Shimba et al., 2011; Kovanen et al., 2015). Several studies have identified SNPs from core circadian regulating genes including *CLOCK*, *ARNTL*, and *CRY2*, that are associated with lipid levels and metabolic syndrome (Scott et al., 2007; Englund et al., 2009; Garaulet et al., 2009; Sookoian et al., 2010; Tsuzaki et al., 2010; Garcia-Rios et al., 2012; Kovanen et al., 2015; Lin et al., 2017). Additionally, various aspects of reproductive physiology, such as the estrous cycle and parturition are regulated by the clock (Urlep and Rozman, 2013). Plasma cholesterol is known to vary depending on the time of day blood is sampled, further illustrating a potential connection between the circadian clock and cholesterol metabolism (Jones and Schoeller, 1990). Several studies in mice have shown that mutations in circadian clock genes abolish expression of genes that are critical for the regulation of cholesterol synthesis (Horvat et al., 2011).

A recent GWAS of PTB identified growth differentiation, immunity and endocrine function as one of the most common pathways associated with gestational age (Zhang et al., 2017). While the GWAS did not identify direct associations between circadian rhythm or lipid metabolism genes with PTB, all of the pathways identified, especially the endocrine system, are highly interconnected with lipid metabolism and thus have an important role in pathogenesis of several primary and secondary lipid metabolism disorders including dyslipidemia, obesity and type 2 diabetes. Given the clear biologic relationship between circadian rhythm, lipid metabolism and risk for PTB we sought to examine the relationship between candidate circadian and lipid metabolism genes with 2^nd^ trimester lipid levels and PTB in a population-based case-control study of 993 women.

## Materials and Methods

### Study Population

The study population was drawn from a population-based cohort of 757,853 singleton live births in the state of California born from July 2009 through December 2010. Study subjects included a nested case-control sampling of 992 (495 PTB cases and 497 controls) California women with a non-fasted 2^nd^ trimester (15–20 weeks gestation) serum sample banked by the California Biobank Program after it was used for routine prenatal screening. Serum samples were drawn between 15 and 20 weeks’ gestation and were not fasted. The population has been described previously (Jelliffe-Pawlowski et al., 2018). Demographic and obstetric factors evaluated included race/ethnicity, maternal age, body mass index, gestational age and gestational age at time of prenatal screening (15–20 weeks gestation). All variables were derived from a file linking birth certificate records to all hospital discharge records for the mother and baby from 1 year prior to the birth to 1 year after the birth. All subtypes of PTB (spontaneous labor and medically indicated) were based on criteria described previously (Jelliffe-Pawlowski et al., 2015).

### DNA Isolation and Lipid Measurements

Lipid levels including total cholesterol, HDL, low density lipoprotein (LDL), and TG were measured on Roche Cobas c 111 instrument on all serum samples. DNA was purified from the clotted material using a Quickgene-610L DNA extraction system (Autogen, Holliston, MA, United States). Methods and protocols for the study were approved by the Committee for the Protection of Human Subjects within the Health and Human Services Agency of the State of California.

### Genetic Marker Selection

**FIGURE 1:**
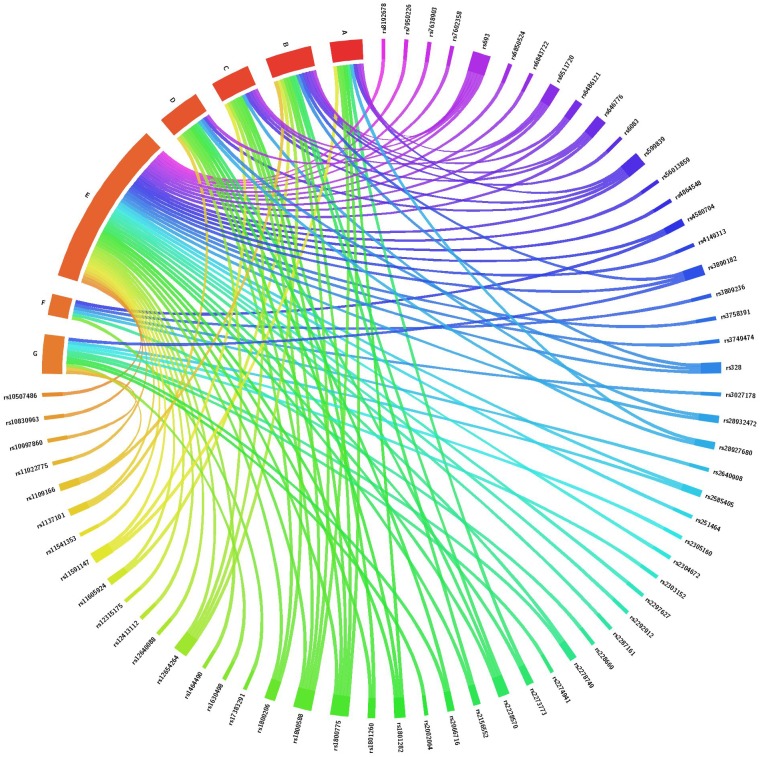
Association of SNPs from core circadian regulating genes, circadian-related and lipid-related genes with one or more lipid levels and/or with metabolic and reproductive phenotypes. This chord diagram is an illustration of Supplementary Tables S1, S2. Each colored line indicates a previously reported SNP-phenotype association. A, total cholesterol level; B, high density lipoprotein level; C, low density lipoprotein level; D, triglyceride level; E, metabolic phenotypes including obesity, body mass index, fasting blood glucose, hemoglobin A1C, gestational diabetes, hyperglycemia, hypertension, cardiovascular disease, ischemic stroke, dyslipidemia, fatty acid composition and C-reactive protein; F, conditions of the liver and colon including hepatocellular carcinoma, non-alcoholic fatty liver disease and colorectal cancer; G, reproductive phenotypes including gestational age, birth weight and prematurity.

A total of 72 candidate SNPs were selected based on their relevance to circadian rhythm or lipid metabolism and their availability in the laboratory (Supplementary Tables S1, S2). Of the total SNPs 25 were from core circadian regulating genes and 47 were from circadian-related or lipid-related genes. There were 10 SNPs related to lipid metabolism that were selected based on a previous study of PTB in our laboratory (Steffen et al., 2007). Of the 72 total SNPs, 32 were directly related to one or more lipid levels (Figure 1 and Supplementary Tables S1, S2). The remainder were associated with metabolic-related phenotypes including dyslipidemia, obesity, type 2 diabetes, body mass index, non-alcoholic fatty liver disease and hepatocellular carcinoma or reproductive complications such as PTB (Figure 1 and Supplementary Tables S1, S2).

### Genotyping and Quality Control

A total of 930 subjects with purified DNA were selected for genotyping. Genotyping procedures have been described previously (Ryckman et al., 2014). TaqMan assays (Applied Biosystems, Foster City, CA, United States) for the 72 markers were tested on control DNA prior to genotyping samples on the EP1 SNP Genotyping System and GT 192.24 Dynamic Array Integrated Fluidic Circuits (Fluidigm, San Francisco, CA, United States). Seventy SNP genotyping assays were available and ordered using assay-on-demand service from Applied Biosystems; two assays were custom designed also from Applied Biosystems. Three CEPH individuals (Coriell Institute, Camden, NJ, United States) served as positive controls and double-distilled water was used as negative controls. SNPs were eliminated based on low genotyping efficiency (< 90%) and deviation from Hardy-Weinberg equilibrium (*P* < 0.01). Four SNPs were excluded for genotyping efficiency < 90%: rs2274941, rs12654264, rs10997860, and rs11605924. A total of 68 markers from circadian clock metabolism and lipid metabolism were considered for association with 2^nd^ trimester lipid levels including total cholesterol, HDL LDL and TG. Twenty-six subjects were excluded for genotyping efficiency < 90%, resulting in a final sample size of 904 subjects (450 PTB cases and 454 term controls). Differences in minor allele frequency by race were tested with chi-square tests.

### Statistical Analysis

Statistical analyses were performed using Plink software (Broad Institute, Cambridge, MA, United States) and Statistical Analysis Software – SAS version 9.4 (SAS Institute, Cary, NC, United States). All four lipid measurements approximated a normal distribution. Each SNP-lipid level combination was tested for association using linear regression. Correction for multiple testing was achieved using the Bonferroni method [0.05/(68 SNPs × 4 lipid measurements) = *p* < 1.8 × 10^-4^]. SNP-lipid combinations that were significant after Bonferroni correction were then evaluated including relevant covariates: gestational age at time of sampling (GA), body mass index (BMI) and race. SNPs that were remained significantly associated with lipid(s) after adjustment for covariates were assessed for association with PTB using logistic regression. SNPs associated with both lipids and PTB were assessed as possible effect modifiers of the lipid-PTB relationship by including a SNP-lipid interaction term in the logistic regression models. For significant interactions separate logistic regression models were performed stratified by SNP genotype.

### Ethics Statement

Data from the California Prenatal and Newborn Screening Programs were obtained through the California Biobank Program (Screening Information System request no. 476). Data were obtained with an agreement that the California Department of Public Health is not responsible for the results or conclusions drawn by the authors of this publication. Methods and protocols were approved by the Committee for the Protection of Human Subjects within the Health and Human Services Agency of the State of California. All data was de-identified and determined not to qualify as human subjects research by The University of Iowa Institutional Review Board.

### Data Availability

The data used in this analysis is owned by the State of California who grants access through an application and approval process. This process is open to any interested researcher or other investigator who seeks access. No special permission was granted for this project. Interested researchers may apply for access to the data at: https://www.cdph.ca.gov/Programs/CFH/DGDS/Pages/cbp/default.aspx.

## Results

### Demographic Characteristics of the Study Population

The analysis included 904 mothers: 454 with term birth and 450 with PTB (Table 1). The mean gestational age was 38.9 in the term group and 32.4 in the preterm group (*p* < 0.001). Mothers with preterm and term birth differed by gestational age at birth but did not differ by gestational age at screening, BMI, race or maternal age. The majority of the women were Hispanic white (approximately 50% in both groups) and non-Hispanic white (approximately 35% in both groups).

**Table 1:** Demographic characteristics of the study population (*N* = 904).

	Term N (%)	Preterm N (%)	*p*-value
**Sample size**	454 (50.2)	450 (49.8)	
**Gestational age (weeks)^†^**	38.9 ± 1.0	32.4 ± 3.8	< 0.001
**Gestational age at screening (weeks)^†^**	16.6 ± 1.1	16.4 ± 1.0	0.085
**Maternal BMI**	25.2 ± 5.6	26.0 ± 6.6	0.261
**Maternal race**			0.795
*Asian*	43 (9.5)	51 (11.3)	
*Hispanic*	234 (51.5)	232 (51.6)	
*Non-Hispanic White*	163 (35.9)	155 (34.4)	
*Unknown*	14 (3.1)	12 (2.7)	
**Maternal age group**			0.354
*< 18 years*	4 (0.9)	7 (1.6)	
*18–34 years*	325 (71.6)	305 (67.8)	
*> 34 years*	125 (27.5)	138 (30.7)	
**Preterm birth subgroups**			N/A
*Spontaneous*	N/A	76 (16.9)	
*Provider initiated*	N/A	357 (79.3)	
*Subtype unknown*	N/A	17 (3.8)	


*^†^Data are expressed as mean ± SD. Variables may contain missing data. N/A indicates the data was unavailable.*

### Genetic Association With 2^nd^ Trimester Lipid Levels

**FIGURE 2:**
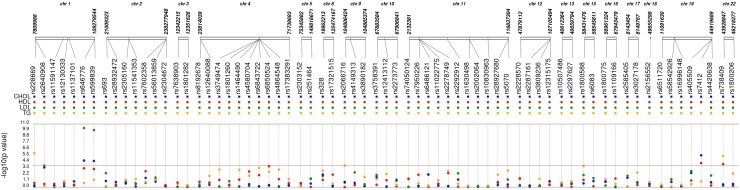
Synthesis-view data visualization of 68 single nucleotide polymorphisms (SNPs) with maternal lipid levels. Each dot represents the unadjusted association with a candidate single nucleotide polymorphism and CHOL, total cholesterol (blue); HDL, high density lipoprotein (red); LDL, low density lipoprotein (green); and TG, triglycerides (yellow).

Of the 72 candidate SNPs across 40 genes, 68 met the quality criteria for further analysis. Of these 45 SNPs were associated with one or more lipid levels at *p*-value < 0.05 (Figure 2 and Supplementary Table S3). After the correction for multiple testing (Bonferroni-corrected *p*-value < 1.8 × 10^-4^), five SNPs in four genes were associated with one or more 2^nd^ trimester lipid levels (Table 2). Six additional SNPs from circadian clock gene candidates (5 SNPs in *CLOCK* and 1 in *PER3*) and 2 lipid related SNPs (1 SNP in *LIPC* and 1 SNP in *ABCA1*) nearly met Bonferroni correction (*p* < 1 × 10^-3^) with one or more lipid levels (Supplementary Table S3).

**Table 2:** Second trimester lipid levels by SNP for significant associations.

	Mean (SD) [mg/dL]			
SNP	CHOL	HDL	LDL	TG
***APOE*** **rs7412**				
CC (*N* = 814)	211.76 (36.13)	73.96 (16.70)	124.90 (33.24)	204.27 (73.75)
CT (*N* = 84)	192.31 (34.65)	81.76 (19.19)	97.73 (27.91)	190.58 (68.43)
***p*-value**	2.81 × 10^-6^	6.35 × 10^-5^	< 1.0 × 10^-12^	0.10
***CELSR2*** **rs646776**				
AA (*N* = 605)	213.64 (36.99)	73.50 (16.43)	127.34 (34.18)	202.71 (72.15)
GA (*N* = 263)	203.24 (33.83)	76.40 (17.60)	113.35 (30.55)	206.50 (77.40)
GG (*N* = 36)	195.08 (35.79)	83.47 (21.63)	100.89 (29.49)	183.00 (56.26)
***p*-value**	2.35 × 10^-5^	5.35 × 10^-4^	5.1 × 10^-11^	0.19
***CELSR2*** **rs599839**				
GG (*N* = 577)	213.81 (37.27)	73.60 (16.44)	127.56 (34.42)	202.37 (72.10)
GT (*N* = 275)	203.69 (33.83)	75.60 (17.50)	114.23 (30.50)	208.23 (77.90)
TT (*N* = 50)	197.16 (33.14)	82.84 (20.64)	103.68 (29.69)	181.262 (55.88)
***p*-value**	2.83 × 10^-5^	7.10 × 10^-5^	1.16 × 10^-10^	0.054
***PNPLA3*** **rs738409**				
CC (*N* = 385)	210.07 (36.42)	76.43 (17.12)	122.28 (33.34)	191.78 (70.42)
CG (*N* = 373)	211.22 (37.20)	75.10 (16.76)	123.20 (34.40)	205.34 (64.37)
GG (*N* = 146)	205.95 (34.45)	69.36 (17.10)	119.55 (33.74)	226.77 (93.37)
***p*-value**	0.33	9.81 × 10^-5^	0.54	3.56 × 10^-5^
***PER3*** **rs228669**				
AA (*N* = 50)	208.04 (30.82)	70.44 (16.46)	120.22 (30.80)	237.66 (84.21)
GA (*N* = 273)	212.01 (38.34)	74.93 (17.22)	123.18 (35.05)	215.23 (84.81)
GG (*N* = 576)	209.02 (35.00)	75.03 (17.07)	121.96 (33.54)	194.34 (64.19)
***p*-value**	0.50	0.19	0.81	1.24 × 10^-6^


*SD, standard deviation; CHOL, total cholesterol; LDL, low-density lipoprotein; HDL, high-density lipoprotein; TG, triglycerides.*

Rs7412 in the *APOE* gene was significantly associated with total cholesterol, LDL and HDL but not TG (Table 2). The CC genotype was associated with higher levels of total cholesterol and LDL and lower levels of HDL compared to those with the CT genotype. No individuals were homozygous for the T allele. Two SNPs, rs646776 and rs599839 from C*ELSR2-PSRC1-SORT1* cholesterol gene cluster were significantly associated with LDL and total cholesterol and marginally associated with HDL but not TG. Each addition of the A allele for rs646776 was associated with an increase in total cholesterol and LDL levels and a decrease in HDL levels. For rs599839 each addition of the G allele was associated with an increase in total cholesterol and LDL levels and a decrease in HDL levels (Table 2). A coding region polymorphism, rs738409, encoding I148M in patatin-like phospholipase domain containing 3 (*PNPLA3*), synonym adiponutrin (Kovac and Rozman, 2015), was significantly associated with TG and HDL (Table 2). Each additional G allele of rs738409 was associated with higher TG and lower HDL. For the circadian clock metabolism markers, rs228669 in the *PER3* gene was significantly associated with TG but not total cholesterol, HDL or LDL. Each addition of the A allele for rs228669 in the *PER3* gene was significantly associated with higher levels of TG. All statistically significant associations remained after adjusting for gestational age at sampling, BMI and race (Table 3). We also performed a sensitivity analysis in White individuals (Hispanic and non-Hispanic) and the results remained significant when excluding Asian individuals.

**Table 3:** Effect sizes for significant (Bonferroni-corrected *p*-value < 1.8 × 10^-4^) genetic associations with 2^nd^ trimester lipid levels adjusted for relevant covariates (BMI, GA, race).

	β (SE; *p*-value)			
	CHOL	HDL	LDL	TG
***APOE*** **rs7412**		
CC (*N* = 814)				N/S
Unadjusted	19.45 (4.13; 2.81 × 10^–6^)	–7.80 (1.94; 6.35 × 10^–5^)	27.18 (3.76; < 1.0 × 10^–12^)	
Adjusted for BMI, GA, race	19.40 (4.29; 6.8 × 10^–6^)	–7.47 (2.0 1; 2.1 × 10^–4^)	27.02 (3.9; 1.0 × 10^–11^)	
CT (*N* = 84) Referent	Referent	Referent	Referent	N/S
***CELSR2*** **rs646776**		
AA (*N* = 605)				N/S
Unadjusted	18.56 (6.18; 2.77 × 10^–3^)	–9.97 (2.92; 6.59 × 10^–4^)	26.45 (5.66; 3.40 × 10^–6^)	
Adjusted for BMI, GA, race	17.57 (6.38; 6.00 × 10^–3^)	–10.78 (2.98; 3.2 × 10^–4^)	26.60 (5.84; 6.00 × 10^–6^)	
GA (*N* = 263)				N/S
Unadjusted	8.16 (6.41; 0.20)	–7.08 (3.02; 0.02)	12.46 (5.86; 3.39 × 10^–2^)	
Adjusted for BMI, GA, race	7.60 (6.60; 0.25)	–7.83 (3.09; 1.1 × 10^–2^)	12.86 (6.05; 3.38 × 10^–2^)	
GG (*N* = 36)	Referent	Referent	Referent	N/S
***CELSR2*** **rs599839**		
GG (*N* = 577)				N/S
Unadjusted	16.65 (5.31; 1.79 × 10^–3^)	–9.24 (2.51; 2.43 × 10^–4^)	23.88 (4.87; 1.11 × 10^–6^)	
Adjusted for BMI, GA, race	16.99 (5.48; 2.00 × 10^–3^)	–9.16 (2.57; 3.80 × 10^–4^)	24.83 (5.03; 9.36 × 10^–7^)	
GT (*N* = 275)				N/S
Unadjusted	6.53 (5.54; 0,24)	–7.24 (2.62; 5.75 × 10^–3^)	10.55 (5.078; 3.80 × 10^–2^)	
Adjusted for BMI, GA, race	7.65 (5.72; 0.18)	–6.77 (2.68; 1.18 × 10^–2^)	12.11 (5.24; 2.11 × 10^–2^)	
TT (*N* = 50)	Referent	Referent	Referent	N/S
***PNPLA3*** **rs738409**		
CC (*N* = 385)	N/S		N/S	
Unadjusted		7.06 (1.65; 2.02 × 10^–5^)		–34.99 (7.03; 7.65 × 10^–7^)
Adjusted for BMI, GA, race		6.64 (1.68; 8.63 × 10^–5^)		–32.89 (7.23; 6.18 × 10^–6^)
CG (*N* = 373)	N/S		N/S	
Unadjusted		5.74 (1.66; 5.57 × 10^–4^)		–21.43 (7.058; 2.46 × 10^–3^)
Adjusted for BMI, GA, race		5.23 (1.69; 2.02 × 10^–3^)		–17.71 (7.25; 1.48 × 10^–2^)
GG (*N* = 146)	N/S	Referent	N/S	Referent
***PER3*** **rs228669**		
AA (*N* = 50)	N/S	N/S	N/S	
Unadjusted				43.32 (10.65; 5.16 × 10^–5^)
Adjusted for BMI, GA, race				43.21 (10.84; 7.24 × 10^–5^)
GA (*N* = 273)	N/S	N/S	N/S	
Unadjusted				20.89 (5.31; 8.91 × 10^–5^)
Adjusted for BMI, GA, race				20.90 (5.44; 1.30 × 10^–4^)
GG (*N* = 576)	N/S	N/S	N/S	Referent


*N/S indicates the association was not significant at the Bonferroni-corrected threshold of p-value < 1.8 × 10^–*4*^. β, beta coefficient; SD, standard deviation; BMI, body mass index; GA, gestational age at time of sampling; CHOL, cholesterol; LDL, low-density lipoprotein; HDL, high-density lipoprotein; TG, triglycerides.*

### Genetic Association With PTB

None of the 4 SNPs from the lipid metabolism genes that were significantly associated with 2^nd^ trimester were associated with any PTB or spontaneous PTB only (Table 4). The AA genotype of rs228669 within the *PER3* gene was marginally associated with an increased risk for PTB (Table 4). This association remained when limiting the sample to only spontaneous PTB (*p* = 0.01). The minor allele frequency for rs228669 was significantly different in Asians compared to Hispanics or Non-Hispanic Whites (Supplementary Table S4). When examining only Hispanic and Non-Hispanic White individuals the association with PTB remained (*p* = 0.04). TG were not associated with PTB in our sample population (OR = 1.00; 95% CI: 0.997–1.002). Therefore, we investigated whether rs228669 is an effect modifier of the relationship between TG and PTB. The interaction between rs228669 and TG levels was significant for the GA vs. AA (*p* = 0.01) genotype and marginally significant the GG vs. AA (*p* = 0.06). When stratifying by genotype lower TG levels were associated with PTB in individuals with the AA genotype (*p* = 0.05) whereas higher TG levels were associated with PTB in individuals with the GA genotype (*p* = 0.04) (Table 5). There is no association between TG levels and PTB in individuals with the GG genotype (*p* = 0.97).

**Table 4:** Genetic associations with PTB.

SNP	Term N (%)	PTB N (%)	*p*-value Full Cohort	*p*-value Spontaneous PTB
***APOE*** **rs7412**			0.97	0.89
CC (*N* = 814)	409 (90.7%)	405 (90.6%)		
CT (*N* = 84)	42 (9.3%)	42 (9.4%)		
TT (*N* = 0)	0	0		
***CELSR2* rs646776**			0.55	0.30
AA (*N* = 605)	311 (68.5%)	294 (65.3%)		
GA (*N* = 263)	127 (28.0%)	136 (30.22%)		
GG (*N* = 36)	16 (3.5%)	20 (4.4%)		
***CELSR2* rs599839**			0.29	0.15
GG (*N* = 577)	300 (66.4%)	277 (61.6%)		
GT (*N* = 275)	130 (28.8%)	145 (32.2%)		
TT (*N* = 50)	22 (4.9%)	28 (6.2%)		
***PNPLA3* rs738409**			0.05	0.06
CC (*N* = 385)	177 (39.0%)	208 (46.2%)		
CG (*N* = 373)	205 (45.2%)	168 (37.3%)		
GG (*N* = 146)	72 (15.9%)	74 (16.4%)		
***PER3* rs228669**			0.02	0.01
AA (*N* = 50)	16 (3.5%)	34 (7.6%)		
GA (*N* = 273)	146 (32.2%)	127 (28.4%)		
GG (*N* = 576)	290 (64.2%)	286 (64%)		


*p-values represent the univariate analysis between each SNP and preterm birth (PTB).*

**Table 5:** The association between TG levels and PTB s stratified by rs228669 *PER3* genotype.

*PER3* rs228669	Term	PTB	*p*-value
AA	275.9 (110.5)	219.7 (62.8)	0.05
	*N* = 16	*N* = 34	
GA	205.0 (73.6)	226.9 (95.1)	0.04
	*N* = 146	*N* = 127	
GG	194.4 (62.4)	194.2 (66.1)	0.97
	*N* = 290	*N* = 286	


*Values are reported as mean (SD) with the number of observations (N) for each cell reported. *p*-values represent the association between TG levels and PTB for each *PER3* rs228669 genotype.*

## Discussion

Preterm birth (PTB) is a major medical and public health concern. Several independent studies have evaluated the associations between maternal dyslipidemia and PTB (Catov et al., 2007; Edison et al., 2007; Toleikyte et al., 2011; Mudd et al., 2012; Vrijkotte et al., 2012; Emet et al., 2013; Jin et al., 2016). A recent meta-analysis demonstrated that elevated total cholesterol and TG were associated with an increased risk of PTB, meaning that abnormal levels of maternal lipids during pregnancy may have an impact on adverse pregnancy outcomes (Jiang et al., 2017). The circadian clock plays an important role in orchestrating lipid metabolism (Gooley and Chua, 2014). This study examines the association between polymorphisms in circadian clock and lipid metabolism genes with 2^nd^ trimester lipid levels and PTB. We identified a variant from the core circadian regulating genes that was significantly associated with 2^nd^ trimester lipid levels. Several variants within circadian- or lipid-related candidate genes that were previously reported to be associated with lipid profiles in non-pregnant adults were significantly associated with 2^nd^ trimester lipid levels in pregnant women.

One of the strongest associations identified was rs7412, a missense variant, in the *APOE* gene with total cholesterol, LDL and HDL. This variant has previously been shown to associate with various abnormalities in lipid metabolism and with PTB; however, the literature is not consistent on the strength or significance of this effect (Steffen et al., 2007; Li et al., 2015; Jacobs et al., 2016). The rs7412 (T) allele, also known as Arg176Cys, generally indicates the presence of an Apo-𝜀2 allele (Ghebranious et al., 2005). In our study, no individuals were homozygous for the T allele; however, individuals heterozygous for rs7412 had significantly lower total cholesterol and LDL and higher HDL than those homozygous for the A allele indicating a potential protective effect from a dyslipidemia profile in those heterozygotes for the Apo-𝜀2 allele. Our study, however, showed no relationship between the rs7412 genotype and PTB.

Two strong associations were identified between two regulatory region variants, rs646776 and rs599839, in the *CELSR2-PSRC1-SORT1* gene cluster with LDL and total cholesterol. Both variations where previously reported to be associated with LDL and total cholesterol (Muendlein et al., 2009; Nakayama et al., 2009; Lu et al., 2010; Angelakopoulou et al., 2012; Walia et al., 2014). These SNPs are in strong linkage disequilibrium (*r*^2^ > 0.86) for European and Hispanic populations based on data from 1000 genomes^1^. This lipid related gene cluster includes three distinct genes, located on the chromosome 1p13.3 region: The first gene from the cluster, *CELSR2*, encodes a cadherin involved in cell adhesion, the second gene, *PSRC1*, encodes a protein which plays a role in microtubule destabilization while the last gene, *SORT1*, encodes a protein involved in the lipid transport (Arvind et al., 2014). Our findings are in the same direction as the other published reports of associations with adult lipid levels (Willer et al., 2013). Neither SNP was associated with PTB.

The *PNPLA3* missense variant, rs738409, is the most consistently replicated genetic risk factor for non-alcoholic fatty liver disease (Romeo et al., 2008; Kovac and Rozman, 2015). In our study the G allele of rs738409 was associated with significantly higher TG, and significantly lower HDL compared to those homozygous for C allele or heterozygous. *PNPLA3* is a triacylglycerol lipase that mediates triacylglycerol hydrolysis in adipocytes. The protein may also be involved in the balance of energy in adipocytes (Kovac and Rozman, 2015). Romeo et al. (2008) reported that those homozygous for the G allele had more than twofold higher hepatic fat than non-carriers. Our findings are consistent with previous studies by demonstrating a significant relationship between the G allele rs738409 and higher 2^nd^ trimester TG levels and lower HDL levels. This SNP was not significantly associated with PTB.

The *PER3* synonymous polymorphism, rs228669, was significantly associated with 2^nd^ trimester TG levels and marginally associated with PTB. The significant relationship of this SNP to 2^nd^ trimester TG levels supports the findings that lipid and circadian clock metabolism are interconnected. *PER3* is a member of the Period family of genes and is expressed in a circadian pattern in the suprachiasmatic nucleus, the primary circadian pacemaker in the mammalian brain. Polymorphisms in this gene have been mostly linked to sleep disorders (Hong et al., 2015; Dallaspezia et al., 2016). Sleep disorders were recently reported to be strongly associated with PTB, further supporting the results of our study (Felder et al., 2017). Additionally, we identified a modest interaction between rs228669 and TG on the association with PTB. We observed an antagonistic interaction where increased TG was associated with PTB in individuals with the GA genotype and decreased TG was associated with PTB in individuals with the AA genotype. This finding suggests that rs228669 acts as an effect modifier of the relationship between TG and PTB and may explain some of the inconsistent findings reported on this relationship (Jiang et al., 2017). Additional studies are needed to further elucidate the role of this polymorphism with TG levels during pregnancy and the relationship to PTB.

This study has several limitations that should be considered when interpreting the findings. First, the lack of pre-pregnancy lipid levels, which would allow us to examine the role of the change in lipid levels during pregnancy in PTB. Longitudinal studies are necessary to fully examine the role of lipid metabolism in the pathophysiology of PTB. Second, non-fasting samples were used which could have a potential effect on the power of study. However, there is little substantial evidence that fasting lipids levels are superior to non-fasting samples when addressing the prognostic value of lipoproteins and lipids (Driver et al., 2016). In fact, there is robust evidence to show that non-fasting blood sampling could become a routine practice worldwide (Farukhi and Mora, 2016). Third, our sample was relatively small and included multiple racial and ethnic groups. We adjusted for race and assessed minor allele differences between racial and ethnic groups for all SNPs; however, future well-powered studies are needed in order to perform ancestry-specific analyses which could shed more light on the racial and ethnic disparities that exist in the prevalence of PTB. To our knowledge this is the first study that focuses on the associations between polymorphisms in circadian clock and lipid metabolism genes with 2^nd^ trimester lipid levels and PTB. We identified a modest interaction between a SNP in *PER3* and TG on the association with PTB. These findings may shed light on the role of TG in PTB and explain the inconsistency in some reported associations of TG with PTB. More studies are needed to fully examine the influence of circadian clock genetics on PTB and the relationship with lipid levels during pregnancy.

## Ethics Statement

Data from the California Prenatal and Newborn Screening Programs were obtained through the California Biobank Program (Screening Information System request no. 476). Data were obtained with an agreement that the California Department of Public Health is not responsible for the results or conclusions drawn by the authors of this publication. Methods and protocols were approved by the Committee for the Protection of Human Subjects within the Health and Human Services Agency of the State of California. All data was de-identified and determined not to qualify as human subjects research by The University of Iowa Institutional Review Board.

## Author Contributions

UK, UPZ, CS, KR, DR, and LJ-P: study concept and design. UK, CS, EJ, RB, BB, BD, and NW: data analysis and acquisition. UK and KR: drafting of initial manuscript. UK, CS, EJ, RB, BB, BD, NW, UPZ, LJ-P, DR, and KR: edits and approval of final manuscript.

## Conflict of Interest Statement

The authors declare that the research was conducted in the absence of any commercial or financial relationships that could be construed as a potential conflict of interest.

## Footnotes

1 http://useast.ensembl.org/index.html

## Abbreviations

CHOL: cholesterol
HDL: high density lipoprotein
LBW: low birth weight
LDL: low density lipoprotein
PTB: preterm birth
SNP: single nucleotide polymorphism
TG: triglycerides
