# Supplementary material for: The Association of Polymorphisms in Circadian Clock and Lipid Metabolism Genes With 2nd Trimester Lipid Levels and Preterm Birth
Source: Front Genet. 2019 Jun 13;10:540. doi: 10.3389/fgene.2019.00540 (PMC6584752; doi:10.3389/fgene.2019.00540)
Supplement: Supplementary file 1 [file Presentation_1.pdf]

Supplementary Table S1: Previously reported metabolic and reproductive associations with selected candidate SNPs from core circadian regulating genes.

Gene/Gene name/ SNP	Functional consequence	Relevant Metabolic and Reproductive Associations	References
CLOCK **Clock Circadian Regulator**			
rs3749474	3’ UTR	weight loss, obesity, BMI, infertility	(Garaulet et al., 2009, 2010; Loria-Kohen et al., 2016; Shen et al., 2015b)
rs4580704	intron variant	metabolic syndrome, obesity, overweight, BMI, type-2 diabetes, coronary heart disease related dyslipidemia, non-alcoholic fatty liver disease	(Bandín et al., 2013; Corella et al., 2016; Garaulet et al., 2009, 2010, 2011; Gomez-Delgado et al., 2015: Sookoian et al., 2007, 2008)
rs1464490	intron variant	obesity	(Corella et al., 2016; Garaulet et al., 2010)
rs6843722	intron variant	obesity	(Sookoian et al., 2007, 2008; Valladares et al., 2015)
rs6850524	intron variant	metabolic syndrome, obesity, idiopathic recurrent spontaneous abortion, infertility	(Hodžić et al., 2013, 2018; Sookoian et al., 2008, 2010; Ye et al., 2016)
rs4864548	non-coding transcript exon variant	metabolic syndrome, obesity, type-2 diabetes	(Krishnan et al., 2017; Scott et al., 2008; Sookoian et al., 2008, 2010; Uemura et al., 2016)
rs1801260	3’ UTR	insulin metabolism, metabolic syndrome, fatty acids, obesity, LDL cholesterol	(Bandín et al., 2013; Galbete et al., 2012; Garaulet et al., 2009, 2010, 2011, 2012; Garcia-Rios et al., 2014; Scott et al., 2008; Shen et al., 2015b; Tsuzaki et al., 2010; Uemura et al., 2016)
ARNTL **Aryl Hydrocarbon Receptor Nuclear Translocator Like**			
rs2278749	intron variant	infertility	(Kovanen et al., 2010)
rs6486121	intron variant	type-2 diabetes, hypertension, HDL cholesterol	(Klarin et al., 2018; Woon et al., 2007)

rs7950226	intron variant	type-2 diabetes, gestational diabetes mellitus, hypertension, metabolic syndrome	(Kelly et al., 2012; Pappa et al., 2013; Woon et al., 2007)
rs11022775	intron variant	gestational diabetes mellitus, type-2 diabetes	(Kelly et al., 2012; Pappa et al., 2013; Woon et al., 2007)
PER1 **Period Circadian regulator 1**			
rs2585405	missense variant	reproductive complications	(Chu et al., 2008)
rs3027178	synonymous variant	hepatocellular carcinoma	(Zhang et al., 2014)
PER2 **Period Circadian regulator 2**			
rs2304672	5’ UTR	plasma fatty acid composition, obesity	(Garaulet et al., 2010; Garcia-Rios et al., 2012)
rs56013859	intron variant	fasting blood glucose	(Englund et al., 2009)
rs7602358	intron variant	type-2 diabetes	(Kelly et al., 2012)
PER3 **Period Circadian regulator 3**			
rs228669	synonymous variant	hepatocellular carcinoma	(Zhang et al., 2014)
rs2640908	synonymous variant	hepatocellular carcinoma	(Zhang et al., 2014; Zhao et al., 2012)
CRY1 **Cryptochrome Circadian regulator 3**			
rs2287161	regulatory region variant	insulin resistance	(Dashti et al., 2014)
rs3809236	5’ UTR	hepatocellular carcinoma	(Zhang et al., 2014)
rs12315175	intron variant	type-2 diabetes	(Kelly et al., 2012)
CRY2 **Cryptochrome Circadian regulator 2**			
rs2292912	non coding transcript exon variant	type-2 diabetes	(Kelly et al., 2012)

rs11605924	intron variant	type-2 diabetes, fasting glucose glucose metabolism, HDL cholesterol	(Barker et al., 2011; Dashti et al., 2015; Dupuis et al., 2010; Hu et al., 2010; Langlois et al., 2016; Manning et al., 2012; Mirzaei et al., 2014; Renström et al., 2015)
NPAS2 **Neuronal PAS domain Protein 2**			
rs2305160	missense variant	reproductive complications	(Yuan et al., 2014; Kovanen et al., 2010; Chu et al., 2008)
rs11541353	missense variant	hypertension	(Englund et al., 2009)

Supplementary Table S2: Previously reported metabolic and reproductive associations with selected candidate SNPs for circadian-related and lipid-related genes.

Gene/Gene name/ SNPs	Functional consequence	Relevant Metabolic and Reproductive Associations	References
SIRT1 **Sirtuin 1**			
rs12413112	intron variant	BMI	(Clark et al., 2012)
rs3758391	intergenic variant	type- 2 diabetes	(Cruz et al., 2010)
rs2273773	synonymous variant	BMI, hypertension, total cholesterol, hyperglycemia, obesity	(Berg et al., 2009; Kilic et al., 2014; Shimoyama et al., 2011, 2012; Zhong et al., 2015b)
rs10997860	intron variant	reproductive complications, placental abruption	(Workalemahu et al., 2013)
CELSR2-PSRC1-SORT1 **gene cluster Cadherin, EGF LAG Seven-pass G-type Receptor 2, Proline and Serine rich coiled-coil 1, Sortilin 1**			
rs646776	regulatory region variant	LDL cholesterol, HDL cholesterol, C-reactive protein, blood protein levels, coronary artery disease	(Arvind et al., 2014; Aulchenko et al., 2009; Chasman et al., 2008; Consortium, 2011; Devaney et al., 2011; Dumitrescu et al., 2011; Gigante et al., 2012; Grallert et al., 2012; Guo et al., 2015a; He et al., 2017; Hubacek et al., 2017; Jansen et al., 2014; Kanai et al., 2018; Kathiresan et al., 2008; Keebler et al., 2010; Keebler Mary E. et al., 2009; Lanktree et al., 2009; Ligthart et al., 2016; Lu et al., 2010; Muendlein et al., 2009; Murray et al., 2009; Qi et al., 2011a, 2011b; Reilly et al., 2011; Saleheen et al., 2010; Sandhu et al., 2008; Shirts et al., 2011; Suhre et al., 2017; Sun et al., 2018; Surakka et al., 2015; Walia et al., 2014; Wallace et al., 2008; Zhou et al., 2015)

rs599839	regulatory region variant	LDL cholesterol, fasting glucose, coronary artery disease, total cholesterol, triglycerides	(Abe et al., 2015; Angelakopoulou et al., 2012; Breitling et al., 2015; Cho et al., 2009; Coronary Artery Disease Consortium et al., 2009; Ellis et al., 2011; Fujimaki et al., 2015; Gigante et al., 2012; Grallert et al., 2012; Guo et al., 2015a; He et al., 2017; Kathiresan et al., 2008; Kleber et al., 2010; Linsel-Nitschke et al., 2010; Ma et al., 2010; Ogawa et al., 2010; Roslin et al., 2009; Samani et al., 2008; Sandhu et al., 2008; Schunkert et al., 2011; Spracklen et al., 2017; Wallace et al., 2008; Wang et al., 2011a; Willer et al., 2008; Zhou et al., 2011, 2015)
POMC **Proopiomelanocortin**			
rs28932472	missense variant	LDL cholesterol, blood pressure	(Queiroz et al., 2015)
PPARA **Peroxisome Proliferator Activated Receptor Alpha**			
rs1800206	missense variant	type-2 diabetes, obesity, LDL cholesterol, dyslipidemia, hypertriglyceridemia	(Alsaleh et al., 2011; Andrulionytė et al., 2007; Bouchard-Mercier et al., 2011; Costa-Urrutia et al., 2017; Dong et al., 2015; Fan et al., 2015; Gu et al., 2015)
PPARG **Peroxisome Proliferator Activated Receptor Gamma**			
rs7638903	intron variant	type-2 diabetes,	(Claussnitzer et al., 2014)
rs1801282	missense variant	type-2 diabetes, BMI, insulin resistance, total cholesterol, triglycerides, obesity, hypertension, metabolic syndrome, coronary artery disease	(Bego et al., 2011; Ben Ali et al., 2009; Bordoni et al., 2017; Bystrova et al., 2017; Chan et al., 2013; Dedoussis et al., 2009; DIAbetes Genetics Replication And Meta-analysis (DIAGRAM) Consortium et al., 2014; Ding et al., 2012; Estivalet et al., 2011; Gaulton et al., 2008; Gouda et al., 2010; Hindy et

			al., 2016; Hoffmann et al., 2018; Hsiao and Lin, 2015; Huang et al., 2016; Kilpeläinen et al., 2008; Li et al., 2015; Manning et al., 2012; Morris et al., 2012; Namvaran et al., 2011; Phani et al., 2016; Queiroz et al., 2015; Regieli et al., 2009; Sanghera et al., 2008, 2010; Saxena et al., 2007; Scott et al., 2007; Shi et al., 2012; Stancáková et al., 2009; Tan et al., 2014; Tellechea et al., 2009; Trombetta et al., 2013; Wang et al., 2015b; Wang and Liu, 2012; Wu et al., 2016; Zeggini et al., 2007; Zhao et al., 2017; Zhu et al., 2017a)
PPARGC1A **Peroxisome Proliferator Activated Receptor Gamma Coactivator 1-alpha**			
rs12640088	intron variant	type- 2 diabetes	(Villegas et al., 2014)
rs8192678	missense variant	type-2 diabetes, Non-alcoholic steatohepatitis, insulin resistance, BMI	(Deeb and Brunzell, 2009; Franks et al., 2014; Lin et al., 2013; Povel et al., 2010; Queiroz et al., 2015; Tai et al., 2016; Zhu et al., 2017a)
rs251464	intron variant	BMI, type-2 diabetes	(Villegas et al., 2014)
VDR **Vitamin D Receptor**			
rs17383291	intergenic variant	colorectal cancer	(Poynter et al., 2010)
rs2228570	start lost	BMI, insulin resistance, type-2 diabetes, triglycerides, HDL cholesterol, hypertension	(Jia et al., 2015; Koroglu et al., 2014; Li et al., 2014; Safar et al., 2018; Zhong et al., 2015a)
FOXO1 **Forkhead Box O1**			
rs10507486	intron variant	carotid atherosclerosis, type-2 diabetes	(Kedenko et al., 2014; Sookoian et al.,

			2009b)
rs2297627	intron variant	carotid atherosclerosis	(Kedenko et al., 2014; Muller et al., 2015; Müssig et al., 2009)
MTNR1B **Melatonin Receptor 1B**			
rs10830963	intron variant	gestational diabetes mellitus, type-2 diabetes, fasting glucose, hemoglobin A1C, insulin resistance, birth weight, obesity-related traits	(Barker et al., 2011; Beaumont et al., 2018; Chambers et al., 2009; Comuzzie et al., 2012; de Luis et al., 2018; DIAbetes Genetics Replication And Meta-analysis (DIAGRAM) Consortium et al., 2014; Dupuis et al., 2010; Evangelou et al., 2018; Garaulet et al., 2015; Goni et al., 2014; Holzapfel et al., 2011; Horikoshi et al., 2016; Hu et al., 2010; Huopio et al., 2013; Ingelsson et al., 2010; Kanai et al., 2018; Keaton et al., 2018; Kettunen et al., 2012; Kong et al., 2015; Kwak et al., 2012; Lane et al., 2016; Langenberg et al., 2009; Liao et al., 2012; Liu et al., 2010; Loomis et al., 2018; Lu et al., 2017; Mao et al., 2012; Morris et al., 2012; Nettleton et al., 2007; Ohshige et al., 2011; Palmer et al., 2015; Prokopenko et al., 2009, 2014; Qi et al., 2017; Rasmussen-Torvik et al., 2010; Rasmussen-Torvik et al., 2012; Reinehr et al., 2011; Ren et al., 2014; Renström et al., 2011, 2015; Rönn et al., 2009; Salman et al., 2015; Simonis-Bik et al., 2010; Sparsø et al., 2009; Staiger et al., 2008; Stuebe et al., 2014; Tam et al., 2010; Tarnowski et al., 2017; Wang et al., 2011b; Wheeler et al., 2017; Wood et al., 2017; Wu et al., 2016; Wu and Pankow, 2018; Xue et al., 2018; Zhang et al., 2014b; Zhao et al., 2014, 2017;

			Zheng et al., 2015)
LEPR **Leptin Receptor**			
rs1137101	missense variant	triglycerides, gestational diabetes mellitus, fasting glucose, BMI, cardiovascular disease, preterm birth, type-2 diabetes, plasma lipids	(Anghebem-Oliveira et al., 2017; Ben Ali et al., 2009; Furusawa et al., 2010; Gregoor et al., 2009; Kasim et al., 2016; Mahmoudi et al., 2016; Manriquez et al., 2018; Queiroz et al., 2015; Rojano-Rodriguez et al., 2016; Salem et al., 2016; Tabassum et al., 2012; Urbanek et al., 2012; Wu and Sun, 2017; Yang et al., 2016; Zayani et al., 2017)
HMGCR **3-Hydroxy-3-Methylglutaryl-CoA Reductase**			(Bream et al., 2013; Steffen et al., 2007)
rs2303152	intron variant	preterm delivery	
rs12654264	intron variant	HDL cholesterol, LDL cholesterol, triglycerides, fasting blood glucose	(Kathiresan et al., 2008; Keebler Mary E. et al., 2009; Kim et al., 2011)
DHCR24 **24-Dehydrocholesterol Reductase**			
rs2274941	non coding transcript exon variant	prematurity	(Steffen et al., 2007)
DHCR7 ***7*****-Dehydrocholesterol Reductase**			
rs1630498	intron variant	low birth weight, prematurity	(Bream et al., 2013; Steffen et al., 2007)
rs2002064	intron variant	birth weight	(Steffen et al., 2007)
PCSK9 **Proprotein Convertase**			

**Subtilisin/Kexin Type 9**			
rs11591147	missense variant	LDL cholesterol, total cholesterol, cardiovascular disease, fasting glucose, type-2 diabetes	(Chasman et al., 2012; Feng et al., 2017a; Guella et al., 2010; Guo et al., 2016; Kathiresan et al., 2007, 2008; Kettunen et al., 2012, 2016; Klarin et al., 2018; Nagy et al., 2017; Nelson et al., 2017; Pott et al., 2018; Qiu et al., 2017; Rasmussen-Torvik et al., 2012; Schmidt et al., 2017; Smith et al., 2010; Southam et al., 2017; Spracklen et al., 2017; Surakka et al., 2015; Tsai et al., 2015; van der Harst and Verweij, 2018)
CETP **Cholesteryl Ester Transfer Protein**			
rs1800775	regulatory region variant	HDL cholesterol, LDL cholesterol, total cholesterol, triglycerides, coronary artery disease, metabolic syndrome, dyslipidemia	(Andaleon et al., 2018; Barbosa et al., 2012; Boes et al., 2009; Chasman et al., 2009; Ganesan et al., 2016; Guo et al., 2015b; Hamrefors et al., 2010; Hebbar et al., 2017; Hou et al., 2017; Hu et al., 2016; Kathiresan et al., 2007, 2008; Kenny et al., 2011; Ma et al., 2010; Murray et al., 2009; Nie et al., 2017; Ridker et al., 2009; Ronald et al., 2009; Sabatti et al., 2009; Saxena et al., 2007, 200; Shin et al., 2014; Wakil et al., 2016; Webb et al., 2017; Winkler et al., 2015; Wu et al., 2013)
LCAT **Lecithin-Cholesterol Acyltransferase**			
rs1109166	3’ UTR	gestational age, HDL cholesterol	(Spracklen et al., 2017; Steffen et al., 2007)
LIPC **Lipase C, Hepatic type**			
rs6083	missense variant	gestational age, preterm prelabor rapture of	(Romero et al., 2010; Steffen et al., 2007;

		membranes, HDL cholesterol	Yang et al., 2010)
rs1800588	intron variant	total cholesterol, HDL cholesterol, triglyceride levels, LDL cholesterol, cardiovascular disease, hypertriglyceridemia	(Ahmad et al., 2011; Ayyappa et al., 2013; Fan et al., 2009; Guardiola et al., 2015; Hamrefors et al., 2010; Hodoglugil et al., 2010; Kanai et al., 2018; Kathiresan et al., 2008; Liu et al., 2011; Lu et al., 2008, 2016a; Nie et al., 2017; Ríos-González et al., 2014; Rudkowska et al., 2013; Sabatti et al., 2009; Spracklen et al., 2017; Villard et al., 2013; White et al., 2015; Yang et al., 2010)
LIPG **Lipase G, endothelial type**			
rs2156552	intergenic variant	HDL cholesterol, ischemic stroke	(Carty Cara L. et al., 2012; Gaulton et al., 2008; Jeemon et al., 2011; Kathiresan et al., 2008; Keebler Mary E. et al., 2009; Khetarpal et al., 2011; Klarin et al., 2018; Ma et al., 2010; Murray et al., 2009; Spracklen et al., 2017; Waterworth et al., 2010)
LPL **Lipoprotein Lipase**			
rs328	stop variant	HDL cholesterol, triglycerides, blood pressure, coronary heart disease	(Ayyappa et al., 2017; Chen et al., 2009; De Castro-Orós Isabel et al., 2014; Drenos et al., 2009; Dumitrescu et al., 2011; Emamian et al., 2015; Garcia-Rios et al., 2011; Guardiola et al., 2015; Jeemon et al., 2011; Kathiresan et al., 2007, 2008; Keebler Mary E. et al., 2009; Kurano et al., 2016; Larifla et al., 2016; Legry et al., 2011; Murray et al., 2009; Pirim et al., 2015; Ronald et al., 2009; Sabatti et al., 2009; Sagoo et al., 2008; Shahid et al., 2017; Tan et al., 2012; Tang et al., 2010; Webster et al., 2009;

			White et al., 2015; Yeo et al., 2017; Yue et al., 2017; Zhou et al., 2013)
ABCA1 **ATP Binding Cassette Subfamily A Member 1**			
rs2066716	missense variant	prematurity, gestational age, birth weight	(Steffen et al., 2007)
rs3890182	intron variant	HDL cholesterol, overweight, obesity	(Jeemon et al., 2011; Kathiresan et al., 2008; Kong et al., 2015; Sabatti et al., 2009; Waterworth et al., 2010; Yao et al., 2016a, 2016b)
rs4149313	missense variant	gestational age	(Steffen et al., 2007)
LDLR **Low Density Lipoprotein Receptor**			
rs6511720	intron variant	LDL cholesterol, total cholesterol, coronary artery disease, hypertension	(Chasman et al., 2008, 2009; Consortium, 2011; Ding and Kullo, 2009; Elbers et al., 2012; Feng et al., 2017b; Grallert et al., 2012; Gupta et al., 2010; Inouye et al., 2012; Kathiresan et al., 2008, 2009; Keebler Mary E. et al., 2009; Lettre et al., 2011; Linsel-Nitschke et al., 2010; Middelberg et al., 2011; Nelson et al., 2017; Rafiq et al., 2012; Ronald et al., 2009; Shetty Priya B. et al., 2015; Teslovich et al., 2010; van der Harst and Verweij, 2018; Willer et al., 2008; Ye et al., 2014)
APOB **Apolipoprotein B**			
rs693	synonymous variant	LDL cholesterol, HDL cholesterol, triglycerides, total cholesterol, ischemic stroke, coronary heart disease	(Au et al., 2017; Aulchenko et al., 2009; Chen et al., 2016; Haas Blake E. et al., 2011; Hamrefors et al., 2010; Hubacek et al., 2017; Kathiresan et al., 2008; Murray et al., 2009; Niu et al., 2017; Park et al., 2011; Rodrigues et al., 2013; Sabatti et al., 2009; Sandhu et al., 2008; Saxena et

			al., 2007; Shirts et al., 2011; Takeuchi et al., 2012; Walker et al., 2011; Xiao et al., 2017)
APOA1 **Apolipoprotein A1**			
rs28927680	3' UTR	triglycerides, total cholesterol	(Jeemon et al., 2011; Kathiresan et al., 2008; Sabatti et al., 2009)
rs5070	intron variant	prematurity, HDL cholesterol	(Rudkowska et al., 2013; Steffen et al., 2007)
APOE **Apolipoprotein**			
rs405509	regulatory region variant	prematurity, gestational age, birth weight, serum fasting insulin, overweight	(Clark et al., 2009; Edwards et al., 2011; Komurcu-Bayrak et al., 2011; Steffen et al., 2007)
rs7412	missense variant	gestational age, LDL cholesterol, total cholesterol, HDL cholesterol, coronary artery disease, premature coronary artery disease, dyslipidemia, type-2 diabetes	(Alharbi et al., 2014; Ansari et al., 2017; Barbosa et al., 2012; Cahua-Pablo et al., 2016; Chasman et al., 2012; Futema et al., 2015; Hanh et al., 2016; Mazzotti et al., 2014; Ripatti et al., 2016; Smith et al., 2010; Smolková et al., 2015; Steffen et al., 2007; Surakka et al., 2015; Takeuchi et al., 2012; Tejedor et al., 2014; van der Harst and Verweij, 2018; Wu et al., 2013; Zhu et al., 2017b)
APOC1 **Apolipoprotein C1**			
rs4420638	intergenic variant	LDL cholesterol, HDL cholesterol, total cholesterol, triglycerides, coronary artery disease, c-reactive protein, BMI, type-2 diabetes	(Adeyemo et al., 2012; Aslibekyan et al., 2012; Burkhardt et al., 2008; Burkhardt Ralph et al., 2008; Chung et al., 2014; Dehghan Abbas et al., 2011; Deshmukh et al., 2012; Elliott et al., 2009; Hubacek et al., 2017; Kathiresan et al., 2008; Keller et al., 2013; Kenny et al., 2011; Kim et al., 2017b; Li et al., 2017; Ligthart et al., 2016; Liu et al., 2011; Lu et al., 2016b; Middelberg et al., 2011;

			Mohlke et al., 2008; Nikpay et al., 2015; Okada et al., 2011; Park et al., 2011; Sandhu et al., 2008; Saxena et al., 2007; Shirali et al., 2016, 201; Shirts et al., 2011; Spracklen et al., 2017; Teslovich et al., 2010; Varga et al., 2014; Wallace et al., 2008; Waterworth et al., 2010; Willer et al., 2013; Winkler et al., 2015; Zhao et al., 2017)
PNPLA3 **Patatin Like Phospholipase Domain Containing 3**			
rs738409	missense variant	non-alcoholic fatty liver disease pathogenesis, total cholesterol, triglycerides, insulin resistance BMI, gestational diabetes, metabolic syndrome	(Akuta et al., 2016; Alam et al., 2017; Ali et al., 2016; Atkinson et al., 2017; Basantani et al., 2011; Bhatt et al., 2013; Bo et al., 2015; Buch et al., 2015; Burza et al., 2014; Cai et al., 2011; Chalasani et al., 2010; Chamorro et al., 2014; Chan et al., 2017; Cox et al., 2011; Falleti et al., 2016; Fan et al., 2016; Flores et al., 2016; Gao et al., 2017; Goran et al., 2010; Graff et al., 2013; Guichelaar et al., 2013; Guyot et al., 2013; Hassan et al., 2013; Hernaez et al., 2013; Hotta et al., 2010; Huang et al., 2015a, 3, 2017, 3; Jiménez-Sousa et al., 2016; Johansson et al., 2008; Kantartzis et al., 2009; Kawaguchi et al., 2012; Kim et al., 2018, 3; Kitamoto et al., 2015; Klarin et al., 2018; Kotronen et al., 2009; Kovac and Rozman, 2015; Krawczyk et al., 2011, 2017; Kupcinskas et al., 2017; Lee et al., 2014; Lin et al., 2011, 2014; Liu et al., 2014a; Mancina et al., 2016; Mangge et al., 2015; Miyaaki et al., 2018; Mondul et al., 2015; Moritou et al., 2013; Oniki

			et al., 2015; Pan et al., 2015; Petta et al., 2012, 2016; Pirazzi et al., 2012; Pontoriero et al., 2015; Rausch et al., 2016; Romeo et al., 2008, 2010a, 2010b; Rüeger et al., 2015; Salameh et al., 2016; Santoro et al., 2010; Sato et al., 2014; Scheiner et al., 2015; Seko et al., 2018; Sevastianova et al., 2011; Shang et al., 2015; Shen et al., 2014, 2015a; Singal et al., 2014; Smagris et al., 2015; Sookoian et al., 2009a; Sookoian and Pirola, 2016; Speliotes et al., 2010, 2011; Stickel et al., 2011; Stojkovic et al., 2014; Tai et al., 2015; Takeuchi et al., 2013; Tang et al., 2015; Trepo et al., 2012; Trépo et al., 2011a, 2011b, 2014; Ueyama et al., 2016; Uygun et al., 2017; Valenti et al., 2010a, 2010b, 2012; Verrijken et al., 2013; Vespasiani-Gentilucci et al., 2016; Viganò et al., 2013; Viitasalo et al., 2015; Wagenknecht et al., 2011; Wang et al., 2016; Xia et al., 2016; Xu et al., 2015; Yasui et al., 2015; Zain et al., 2012; Zhang et al., 2014a, 2015)
TM6SF2 **Transmembrane 6 Superfamily Member 2**			
rs58542926	missense variant	non-alcoholic fatty liver disease pathogenesis, triglycerides, lipid abnormalities, total cholesterol	(Boonvisut et al., 2016; Chen et al., 2015, 2; Dongiovanni et al., 2015; Ehrhardt et al., 2017; Eslam et al., 2016; Falleti et al., 2016; Goffredo et al., 2016; Kanai et al., 2018; Kanth et al., 2016; Kim et al., 2017a; Liu et al., 2014b; Petta et al., 2016; Pirola and Sookoian, 2015; Sookoian et al., 2015, 2016; Surakka et al., 2015; Tang et al., 2015; Wang et al.,

			2016)
NCAN **Neurocan** rs16996148	intergenic variant	LDL cholesterol, HDL cholesterol, triglycerides, coronary heart disease	(Kathiresan et al., 2008; Willer et al., 2008)
TRIB1 **G-Protein-Coupled Receptor-Induced Gene 2 Protein** rs17321515	intron variant	LDL cholesterol, HDL cholesterol, triglycerides, coronary heart disease	(Aung et al., 2011; Dastani et al., 2012; Hegele et al., 2009; Huang et al., 2016; Kathiresan et al., 2008; Keebler Mary E. et al., 2009; Mohlke et al., 2008; Ollila et al., 2012; Park et al., 2011; Wang et al., 2015a; Willer et al., 2008; Zhou et al., 2011, 2013)
ANGPTL3 **Angiopoietin Like 3** rs12130333	intergenic variant	hypertriglyceridemia, triglycerides	(Hegele et al., 2009; Kathiresan et al., 2008; Wang et al., 2008)
IGF2 **Insulin like growth factor 2** rs74050124	3’ UTR	pregnancy complications	(Queiroz et al., 2015, 2)

*rs74050124 and rs680 are in LD

Supplementary Table S3: p-values for the association between 2nd trimester lipid levels and SNPs in lipid and circadian genes.

**SNPs**	**Core circadian regulating genes**	**CHOL**	**HDL**	**LDL**	**TG**
rs3749474	*CLOCK*	0.86	5.37×10^{-2}	0.79	8.70×10^{-4}
rs4580704	*CLOCK*	0.31	1.93×10^{-3}	0.63	1.02×10^{-2}
rs1464490	*CLOCK*	0.85	4.94×10^{-2}	0.75	9.72×10^{-4}
rs6843722	*CLOCK*	0.73	8.07×10^{-3}	0.83	4.43×10^{-4}
rs6850524	*CLOCK*	0.44	2.27×10^{-4}	0.54	2.05×10^{-3}
rs4864548	*CLOCK*	0.89	4.14×10^{-2}	0.73	8.93×10^{-4}
rs1801260	*CLOCK*	0.42	0.40	9.28×10^{-2}	0.92
rs2278749	*ARNTL*	0.67	3.56×10^{-3}	0.82	1.50×10^{-3}
rs6486121	*ARNTL*	6.21×10^{-3}	0.93	6.42×10^{-3}	0.50
rs7950226	*ARNTL*	0.62	6.88×10^{-3}	0.85	0.21
rs11022775	*ARNTL*	0.56	0.25	0.30	6.35×10^{-2}
rs2585405	*PER1*	2.31×10^{-2}	0.80	1.06×10^{-2}	0.76
rs3027178	*PER1*	0.17	0.42	0.14	2.97×10^{-3}
rs2304672	*PER2*	0.65	0.45	0.69	0.99
rs56013859	*PER2*	0.11	6.04×10^{-2}	2.20×10^{-2}	5.08×10^{-2}
rs7602358	*PER3*	2.51×10^{-2}	1.72×10^{-3}	0.18	0.12

rs228669	*PER3*	0.50	0.19	0.81	1.24×10^{-6}
rs2640908	*PER3*	3.77×10^{-4}	0.28	1.78×10^{-4}	0.64
rs2287161	*CRY1*	0.53	0.95	0.81	0.95
rs3809236	*CRY1*	0.69	0.52	0.52	3.56×10^{-2}
rs12315175	*CRY1*	9.28×10^{-2}	0.38	0.41	0.15
rs2292912	*CRY2*	4.84×10^{-2}	0.85	3.92×10^{-3}	0.25
rs11605924	*CRY2*	N/A	N/A	N/A	N/A
rs2305160	*NPAS2*	0.21	0.23	0.11	5.46×10^{-2}
rs11541353	*NPAS2*	0.84	0.30	0.22	4.03×10^{-2}
**SNPs**	**Circadian-related and lipid-related genes**	**CHOL**	**HDL**	**LDL**	**TG**
rs12413112	*SIRT1*	0.79	6.17×10^{-2}	0.96	5.25×10^{-3}
rs3758391	*SIRT1*	1.83×10^{-2}	0.39	7.73×10^{-3}	3.84×10^{-2}
rs2273773	*SIRT1*	7.22×10^{-2}	0.78	5.10×10^{-2}	0.40
rs10997860	*SIRT1*	N/A	N/A	N/A	N/A
rs646776	*CELSR2-PSRC1-SORT1*	2.35×10^{-5}	5.35×10^{-4}	5.10×10^{-11}	0.19
rs599839	*CELSR2-PSRC1-SORT1*	2.83×10^{-5}	7.10×10^{-4}	1.16×10^{-10}	5.38×10^{-2}
rs28932472	*POMC*	0.13	0.38	0.65	0.39
rs1800206	*PPARA*	0.28	0.49	0.61	4.35×10^{-3}

rs7638903	*PPARG*	0.92	0.16	0.88	0.29
rs1801282	*PPARG*	0.95	0.60	0.94	0.45
rs12640088	*PPARGC1A*	0.39	0.31	0.16	1.10×10^{-2}
rs8192678	*PPARGC1A*	0.78	0.12	0.35	0.82
rs251464	*PPARGC1B*	0.24	0.95	3.30×10^{-2}	4.40×10^{-2}
rs17383291	*VDR*	0.45	0.21	0.56	4.17×10^{-2}
rs2228570	*VDR*	0.58	2.87×10^{-2}	0.44	4.66×10^{-2}
rs10507486	*FOXO1*	0.55	0.47	0.25	0.15
rs2297627	*FOXO1*	0.27	0.87	0.16	0.10
rs10830963	*MTNR1B*	0.69	0.86	0.76	0.56
rs1137101	*LEPR*	0.10	0.39	0.44	0.70
rs2303152	*HMGCR*	0.56	0.20	0.24	0.51
rs12654264	*HMGCR*	N/A	N/A	N/A	N/A
rs2274941	*DHCR24*	N/A	N/A	N/A	N/A
rs1630498	*DHCR7*	0.73	0.92	0.60	0.57
rs2002064	*DHCR7*	0.86	0.92	0.65	0.68
rs11591147	*PCSK9*	0.47	0.27	0.98	0.75
rs1800775	*CETP*	0.10	0.12	0.54	4.27×10^{-2}

rs1109166	*LCAT*	0.99	3.15×10^{-2}	0.85	0.28
rs6083	*LIPC*	8.86×10^{-2}	0.74	2.75×10^{-2}	0.80
rs1800588	*LIPC*	0.22	5.57×10^{-2}	7.63×10^{-3}	2.16×10^{-4}
rs2156552	*LIPG*	0.25	0.63	0.34	0.12
rs328	*LPL*	6.65×10^{-3}	5.52×10^{-2}	1.13×10^{-2}	1.33×10^{-3}
rs2066716	*ABCA1*	0.13	0.996	0.16	1.81×10^{-4}
rs3890182	*ABCA1*	0.69	0.73	0.32	1.63×10^{-2}
rs4149313	*ABCA1*	0.18	0.42	2.74×10^{-3}	0.49
rs6511720	*LDLR*	0.92	0.55	0.92	0.35
rs693	*APOB*	8.46×10^{-2}	0.83	3.70×10^{-3}	0.70
rs28927680	*APOA1*	0.44	0.13	0.18	6.66×10^{-3}
rs5070	*APOA1*	0.35	0.92	4.83×10^{-2}	1.24×10^{-3}
rs405509	*APOE*	3.39×10^{-3}	6.94×10^{-2}	1.63×10^{-3}	0.31
rs7412	*APOE*	2.81×10^{-6}	6.35×10^{-5}	$< 1.0 \times 10^{-12}$	0.10
rs4420638	*APOC1*	0.14	0.52	9.52×10^{-3}	0.23
rs738409	*PNPLA3*	0.33	9.81×10^{-5}	0.54	3.56×10^{-6}
rs58542926	*TM6SF2*	0.12	0.89	4.22×10^{-2}	0.89
rs16996148	*NCAN*	0.76	0.49	0.81	0.38

rs17321515	*TRIB1*	0.55	6.69×10^{-3}	0.89	0.26
rs12130333	*ANGPTL3*	0.71	0.18	0.33	9.71×10^{-2}
rs74050124	*IGF2*	0.91	3.38×10^{-2}	0.91	4.73×10^{-2}

All data presented are unadjusted and represent the p-value for the association between each individual lipid (as the outcome) with a single candidate SNP.

Supplementary Table S4: Minor allele frequencies for candidate SNPs and differences in frequency by race.

			**Minor Allele Frequency**				
	**SNPs**	**Minor Allele**	**Overall**	**Hispanic**	**White Non-Hispanic**	**Asian**	**p-value**
ABCA1	rs2066716	A	0.17	0.16	0.18	0.18	0.57
ABCA1	rs3890182	A	0.09	0.10	0.08	0.11	0.41
ABCA1	rs4149313	G	0.26	0.25	0.30	0.18	0.003
ANGPTL3	rs12130333	T	0.20	0.19	0.19	0.23	0.40
APOA1	rs28927680	G	0.10	0.09	0.11	0.09	0.35
APOA1	rs5070	A	0.47	0.47	0.49	0.44	0.45
APOB	rs693	T	0.38	0.38	0.37	0.40	0.76
APOC1	rs4420638	G	0.13	0.14	0.13	0.10	0.34
APOE	rs405509	C	0.47	0.46	0.46	0.47	0.96
APOE	rs7412	T	0.05	0.04	0.06	0.04	0.24
ARNTL	rs2278749	A	0.22	0.25	0.18	0.22	0.02
ARNTL	rs6486121	T*	0.49	0.49	0.49	0.47	0.59
ARNTL	rs7950226	G*	0.47	0.45	0.50	0.49	0.08
ARNTL	rs11022775	T	0.11	0.10	0.12	0.10	0.53
CELSR2-PSRC1-SORT1	rs646776	G	0.19	0.18	0.18	0.22	0.31
CELSR2-PSRC1-SORT1	rs599839	T	0.21	0.20	0.21	0.25	0.34
CETP	rs1800775	C	0.47	0.45	0.48	0.46	0.68
CLOCK	rs3749474	T	0.45	0.47	0.43	0.43	0.18
CLOCK	rs4580704	G	0.31	0.31	0.33	0.32	0.58
CLOCK	rs1464490	C	0.45	0.47	0.43	0.43	0.18
CLOCK	rs6843722	C	0.43	0.44	0.41	0.41	0.30
CLOCK	rs6850524	C	0.36	0.35	0.39	0.38	0.34

CLOCK	rs4864548	A	0.45	0.47	0.42	0.43	0.15
CLOCK	rs1801260	C	0.21	0.21	0.22	0.23	0.73
CRY1	rs3809236	T	0.12	0.12	0.11	0.13	0.73
CRY1	rs12315175	C	0.19	0.21	0.18	0.16	0.22
CRY1	rs2287161	C	0.45	0.44	0.47	0.42	0.26
CRY2	rs2292912	C	0.34	0.33	0.36	0.30	0.25
DHCR7	rs1630498	G	0.24	0.25	0.23	0.25	0.81
DHCR7	rs2002064	C	0.25	0.25	0.24	0.25	0.85
FOXO1	rs10507486	T	0.21	0.21	0.20	0.23	0.54
FOXO1	rs2297627	C	0.43	0.44	0.43	0.38	0.30
HMGCR	rs2303152	T	0.07	0.07	0.07	0.12	0.03
IGF2	rs74050124	A	0.01	0.01	0.01	0.01	0.86
LCAT	rs1109166	G	0.18	0.18	0.17	0.19	0.73
LDLR	rs6511720	T	0.09	0.09	0.09	0.11	0.59
LEPR	rs1137101	A*	0.48	0.47	0.47	0.44	0.06
LIPC	rs6083	A*	0.49	0.50	0.45	0.44	0.02
LIPC	rs1800588	T	0.40	0.40	0.42	0.36	0.26
LIPG	rs2156552	A	0.12	0.11	0.13	0.12	0.33
LPL	rs328	G	0.09	0.09	0.09	0.07	0.68
MTNR1B	rs10830963	G	0.31	0.32	0.30	0.30	0.59
NCAN	rs16996148	T	0.08	0.07	0.09	0.09	0.18
NPAS2	rs2305160	T	0.27	0.26	0.27	0.30	0.49
NPAS2	rs11541353	A	0.11	0.11	0.11	0.11	0.98
PCSK9	rs11591147	T	0.007	0.005	0.008	0.01	0.67
PER1	rs2585405	C	0.19	0.20	0.19	0.15	0.32
PER1	rs3027178	C	0.39	0.38	0.42	0.35	0.20
PER2	rs2304672	C	0.05	0.05	0.05	0.07	0.29

PER2	rs56013859	C	0.14	0.14	0.13	0.16	0.62
PER2	rs7602358	G	0.18	0.17	0.19	0.21	0.22
PER3	rs228669	A	0.21	0.21	0.23	0.11	0.003
PER3	rs2640908	T	0.22	0.22	0.24	0.20	0.57
PNPLA3	rs738409	G	0.37	0.38	0.33	0.40	0.08
POMC	rs28932472	C	0.21	0.22	0.20	0.20	0.48
PPARA	rs1800206	G	0.04	0.05	0.04	0.03	0.40
PPARG	rs1801282	G	0.10	0.11	0.09	0.08	0.35
PPARG	rs7638903	A	0.10	0.11	0.09	0.09	0.29
PPARGC1A	rs8192678	A	0.31	0.32	0.31	0.34	0.75
PPARGC1A	rs12640088	C	0.10	0.10	0.10	0.09	0.78
PPARGC1B	rs251464	C	0.36	0.36	0.36	0.40	0.52
SIRT1	rs12413112	A	0.16	0.16	0.16	0.16	0.99
SIRT1	rs3758391	T*	0.50	0.49	0.49	0.49	0.89
SIRT1	rs2273773	C	0.14	0.14	0.14	0.12	0.67
TM6SF2	rs58542926	T	0.06	0.05	0.07	0.06	0.39
TRIB1	rs17321515	G	0.44	0.43	0.47	0.43	0.23
VDR	rs17383291	G	0.43	0.42	0.43	0.43	0.91
VDR	rs2228570	A	0.44	0.45	0.44	0.34	0.02

*Minor allele for rs6486121 in White-Not Hispanic is C; Minor allele for rs7950226 in White-Not Hispanic is A; Minor allele for rs11605924 in Hispanic is A; Minor allele for rs3758391 in Hispanic is C; Minor allele for rs10997860 in White-Not Hispanic is T; Minor allele for rs1137101 in Asian is G; Minor allele for rs6083 in Asian is G.

MAF, minor allele frequency as calculated from the study population.

P-value represents the chi-square test for differences in MAF by race.

References

Abe, S., Tokoro, F., Matsuoka, R., Arai, M., Noda, T., Watanabe, S., et al. (2015). Association of genetic variants with dyslipidemia. *Mol. Med. Rep.* 12, 5429–5436. doi:10.3892/mmr.2015.4081.

Adeyemo, A., Bentley, A. R., Meilleur, K. G., Doumatey, A. P., Chen, G., Zhou, J., et al. (2012). Transferability and Fine Mapping of genome-wide associated loci for lipids in African Americans. *BMC Med. Genet.* 13, 88. doi:10.1186/1471-2350-13-88.

Ahmad, T., Chasman, D. I., Buring, J. E., Lee, I.-M., Ridker, P. M., and Everett, B. M. (2011). Physical activity modifies the effect of LPL, LIPC, and CETP polymorphisms on HDL-C levels and the risk of myocardial infarction in women of European ancestry. *Circ. Cardiovasc. Genet.* 4, 74–80. doi:10.1161/CIRCGENETICS.110.957290.

Akuta, N., Kawamura, Y., Arase, Y., Suzuki, F., Sezaki, H., Hosaka, T., et al. (2016). Relationships between Genetic Variations of PNPLA3, TM6SF2 and Histological Features of Nonalcoholic Fatty Liver Disease in Japan. *Gut Liver* 10, 437–445. doi:10.5009/gnl15163.

Alam, S., Islam, M. S., Islam, S., Mustafa, G., Saleh, A. A., and Ahmad, N. (2017). Association of single nucleotide polymorphism at PNPLA3 with fatty liver, steatohepatitis, and cirrhosis of liver. *Indian J. Gastroenterol.* 36, 366–372. doi:10.1007/s12664-017-0784-y.

Alharbi, K. K., Khan, I. A., and Syed, R. (2014). Association of apolipoprotein E polymorphism with type 2 diabetes mellitus in a Saudi population. *DNA Cell Biol.* 33, 637–641. doi:10.1089/dna.2014.2461.

Ali, M., Yopp, A., Gopal, P., Beg, M. S., Zhu, H., Lee, W., et al. (2016). A Variant in PNPLA3 Associated With Fibrosis Progression but not Hepatocellular Carcinoma in Patients With Hepatitis C Virus Infection. *Clin. Gastroenterol. Hepatol. Off. Clin. Pract. J. Am. Gastroenterol. Assoc.* 14, 295–300. doi:10.1016/j.cgh.2015.08.018.

Alsaleh, A., Frost, G. S., Griffin, B. A., Lovegrove, J. A., Jebb, S. A., Sanders, T. A. B., et al. (2011). PPARγ2 gene Pro12Ala and PPARα gene Leu162Val single nucleotide polymorphisms interact with dietary intake of fat in determination of plasma lipid concentrations. *J. Nutr. Nutr.* 4, 354–366. doi:10.1159/000336362.

Andaleon, A., Mogil, L. S., and Wheeler, H. E. (2018). Gene-based association study for lipid traits in diverse cohorts implicates BACE1 and SIDT2 regulation in triglyceride levels. *PeerJ* 6, e4314. doi:10.7717/peerj.4314.

Andrulionytė, L., Kuulasmaa, T., Chiasson, J.-L., and Laakso, M. (2007). Single Nucleotide Polymorphisms of the Peroxisome Proliferator–Activated Receptor-α Gene (PPARA) Influence the Conversion From Impaired Glucose Tolerance to Type 2 Diabetes : The STOP-NIDDM Trial. *Diabetes* 56, 1181–1186. doi:10.2337/db06-1110.

Angelakopoulou, A., Shah, T., Sofat, R., Shah, S., Berry, D. J., Cooper, J., et al. (2012). Comparative analysis of genome-wide association studies signals for lipids, diabetes, and coronary heart disease: Cardiovascular Biomarker Genetics Collaboration. *Eur. Heart J.* 33, 393–407. doi:10.1093/eurheartj/ehr225.

Anghebem-Oliveira, M. I., Martins, B. R., Alberton, D., Ramos, E. A. de S., Picheth, G., and Rego, F. G. de M. (2017). Type 2 diabetes-associated genetic variants of FTO, LEPR, PPARg, and TCF7L2 in gestational diabetes in a Brazilian population. *Arch. Endocrinol. Metab.* 61, 238–248. doi:10.1590/2359-3997000000258.

Ansari, W. M., Humphries, S. E., Naveed, A. K., Khan, O. J., Khan, D. A., and Khattak, E. H. (2017). Effect of Coronary Artery Disease risk SNPs on serum cytokine levels and cytokine imbalance in Premature Coronary Artery Disease. *Cytokine*. doi:10.1016/j.cyto.2017.05.013.

Arvind, P., Nair, J., Jambunathan, S., Kakkar, V. V., and Shanker, J. (2014). CELSR2–PSRC1–SORT1 gene expression and association with coronary artery disease and plasma lipid levels in an Asian Indian cohort. *J. Cardiol.* 64, 339–346. doi:10.1016/j.jjcc.2014.02.012.

Aslibekyan, S., Goodarzi, M. O., Frazier-Wood, A. C., Yan, X., Irvin, M. R., Kim, E., et al. (2012). Variants identified in a GWAS meta-analysis for blood lipids are associated with the lipid response to fenofibrate. *PloS One* 7, e48663. doi:10.1371/journal.pone.0048663.

Atkinson, S. R., Way, M. J., McQuillin, A., Morgan, M. Y., and Thursz, M. R. (2017). Homozygosity for rs738409:G in PNPLA3 is associated with increased mortality following an episode of severe alcoholic hepatitis. *J. Hepatol.* 67, 120–127. doi:10.1016/j.jhep.2017.01.018.

Au, A., Griffiths, L. R., Irene, L., Kooi, C. W., and Wei, L. K. (2017). The impact of APOA5, APOB, APOC3 and ABCA1 gene polymorphisms on ischemic stroke: Evidence from a meta-analysis. *Atherosclerosis* 265, 60–70. doi:10.1016/j.atherosclerosis.2017.08.003.

Aulchenko, Y. S., Ripatti, S., Lindqvist, I., Boomsma, D., Heid, I. M., Pramstaller, P. P., et al. (2009). Loci influencing lipid levels and coronary heart disease risk in 16 European population cohorts. *Nat. Genet.* 41, 47–55. doi:10.1038/ng.269.

Aung, L. H. H., Yin, R.-X., Wu, D.-F., Li, Q., Yan, T.-T., Wang, Y.-M., et al. (2011). Association of the TRIB1 tribbles homolog 1 gene rs17321515 A>G polymorphism and serum lipid levels in the Mulao and Han populations. *Lipids Health Dis.* 10, 230. doi:10.1186/1476-511X-10-230.

Ayyappa, K. A., Ghosh, S., Mohan, V., and Radha, V. (2013). Association of hepatic lipase gene polymorphisms with hypertriglyceridemia and low high-density lipoprotein-cholesterol levels among South Indian subjects without diabetes. *Diabetes Technol. Ther.* 15, 503–512. doi:10.1089/dia.2012.0302.

Ayyappa, K. A., Shatwan, I., Bodhini, D., Bramwell, L. R., Ramya, K., Sudha, V., et al. (2017). High fat diet modifies the association of lipoprotein lipase gene polymorphism with high density lipoprotein cholesterol in an Asian Indian population. *Nutr. Metab.* 14, 8. doi:10.1186/s12986-016-0155-1.

Bandín, C., Martinez-Nicolas, A., Ordovás, J. M., Ros Lucas, J. A., Castell, P., Silvente, T., et al. (2013). Differences in circadian rhythmicity in CLOCK 3111T/C genetic variants in moderate obese women as assessed by thermometry, actimetry and body position. *Int. J. Obes. 2005* 37, 1044–1050. doi:10.1038/ijo.2012.180.

Barbosa, E. J. L., Glad, C. A. M., Nilsson, A. G., Filipsson Nyström, H., Götherström, G., Svensson, P.-A., et al. (2012). Genotypes associated with lipid metabolism contribute to differences in serum lipid profile of GH-deficient adults before and after GH replacement therapy. *Eur. J. Endocrinol.* 167, 353–362. doi:10.1530/EJE-12-0263.

Barker, A., Sharp, S. J., Timpson, N. J., Bouatia-Naji, N., Warrington, N. M., Kanoni, S., et al. (2011). Association of Genetic

Loci With Glucose Levels in Childhood and Adolescence: A Meta-Analysis of Over 6,000 Children. *Diabetes* 60, 1805–1812. doi:10.2337/db10-1575.

Basantani, M. K., Sitnick, M. T., Cai, L., Brenner, D. S., Gardner, N. P., Li, J. Z., et al. (2011). Pnpla3/Adiponutrin deficiency in mice does not contribute to fatty liver disease or metabolic syndrome. *J. Lipid Res.* 52, 318–329. doi:10.1194/jlr.M011205.

Beaumont, R. N., Warrington, N. M., Cavadino, A., Tyrrell, J., Nodzenski, M., Horikoshi, M., et al. (2018). Genome-wide association study of offspring birth weight in 86 577 women identifies five novel loci and highlights maternal genetic effects that are independent of fetal genetics. *Hum. Mol. Genet.* 27, 742–756. doi:10.1093/hmg/ddx429.

Bego, T., Dujic, T., Mlinar, B., Semiz, S., Malenica, M., Prnjavorac, B., et al. (2011). Association of PPARG and LPIN1 gene polymorphisms with metabolic syndrome and type 2 diabetes. *Med. Glas. Off. Publ. Med. Assoc. Zenica-Doboj Cant. Bosnia Herzeg.* 8, 76–83.

Ben Ali, S., Ben Yahia, F., Sediri, Y., Kallel, A., Ftouhi, B., Feki, M., et al. (2009). Gender-specific effect of Pro12Ala polymorphism in peroxisome proliferator-activated receptor γ-2 gene on obesity risk and leptin levels in a Tunisian population. *Clin. Biochem.* 42, 1642–1647. doi:10.1016/j.clinbiochem.2009.08.019.

Benyamin, B., Esko, T., Ried, J. S., Radhakrishnan, A., Vermeulen, S. H., Traglia, M., et al. (2014). Novel loci affecting iron homeostasis and their effects in individuals at risk for hemochromatosis. *Nat. Commun.* 5, 4926. doi:10.1038/ncomms5926.

Berg, S. W. van den, Dollé, M. E. T., Imholz, S., A, D. L. van der, Slot, R. van 't, Wijmenga, C., et al. (2009). Genetic variations in regulatory pathways of fatty acid and glucose metabolism are associated with obesity phenotypes: a population-based cohort study. *Int. J. Obes.* 33, 1143. doi:10.1038/ijo.2009.152.

Bhatt, S. P., Nigam, P., Misra, A., Guleria, R., Pandey, R. M., and Pasha, M. A. Q. (2013). Genetic variation in the patatin-like phospholipase domain-containing protein-3 (PNPLA-3) gene in Asian Indians with nonalcoholic fatty liver disease. *Metab. Syndr. Relat. Disord.* 11, 329–335. doi:10.1089/met.2012.0064.

Bo, S., Gambino, R., Menato, G., Canil, S., Ponzo, V., Pinach, S., et al. (2015). Isoleucine-to-methionine substitution at residue 148 variant of PNPLA3 gene and metabolic outcomes in gestational diabetes. *Am. J. Clin. Nutr.* 101, 310–318. doi:10.3945/ajcn.114.095125.

Boes, E., Coassin, S., Kollerits, B., Heid, I. M., and Kronenberg, F. (2009). Genetic-epidemiological evidence on genes associated with HDL cholesterol levels: A systematic in-depth review. *Exp. Gerontol.* 44, 136–160. doi:10.1016/j.exger.2008.11.003.

Boonvisut, S., Nakayama, K., Makishima, S., Watanabe, K., Miyashita, H., Lkhagvasuren, M., et al. (2016). Replication analysis of genetic association of the NCAN-CILP2 region with plasma lipid levels and non-alcoholic fatty liver disease in Asian and Pacific ethnic groups. *Lipids Health Dis.* 15, 8. doi:10.1186/s12944-016-0181-z.

Bordoni, L., Marchegiani, F., Piangerelli, M., Napolioni, V., and Gabbianelli, R. (2017). Obesity-related genetic polymorphisms and adiposity indices in a young Italian population. *IUBMB Life* 69, 98–105. doi:10.1002/iub.1596.

Bouchard-Mercier, A., Godin, G., Lamarche, B., Pérusse, L., and Vohl, M.-C. (2011). Effects of peroxisome proliferator-activated receptors, dietary fat intakes and gene-diet interactions on peak particle diameters of low-density lipoproteins. *J. Nutr. Nutr.*

4, 36–48. doi:10.1159/000324531.

Bream, E. N. A., Leppellere, C. R., Cooper, M. E., Dagle, J. M., Merrill, D. C., Christensen, K., et al. (2013). Candidate gene linkage approach to identify DNA variants that predispose to preterm birth. *Pediatr. Res.* 73, 135–141. doi:10.1038/pr.2012.166.

Breitling, C., Gross, A., Büttner, P., Weise, S., Schleinitz, D., Kiess, W., et al. (2015). Genetic Contribution of Variants near SORT1 and APOE on LDL Cholesterol Independent of Obesity in Children. *PLoS ONE* 10. doi:10.1371/journal.pone.0138064.

Buch, S., Stickel, F., Trépo, E., Way, M., Herrmann, A., Nischalke, H. D., et al. (2015). A genome-wide association study confirms PNPLA3 and identifies TM6SF2 and MBOAT7 as risk loci for alcohol-related cirrhosis. *Nat. Genet.* 47, 1443–1448. doi:10.1038/ng.3417.

Burkhardt, R., Kenny, E. E., Lowe, J. K., Birkeland, A., Josowitz, R., Noel, M., et al. (2008). Common SNPs in HMGCR in micronesians and whites associated with LDL-cholesterol levels affect alternative splicing of exon13. *Arterioscler. Thromb. Vasc. Biol.* 28, 2078–2084. doi:10.1161/ATVBAHA.108.172288.

Burkhardt Ralph, Kenny Eimear E., Lowe Jennifer K., Birkeland Andrew, Josowitz Rebecca, Noel Martha, et al. (2008). Common SNPs in HMGCR in Micronesians and Whites Associated With LDL-Cholesterol Levels Affect Alternative Splicing of Exon13. *Arterioscler. Thromb. Vasc. Biol.* 28, 2078–2084. doi:10.1161/ATVBAHA.108.172288.

Burza, M. A., Molinaro, A., Attilia, M. L., Rotondo, C., Attilia, F., Ceccanti, M., et al. (2014). PNPLA3 I148M (rs738409) genetic variant and age at onset of at-risk alcohol consumption are independent risk factors for alcoholic cirrhosis. *Liver Int. Off. J. Int. Assoc. Study Liver* 34, 514–520. doi:10.1111/liv.12310.

Bystrova, A. A., Ulitina, A. S., Kim, M. V., Skoryukova, S. A., Miroshnikova, V. V., Panteleeva, A. A., et al. (2017). [Genetic Risk Factors of Macrovascular Complications in Patients With Type 2 Diabetes]. *Kardiologiia* 57, 17–22.

Cahua-Pablo, G., Cruz, M., Moral-Hernández, O. D., Leyva-Vázquez, M. A., Antúnez-Ortiz, D. L., Cahua-Pablo, J. A., et al. (2016). Elevated Levels of LDL-C are Associated With ApoE4 but Not With the rs688 Polymorphism in the LDLR Gene. *Clin. Appl. Thromb. Off. J. Int. Acad. Clin. Appl. Thromb.* 22, 465–470. doi:10.1177/1076029614568714.

Cai, T., Dufour, J.-F., Muellhaupt, B., Gerlach, T., Heim, M., Moradpour, D., et al. (2011). Viral genotype-specific role of PNPLA3, PPARG, MTTP, and IL28B in hepatitis C virus-associated steatosis. *J. Hepatol.* 55, 529–535. doi:10.1016/j.jhep.2010.12.020.

Carty Cara L., Bůžková Petra, Fornage Myriam, Franceschini Nora, Cole Shelley, Heiss Gerardo, et al. (2012). Associations Between Incident Ischemic Stroke Events and Stroke and Cardiovascular Disease-Related Genome-Wide Association Studies Single Nucleotide Polymorphisms in the Population Architecture Using Genomics and Epidemiology Study. *Circ. Cardiovasc. Genet.* 5, 210–216. doi:10.1161/CIRCGENETICS.111.962191.

Chalasani, N., Guo, X., Loomba, R., Goodarzi, M. O., Haritunians, T., Kwon, S., et al. (2010). Genome-wide association study identifies variants associated with histologic features of nonalcoholic Fatty liver disease. *Gastroenterology* 139, 1567–1576, 1576.e1–6. doi:10.1053/j.gastro.2010.07.057.

Chambers, J. C., Zhang, W., Zabaneh, D., Sehmi, J., Jain, P., McCarthy, M. I., et al. (2009). Common genetic variation near

melatonin receptor MTNR1B contributes to raised plasma glucose and increased risk of type 2 diabetes among Indian Asians and European Caucasians. *Diabetes* 58, 2703–2708. doi:10.2337/db08-1805.

Chamorro, A.-J., Torres, J.-L., Mirón-Canelo, J.-A., González-Sarmiento, R., Laso, F.-J., and Marcos, M. (2014). Systematic review with meta-analysis: the I148M variant of patatin-like phospholipase domain-containing 3 gene (PNPLA3) is significantly associated with alcoholic liver cirrhosis. *Aliment. Pharmacol. Ther.* 40, 571–581. doi:10.1111/apt.12890.

Chan, A. W. H., Wong, G. L. H., Chan, H.-Y., Tong, J. H. M., Yu, Y.-H., Choi, P. C. L., et al. (2017). Concurrent fatty liver increases risk of hepatocellular carcinoma among patients with chronic hepatitis B. *J. Gastroenterol. Hepatol.* 32, 667–676. doi:10.1111/jgh.13536.

Chan, K. H. K., Niu, T., Ma, Y., You, N. Y., Song, Y., Sobel, E. M., et al. (2013). Common genetic variants in peroxisome proliferator-activated receptor-γ (PPARG) and type 2 diabetes risk among Women's Health Initiative postmenopausal women. *J. Clin. Endocrinol. Metab.* 98, E600-604. doi:10.1210/jc.2012-3644.

Chasman, D. I., Giulianini, F., MacFadyen, J., Barratt, B. J., Nyberg, F., and Ridker, P. M. (2012). Genetic determinants of statin-induced low-density lipoprotein cholesterol reduction: the Justification for the Use of Statins in Prevention: an Intervention Trial Evaluating Rosuvastatin (JUPITER) trial. *Circ. Cardiovasc. Genet.* 5, 257–264. doi:10.1161/CIRCGENETICS.111.961144.

Chasman, D. I., Paré, G., Mora, S., Hopewell, J. C., Peloso, G., Clarke, R., et al. (2009). Forty-three loci associated with plasma lipoprotein size, concentration, and cholesterol content in genome-wide analysis. *PLoS Genet.* 5, e1000730. doi:10.1371/journal.pgen.1000730.

Chasman, D. I., Paré, G., Zee, R. Y. L., Parker, A. N., Cook, N. R., Buring, J. E., et al. (2008). Genetic loci associated with plasma concentration of low-density lipoprotein cholesterol, high-density lipoprotein cholesterol, triglycerides, apolipoprotein A1, and Apolipoprotein B among 6382 white women in genome-wide analysis with replication. *Circ. Cardiovasc. Genet.* 1, 21–30. doi:10.1161/CIRCGENETICS.108.773168.

Chen, L.-Z., Xia, H. H.-X., Xin, Y.-N., Lin, Z.-H., and Xuan, S.-Y. (2015). TM6SF2 E167K Variant, a Novel Genetic Susceptibility Variant, Contributing to Nonalcoholic Fatty Liver Disease. *J. Clin. Transl. Hepatol.* 3, 265–270. doi:10.14218/JCTH.2015.00023.

Chen, S. N., Cilingiroglu, M., Todd, J., Lombardi, R., Willerson, J. T., Gotto, A. M., et al. (2009). Candidate genetic analysis of plasma high-density lipoprotein-cholesterol and severity of coronary atherosclerosis. *BMC Med. Genet.* 10, 111. doi:10.1186/1471-2350-10-111.

Chen, Y., Lin, M., Liang, Y., Zhang, N., and Rao, S. (2016). Association Between Apolipoprotein B XbaI Polymorphism and Coronary Heart Disease in Han Chinese Population: A Meta-Analysis. *Genet. Test. Mol. Biomark.* 20, 304–311. doi:10.1089/gtmb.2015.0126.

Cho, Y. S., Go, M. J., Kim, Y. J., Heo, J. Y., Oh, J. H., Ban, H.-J., et al. (2009). A large-scale genome-wide association study of Asian populations uncovers genetic factors influencing eight quantitative traits. *Nat. Genet.* 41, 527–534. doi:10.1038/ng.357.

Chu, L. W., Zhu, Y., Yu, K., Zheng, T., Yu, H., Zhang, Y., et al. (2008). Variants in circadian genes and prostate cancer risk: a

population-based study in China. *Prostate Cancer Prostatic Dis.* 11, 342–348. doi:10.1038/sj.pcan.4501024.

Chung, S.-K., Yu, H., Park, A. Y., Kim, J. Y., and Cha, S. (2014). Genetic loci associated with changes in lipid levels leading to constitution-based discrepancy in Koreans. *BMC Complement. Altern. Med.* 14, 230. doi:10.1186/1472-6882-14-230.

Clark, D., Skrobot, O. A., Adebiyi, I., Susce, M. T., de Leon, J., Blakemore, A. F., et al. (2009). Apolipoprotein-E gene variants associated with cardiovascular risk factors in antipsychotic recipients. *Eur. Psychiatry* 24, 456–463. doi:10.1016/j.eurpsy.2009.03.003.

Clark, S. J., Falchi, M., Olsson, B., Jacobson, P., Cauchi, S., Balkau, B., et al. (2012). Association of Sirtuin 1 (SIRT1) Gene SNPs and Transcript Expression Levels With Severe Obesity. *Obes. Silver Spring Md* 20, 178–185. doi:10.1038/oby.2011.200.

Claussnitzer, M., Dankel, S. N., Klocke, B., Grallert, H., Glunk, V., Berulava, T., et al. (2014). Leveraging Cross-Species Transcription Factor Binding Site Patterns: From Diabetes Risk Loci to Disease Mechanisms. *Cell* 156, 343–358. doi:10.1016/j.cell.2013.10.058.

Comuzzie, A. G., Cole, S. A., Laston, S. L., Voruganti, V. S., Haack, K., Gibbs, R. A., et al. (2012). Novel Genetic Loci Identified for the Pathophysiology of Childhood Obesity in the Hispanic Population. *PLOS ONE* 7, e51954. doi:10.1371/journal.pone.0051954.

Consortium, T. I. 50K C. (2011). Large-Scale Gene-Centric Analysis Identifies Novel Variants for Coronary Artery Disease. *PLOS Genet.* 7, e1002260. doi:10.1371/journal.pgen.1002260.

Corella, D., Asensio, E. M., Coltell, O., Sorlí, J. V., Estruch, R., Martínez-González, M. Á., et al. (2016). CLOCK gene variation is associated with incidence of type-2 diabetes and cardiovascular diseases in type-2 diabetic subjects: dietary modulation in the PREDIMED randomized trial. *Cardiovasc. Diabetol.* 15, 4. doi:10.1186/s12933-015-0327-8.

Coronary Artery Disease Consortium, Samani, N. J., Deloukas, P., Erdmann, J., Hengstenberg, C., Kuulasmaa, K., et al. (2009). Large scale association analysis of novel genetic loci for coronary artery disease. *Arterioscler. Thromb. Vasc. Biol.* 29, 774–780. doi:10.1161/ATVBAHA.108.181388.

Costa-Urrutia, P., Abud, C., Franco-Trecu, V., Colistro, V., Rodríguez-Arellano, M. E., Vázquez-Pérez, J., et al. (2017). Genetic Obesity Risk and Attenuation Effect of Physical Fitness in Mexican-Mestizo Population: a Case-Control Study. *Ann. Hum. Genet.* 81, 106–116. doi:10.1111/ahg.12190.

Cox, A. J., Wing, M. R., Carr, J. J., Hightower, R. C., Smith, S. C., Xu, J., et al. (2011). Association of PNPLA3 SNP rs738409 with liver density in African Americans with type 2 diabetes mellitus. *Diabetes Metab.* 37, 452–455. doi:10.1016/j.diabet.2011.05.001.

Cruz, M., Valladares-Salgado, A., Garcia-Mena, J., Ross, K., Edwards, M., Angeles-Martinez, J., et al. (2010). Candidate gene association study conditioning on individual ancestry in patients with type 2 diabetes and metabolic syndrome from Mexico City. *Diabetes Metab. Res. Rev.* 26, 261–270. doi:10.1002/dmrr.1082.

Dashti, H. S., Follis, J. L., Smith, C. E., Tanaka, T., Garaulet, M., Gottlieb, D. J., et al. (2015). Gene-Environment Interactions of Circadian-Related Genes for Cardiometabolic Traits. *Diabetes Care* 38, 1456–1466. doi:10.2337/dc14-2709.

Dashti, H. S., Smith, C. E., Lee, Y.-C., Parnell, L. D., Lai, C.-Q., Arnett, D. K., et al. (2014). CRY1 circadian gene variant interacts with carbohydrate intake for insulin resistance in two independent populations: Mediterranean and North American. *Chronobiol. Int.* 31, 660–667. doi:10.3109/07420528.2014.886587.

Dastani, Z., Hivert, M.-F., Timpson, N., Perry, J. R. B., Yuan, X., Scott, R. A., et al. (2012). Novel loci for adiponectin levels and their influence on type 2 diabetes and metabolic traits: a multi-ethnic meta-analysis of 45,891 individuals. *PLoS Genet.* 8, e1002607. doi:10.1371/journal.pgen.1002607.

De Castro-Orós Isabel, Cenarro Ana, Tejedor María Teresa, Baila-Rueda Lucía, Mateo-Gallego Rocío, Lamiquiz-Moneo Itziar, et al. (2014). Common Genetic Variants Contribute to Primary Hypertriglyceridemia Without Differences Between Familial Combined Hyperlipidemia and Isolated Hypertriglyceridemia. *Circ. Cardiovasc. Genet.* 7, 814–821. doi:10.1161/CIRCGENETICS.114.000522.

de Luis, D. A., Izaola, O., Primo, D., and Aller, R. (2018). Association of the rs10830963 polymorphism in melatonin receptor type 1B (MTNR1B) with metabolic response after weight loss secondary to a hypocaloric diet based in Mediterranean style. *Clin. Nutr. Edinb. Scotl.* 37, 1563–1568. doi:10.1016/j.clnu.2017.08.015.

Dedoussis, G. V., Vidra, N., Butler, J., Papoutsakis, C., Yannakoulia, M., Hirschhorn, J. N., et al. (2009). Peroxisome proliferator-activated receptor-gamma (PPARgamma) Pro12Ala polymorphism and risk for pediatric obesity. *Clin. Chem. Lab. Med.* 47, 1047–1050. doi:10.1515/CCLM.2009.242.

Deeb, S. S., and Brunzell, J. D. (2009). The role of the PGC1α Gly482Ser polymorphism in weight gain due to intensive diabetes therapy. *PPAR Res.* 2009, 649286. doi:10.1155/2009/649286.

Dehghan Abbas, Dupuis Josée, Barbalic Maja, Bis Joshua C., Eiriksdottir Gudny, Lu Chen, et al. (2011). Meta-Analysis of Genome-Wide Association Studies in >80 000 Subjects Identifies Multiple Loci for C-Reactive Protein Levels. *Circulation* 123, 731–738. doi:10.1161/CIRCULATIONAHA.110.948570.

Deshmukh, H. A., Colhoun, H. M., Johnson, T., McKeigue, P. M., Betteridge, D. J., Durrington, P. N., et al. (2012). Genome-wide association study of genetic determinants of LDL-c response to atorvastatin therapy: importance of Lp(a). *J. Lipid Res.* 53, 1000–1011. doi:10.1194/jlr.P021113.

Devaney, J. M., Thompson, P. D., Visich, P. S., Saltarelli, W. A., Gordon, P. M., Orkunoglu-Suer, E. F., et al. (2011). The 1p13.3 LDL (C)-Associated Locus Shows Large Effect Sizes in Young Populations. *Pediatr. Res.* 69, 538–543. doi:10.1203/PDR.0b013e3182139227.

DIAbetes Genetics Replication And Meta-analysis (DIAGRAM) Consortium, Asian Genetic Epidemiology Network Type 2 Diabetes (AGEN-T2D) Consortium, South Asian Type 2 Diabetes (SAT2D) Consortium, Mexican American Type 2 Diabetes (MAT2D) Consortium, Type 2 Diabetes Genetic Exploration by Next-generation sequencing in multi-Ethnic Samples (T2D-GENES) Consortium, Mahajan, A., et al. (2014). Genome-wide trans-ancestry meta-analysis provides insight into the genetic architecture of type 2 diabetes susceptibility. *Nat. Genet.* 46, 234–244. doi:10.1038/ng.2897.

Ding, K., and Kullo, I. J. (2009). Genome-wide association studies for atherosclerotic vascular disease and its risk factors.

Circ. Cardiovasc. Genet. 2, 63–72. doi:10.1161/CIRCGENETICS.108.816751.

Ding, S., Liu, L., Zhuge, Q.-C., Yu, Z., Zhang, X., Xie, J., et al. (2012). The meta-analysis of the association of PPARG P12A, C161T polymorphism and coronary heart disease. *Wien. Klin. Wochenschr.* 124, 671–677. doi:10.1007/s00508-012-0223-0.

Dong, C., Zhou, H., Shen, C., Yu, L.-G., Ding, Y., Zhang, Y.-H., et al. (2015). Role of peroxisome proliferator-activated receptors gene polymorphisms in type 2 diabetes and metabolic syndrome. *World J. Diabetes* 6, 654–661. doi:10.4239/wjd.v6.i4.654.

Dongiovanni, P., Petta, S., Maglio, C., Fracanzani, A. L., Pipitone, R., Mozzi, E., et al. (2015). Transmembrane 6 superfamily member 2 gene variant disentangles nonalcoholic steatohepatitis from cardiovascular disease. *Hepatol. Baltim. Md* 61, 506–514. doi:10.1002/hep.27490.

Drenos, F., Talmud, P. J., Casas, J. P., Smeeth, L., Palmen, J., Humphries, S. E., et al. (2009). Integrated associations of genotypes with multiple blood biomarkers linked to coronary heart disease risk. *Hum. Mol. Genet.* 18, 2305–2316. doi:10.1093/hmg/ddp159.

Dumitrescu, L., Carty, C. L., Taylor, K., Schumacher, F. R., Hindorff, L. A., Ambite, J. L., et al. (2011). Genetic Determinants of Lipid Traits in Diverse Populations from the Population Architecture using Genomics and Epidemiology (PAGE) Study. *PLOS Genet.* 7, e1002138. doi:10.1371/journal.pgen.1002138.

Dupuis, J., Langenberg, C., Prokopenko, I., Saxena, R., Soranzo, N., Jackson, A. U., et al. (2010). New genetic loci implicated in fasting glucose homeostasis and their impact on type 2 diabetes risk. *Nat. Genet.* 42, 105–116. doi:10.1038/ng.520.

Edwards, D. R. V., Romero, R., Kusanovic, J. P., Hassan, S. S., Mazaki-Tovi, S., Vaisbuch, E., et al. (2011). Polymorphisms in maternal and fetal genes encoding for proteins involved in extracellular matrix metabolism alter the risk for small-for-gestational-age. *J. Matern. Fetal Neonatal Med.* 24, 362–380. doi:10.3109/14767058.2010.497572.

Ehrhardt, N., Doche, M. E., Chen, S., Mao, H. Z., Walsh, M. T., Bedoya, C., et al. (2017). Hepatic Tm6sf2 overexpression affects cellular ApoB-trafficking, plasma lipid levels, hepatic steatosis and atherosclerosis. *Hum. Mol. Genet.* 26, 2719–2731. doi:10.1093/hmg/ddx159.

Elbers, C. C., Guo, Y., Tragante, V., Iperen, E. P. A. van, Lanktree, M. B., Castillo, B. A., et al. (2012). Gene-Centric Meta-Analysis of Lipid Traits in African, East Asian and Hispanic Populations. *PLOS ONE* 7, e50198. doi:10.1371/journal.pone.0050198.

Elliott, P., Chambers, J. C., Zhang, W., Clarke, R., Hopewell, J. C., Peden, J. F., et al. (2009). Genetic Loci associated with C-reactive protein levels and risk of coronary heart disease. *JAMA* 302, 37–48. doi:10.1001/jama.2009.954.

Ellis, K. L., Frampton, C. M., Pilbrow, A. P., Troughton, R. W., Doughty, R. N., Whalley, G. A., et al. (2011). Genomic risk variants at 1p13.3, 1q41, and 3q22.3 are associated with subsequent cardiovascular outcomes in healthy controls and in established coronary artery disease. *Circ. Cardiovasc. Genet.* 4, 636–646. doi:10.1161/CIRCGENETICS.111.960336.

Emamian, M., Avan, A., Pasdar, A., Mirhafez, S. R., Sadeghzadeh, M., Moghadam, M. S., et al. (2015). The lipoprotein lipase S447X and cholesteryl ester transfer protein rs5882 polymorphisms and their relationship with lipid profile in human serum of obese individuals. *Gene* 558, 195–199. doi:10.1016/j.gene.2014.12.070.

Englund, A., Kovanen, L., Saarikoski, S. T., Haukka, J., Reunanen, A., Aromaa, A., et al. (2009). NPAS2 and PER2 are linked

to risk factors of the metabolic syndrome. *J. Circadian Rhythms* 7, 5. doi:10.1186/1740-3391-7-5.

Eslam, M., Mangia, A., Berg, T., Chan, H. L. Y., Irving, W. L., Dore, G. J., et al. (2016). Diverse impacts of the rs58542926 E167K variant in TM6SF2 on viral and metabolic liver disease phenotypes. *Hepatol. Baltim. Md* 64, 34–46. doi:10.1002/hep.28475.

Estivalet, A. A. F., Leiria, L. B., Dora, J. M., Rheinheimer, J., Bouças, A. P., Maia, A. L., et al. (2011). D2 Thr92Ala and PPARγ2 Pro12Ala polymorphisms interact in the modulation of insulin resistance in type 2 diabetic patients. *Obes. Silver Spring Md* 19, 825–832. doi:10.1038/oby.2010.231.

Evangelou, E., Warren, H. R., Mosen-Ansorena, D., Mifsud, B., Pazoki, R., Gao, H., et al. (2018). Genetic analysis of over 1 million people identifies 535 new loci associated with blood pressure traits. *Nat. Genet.* 50, 1412–1425. doi:10.1038/s41588-018-0205-x.

Falleti, E., Cussigh, A., Cmet, S., Fabris, C., and Toniutto, P. (2016). PNPLA3 rs738409 and TM6SF2 rs58542926 variants increase the risk of hepatocellular carcinoma in alcoholic cirrhosis. *Dig. Liver Dis. Off. J. Ital. Soc. Gastroenterol. Ital. Assoc. Study Liver* 48, 69–75. doi:10.1016/j.dld.2015.09.009.

Fan, J.-H., Xiang, M.-Q., Li, Q.-L., Shi, H.-T., and Guo, J.-J. (2016). PNPLA3 rs738409 Polymorphism Associated with Hepatic Steatosis and Advanced Fibrosis in Patients with Chronic Hepatitis C Virus: A Meta-Analysis. *Gut Liver* 10, 456–463. doi:10.5009/gnl15261.

Fan, W., Shen, C., Wu, M., Zhou, Z.-Y., and Guo, Z.-R. (2015). Association and Interaction of PPARα, δ, and γ Gene Polymorphisms with Low-Density Lipoprotein-Cholesterol in a Chinese Han Population. *Genet. Test. Mol. Biomark.* 19, 379–386. doi:10.1089/gtmb.2015.0002.

Fan, Y.-M., Raitakari, O. T., Kähönen, M., Hutri-Kähönen, N., Juonala, M., Marniemi, J., et al. (2009). Hepatic lipase promoter C-480T polymorphism is associated with serum lipids levels, but not subclinical atherosclerosis: the Cardiovascular Risk in Young Finns Study. *Clin. Genet.* 76, 46–53. doi:10.1111/j.1399-0004.2009.01180.x.

Feng, Q., Wei, W. Q., Chung, C. P., Levinson, R. T., Bastarache, L., Denny, J. C., et al. (2017a). The effect of genetic variation in PCSK9 on the LDL-cholesterol response to statin therapy. *Pharmacogenomics J.* 17, 204–208. doi:10.1038/tpj.2016.3.

Feng, Q., Wei, W.-Q., Levinson, R. T., Mosley, J. D., and Stein, C. M. (2017b). Replication and fine-mapping of genetic predictors of lipid traits in African-Americans. *J. Hum. Genet.* 62, 895–901. doi:10.1038/jhg.2017.55.

Flores, Y. N., Velázquez-Cruz, R., Ramírez, P., Bañuelos, M., Zhang, Z.-F., Yee, H. F., et al. (2016). Association between PNPLA3 (rs738409), LYPLAL1 (rs12137855), PPP1R3B (rs4240624), GCKR (rs780094), and elevated transaminase levels in overweight/obese Mexican adults. *Mol. Biol. Rep.* 43, 1359–1369. doi:10.1007/s11033-016-4058-z.

Franks, P. W., Christophi, C. A., Jablonski, K. A., Billings, L. K., Delahanty, L. M., Horton, E. S., et al. (2014). Common variation at PPARGC1A/B and change in body composition and metabolic traits following preventive interventions: the Diabetes Prevention Program. *Diabetologia* 57, 485–490. doi:10.1007/s00125-013-3133-4.

Fujimaki, T., Oguri, M., Horibe, H., Kato, K., Matsuoka, R., Abe, S., et al. (2015). Association of a transcription factor 21 gene polymorphism with hypertension. *Biomed. Rep.* 3, 118–122. doi:10.3892/br.2014.371.

Furusawa, T., Naka, I., Yamauchi, T., Natsuhara, K., Kimura, R., Nakazawa, M., et al. (2010). The Q223R polymorphism in LEPR is associated with obesity in Pacific Islanders. *Hum. Genet.* 127, 287–294. doi:10.1007/s00439-009-0768-9.

Futema, M., Kumari, M., Boustred, C., Kivimaki, M., and Humphries, S. E. (2015). Would raising the total cholesterol diagnostic cut-off from 7.5 mmol/L to 9.3 mmol/L improve detection rate of patients with monogenic familial hypercholesterolaemia? *Atherosclerosis* 239, 295–298. doi:10.1016/j.atherosclerosis.2015.01.028.

Galbete, C., Contreras, R., Martínez, J. A., Martínez-González, M. Á., Guillén-Grima, F., and Marti, A. (2012). Physical Activity and Sex Modulate Obesity Risk Linked to 3111T/C Gene Variant of the CLOCK Gene in an Elderly Population: The SUN Project. *Chronobiol. Int.* 29, 1397–1404. doi:10.3109/07420528.2012.728657.

Ganesan, M., Nizamuddin, S., Katkam, S. K., Kumaraswami, K., Hosad, U. K., Lobo, L. L., et al. (2016). c.*84G>A Mutation in CETP Is Associated with Coronary Artery Disease in South Indians. *PloS One* 11, e0164151. doi:10.1371/journal.pone.0164151.

Gao, X., Liu, W., Yang, L., Zhang, X., Ma, N., Wang, L., et al. (2017). Association between PNPLA3 gene polymorphisms and risk of hepatitis B virus-related hepatocellular carcinoma in Han population in China:a case-control study. *Scand. J. Gastroenterol.* 52, 1120–1127. doi:10.1080/00365521.2017.1334088.

Garaulet, M., Corbalán, M. D., Madrid, J. A., Morales, E., Baraza, J. C., Lee, Y. C., et al. (2010a). CLOCK gene is implicated in weight reduction in obese patients participating in a dietary programme based on the Mediterranean diet. *Int. J. Obes. 2005* 34, 516–523. doi:10.1038/ijo.2009.255.

Garaulet, M., Corbalán-Tutau, M. D., Madrid, J. A., Baraza, J. C., Parnell, L. D., Lee, Y.-C., et al. (2010b). PERIOD2 variants are associated with abdominal obesity, psycho-behavioral factors, and attrition in the dietary treatment of obesity. *J. Am. Diet. Assoc.* 110, 917–921. doi:10.1016/j.jada.2010.03.017.

Garaulet, M., Esteban Tardido, A., Lee, Y.-C., Smith, C. E., Parnell, L. D., and Ordovás, J. M. (2012). *SIRT1* and *CLOCK* 3111T>C combined genotype is associated with evening preference and weight loss resistance in a behavioral therapy treatment for obesity. *Int. J. Obes.* 36, 1436–1441. doi:10.1038/ijo.2011.270.

Garaulet, M., Gómez-Abellán, P., Rubio-Sastre, P., Madrid, J. A., Saxena, R., and Scheer, F. A. J. L. (2015). Common type 2 diabetes risk variant in MTNR1B worsens the deleterious effect of melatonin on glucose tolerance in humans. *Metabolism.* 64, 1650–1657. doi:10.1016/j.metabol.2015.08.003.

Garaulet, M., Lee, Y.-C., Shen, J., Parnell, L. D., Arnett, D. K., Tsai, M. Y., et al. (2009). CLOCK genetic variation and metabolic syndrome risk: modulation by monounsaturated fatty acids. *Am. J. Clin. Nutr.* 90, 1466–1475. doi:10.3945/ajcn.2009.27536.

Garaulet, M., Lee, Y.-C., Shen, J., Parnell, L. D., Arnett, D. K., Tsai, M. Y., et al. (2010). Genetic variants in human CLOCK associate with total energy intake and cytokine sleep factors in overweight subjects (GOLDN population). *Eur. J. Hum. Genet.* 18, 364–369. doi:10.1038/ejhg.2009.176.

Garaulet, M., Sánchez-Moreno, C., Smith, C. E., Lee, Y.-C., Nicolás, F., and Ordovás, J. M. (2011). Ghrelin, sleep reduction and evening preference: relationships to CLOCK 3111 T/C SNP and weight loss. *PloS One* 6, e17435.

doi:10.1371/journal.pone.0017435.

Garcia-Rios, A., Delgado-Lista, J., Perez-Martinez, P., Phillips, C. M., Ferguson, J. F., Gjelstad, I. M. F., et al. (2011). Genetic variations at the lipoprotein lipase gene influence plasma lipid concentrations and interact with plasma n-6 polyunsaturated fatty acids to modulate lipid metabolism. *Atherosclerosis* 218, 416–422. doi:10.1016/j.atherosclerosis.2011.07.092.

Garcia-Rios, A., Gomez-Delgado, F. J., Garaulet, M., Alcala-Diaz, J. F., Delgado-Lista, F. J., Marin, C., et al. (2014). Beneficial effect of CLOCK gene polymorphism rs1801260 in combination with low-fat diet on insulin metabolism in the patients with metabolic syndrome. *Chronobiol. Int.* 31, 401–408. doi:10.3109/07420528.2013.864300.

Garcia-Rios, A., Perez-Martinez, P., Delgado-Lista, J., Phillips, C. M., Gjelstad, I. M. F., Wright, J. W., et al. (2012). A Period 2 Genetic Variant Interacts with Plasma SFA to Modify Plasma Lipid Concentrations in Adults with Metabolic Syndrome. *J. Nutr.* 142, 1213–1218. doi:10.3945/jn.111.156968.

Gaulton, K. J., Willer, C. J., Li, Y., Scott, L. J., Conneely, K. N., Jackson, A. U., et al. (2008). Comprehensive association study of type 2 diabetes and related quantitative traits with 222 candidate genes. *Diabetes* 57, 3136–3144. doi:10.2337/db07-1731.

Gigante, B., Leander, K., Vikström, M., Ye, S., and de Faire, U. (2012). Chromosome 1p13 genetic variants antagonize the risk of myocardial infarction associated with high ApoB serum levels. *BMC Cardiovasc. Disord.* 12, 90. doi:10.1186/1471-2261-12-90.

Goffredo, M., Caprio, S., Feldstein, A. E., D'Adamo, E., Shaw, M. M., Pierpont, B., et al. (2016). Role of TM6SF2 rs58542926 in the pathogenesis of nonalcoholic pediatric fatty liver disease: A multiethnic study. *Hepatol. Baltim. Md* 63, 117–125. doi:10.1002/hep.28283.

Gomez-Delgado, F., Garcia-Rios, A., Alcala-Diaz, J. F., Rangel-Zuñiga, O., Delgado-Lista, J., Yubero-Serrano, E. M., et al. (2015). Chronic consumption of a low-fat diet improves cardiometabolic risk factors according to the CLOCK gene in patients with coronary heart disease. *Mol. Nutr. Food Res.* 59, 2556–2564. doi:10.1002/mnfr.201500375.

Goni, L., Cuervo, M., Milagro, F. I., and Martínez, J. A. (2014). Gene-Gene Interplay and Gene-Diet Interactions Involving the MTNR1B rs10830963 Variant with Body Weight Loss. *J. Nutr. Nutr.* 7, 232–242. doi:10.1159/000380951.

Goran, M. I., Walker, R., Le, K.-A., Mahurkar, S., Vikman, S., Davis, J. N., et al. (2010). Effects of PNPLA3 on liver fat and metabolic profile in Hispanic children and adolescents. *Diabetes* 59, 3127–3130. doi:10.2337/db10-0554.

Gouda, H. N., Sagoo, G. S., Harding, A.-H., Yates, J., Sandhu, M. S., and Higgins, J. P. T. (2010). The association between the peroxisome proliferator-activated receptor-gamma2 (PPARG2) Pro12Ala gene variant and type 2 diabetes mellitus: a HuGE review and meta-analysis. *Am. J. Epidemiol.* 171, 645–655. doi:10.1093/aje/kwp450.

Graff, M., North, K. E., Franceschini, N., Reiner, A. P., Feitosa, M., Carr, J. J., et al. (2013). PNPLA3 gene-by-visceral adipose tissue volume interaction and the pathogenesis of fatty liver disease: the NHLBI family heart study. *Int. J. Obes. 2005* 37, 432–438. doi:10.1038/ijo.2012.65.

Grallert, H., Dupuis, J., Bis, J. C., Dehghan, A., Barbalic, M., Baumert, J., et al. (2012). Eight genetic loci associated with variation in lipoprotein-associated phospholipase A2 mass and activity and coronary heart disease: meta-analysis of genome-wide association studies from five community-based studies. *Eur. Heart J.* 33, 238–251. doi:10.1093/eurheartj/ehr372.

Gregoor, J. G., van der Weide, J., Mulder, H., Cohen, D., van Megen, H. J. G. M., Egberts, A. C. G., et al. (2009). Polymorphisms of the LEP- and LEPR gene and obesity in patients using antipsychotic medication. *J. Clin. Psychopharmacol.* 29, 21–25. doi:10.1097/JCP.0b013e31819359be.

Gu, S.-J., Chen, D.-H., Guo, Z.-R., Zhou, Z.-Y., Hu, X.-S., and Wu, M. (2015). Effect of obesity on the association between common variations in the PPAR gene and C-reactive protein level in Chinese Han population. *Endocrine* 48, 195–202. doi:10.1007/s12020-014-0218-x.

Guardiola, M., Echeverria, P., González, M., Vallvé, J. C., Puig, J., Clotet, B., et al. (2015). Polymorphisms in LPL, CETP, and HL protect HIV-infected patients from atherogenic dyslipidemia in an allele-dose-dependent manner. *AIDS Res. Hum. Retroviruses* 31, 882–888. doi:10.1089/AID.2015.0061.

Guella, I., Asselta, R., Ardissino, D., Merlini, P. A., Peyvandi, F., Kathiresan, S., et al. (2010). Effects of PCSK9 genetic variants on plasma LDL cholesterol levels and risk of premature myocardial infarction in the Italian population. *J. Lipid Res.* 51, 3342–3349. doi:10.1194/jlr.M010009.

Guichelaar, M. M. J., Gawrieh, S., Olivier, M., Viker, K., Krishnan, A., Sanderson, S., et al. (2013). Interactions of allelic variance of PNPLA3 with nongenetic factors in predicting nonalcoholic steatohepatitis and nonhepatic complications of severe obesity. *Obes. Silver Spring Md* 21, 1935–1941. doi:10.1002/oby.20327.

Guo, J., Luo, Y. X., Tao, L. X., and Guo, X. H. (2015a). Association between 1p13.3 genomic markers and coronary artery disease: a meta-analysis involving patients and controls. *Genet. Mol. Res. GMR* 14, 9092–9102. doi:10.4238/2015.August.7.18.

Guo, S., Hu, Y., Ding, Y., Liu, J., Zhang, M., Ma, R., et al. (2015b). Association between Eight Functional Polymorphisms and Haplotypes in the Cholesterol Ester Transfer Protein (CETP) Gene and Dyslipidemia in National Minority Adults in the Far West Region of China. *Int. J. Environ. Res. Public. Health* 12, 15979–15992. doi:10.3390/ijerph121215036.

Guo, T., Yin, R.-X., Lin, W.-X., Wang, W., Huang, F., and Pan, S.-L. (2016). Association of the variants and haplotypes in the DOCK7, PCSK9 and GALNT2 genes and the risk of hyperlipidaemia. *J. Cell. Mol. Med.* 20, 243–265. doi:10.1111/jcmm.12713.

Gupta, R., Ejebe, K., Butler, J., Lettre, G., Lyon, H., Guiducci, C., et al. (2010). Association of common DNA sequence variants at 33 genetic loci with blood lipids in individuals of African ancestry from Jamaica. *Hum. Genet.* 128, 557–561. doi:10.1007/s00439-010-0887-3.

Guyot, E., Sutton, A., Rufat, P., Laguillier, C., Mansouri, A., Moreau, R., et al. (2013). PNPLA3 rs738409, hepatocellular carcinoma occurrence and risk model prediction in patients with cirrhosis. *J. Hepatol.* 58, 312–318. doi:10.1016/j.jhep.2012.09.036.

Haas Blake E., Weissglas-Volkov Daphna, Aguilar-Salinas Carlos A., Nikkola Elina, Vergnes Laurent, Cruz-Bautista Ivette, et al. (2011). Evidence of How rs7575840 Influences Apolipoprotein B–Containing Lipid Particles. *Arterioscler. Thromb. Vasc. Biol.* 31, 1201–1207. doi:10.1161/ATVBAHA.111.224139.

Hamrefors, V., Orho-Melander, M., Krauss, R. M., Hedblad, B., Almgren, P., Berglund, G., et al. (2010). A gene score of nine LDL and HDL regulating genes is associated with fluvastatin-induced cholesterol changes in women. *J. Lipid Res.* 51, 625–634. doi:10.1194/jlr.P001792.

Hanh, N. T. H., Nhung, B. T., Dao, D. T. A., Tuyet, L. T., Hop, L. T., Binh, T. Q., et al. (2016). Association of apolipoprotein E polymorphism with plasma lipid disorders, independent of obesity-related traits in Vietnamese children. *Lipids Health Dis.* 15, 176. doi:10.1186/s12944-016-0349-6.

Hassan, M. M., Kaseb, A., Etzel, C. J., El-Serag, H., Spitz, M. R., Chang, P., et al. (2013). Genetic variation in the PNPLA3 gene and hepatocellular carcinoma in USA: risk and prognosis prediction. *Mol. Carcinog.* 52 Suppl 1, E139-147. doi:10.1002/mc.22057.

He, Q.-C., Hu, Y.-Y., Zhang, Q.-P., Tan, L.-L., Liu, Y.-H., Liu, T., et al. (2017). A meta-analysis of three identified single nucleotide polymorphisms at 1p13.3 and 1q41 and their associations with lipid levels and coronary artery disease. *Kaohsiung J. Med. Sci.* 33, 1–10. doi:10.1016/j.kjms.2016.10.011.

Hebbar, P., Elkum, N., Alkayal, F., John, S. E., Thanaraj, T. A., and Alsmadi, O. (2017). Genetic risk variants for metabolic traits in Arab populations. *Sci. Rep.* 7, 40988. doi:10.1038/srep40988.

Hegele, R. A., Ban, M. R., Hsueh, N., Kennedy, B. A., Cao, H., Zou, G. Y., et al. (2009). A polygenic basis for four classical Fredrickson hyperlipoproteinemia phenotypes that are characterized by hypertriglyceridemia. *Hum. Mol. Genet.* 18, 4189–4194. doi:10.1093/hmg/ddp361.

Hernaez, R., McLean, J., Lazo, M., Brancati, F. L., Hirschhorn, J. N., Borecki, I. B., et al. (2013). Association between variants in or near PNPLA3, GCKR, and PPP1R3B with ultrasound-defined steatosis based on data from the third National Health and Nutrition Examination Survey. *Clin. Gastroenterol. Hepatol. Off. Clin. Pract. J. Am. Gastroenterol. Assoc.* 11, 1183-1190.e2. doi:10.1016/j.cgh.2013.02.011.

Hindy, G., Mollet, I. G., Rukh, G., Ericson, U., and Orho-Melander, M. (2016). Several type 2 diabetes-associated variants in genes annotated to WNT signaling interact with dietary fiber in relation to incidence of type 2 diabetes. *Genes Nutr.* 11, 6. doi:10.1186/s12263-016-0524-4.

Hodoglugil, U., Williamson, D. W., and Mahley, R. W. (2010). Polymorphisms in the hepatic lipase gene affect plasma HDL-cholesterol levels in a Turkish population. *J. Lipid Res.* 51, 422–430. doi:10.1194/jlr.P001578.

Hodžić, A., Lavtar, P., Ristanović, M., Novaković, I., Dotlić, J., and Peterlin, B. (2018). Genetic variation in the CLOCK gene is associated with idiopathic recurrent spontaneous abortion. *PloS One* 13, e0196345. doi:10.1371/journal.pone.0196345.

Hodžić, A., Ristanović, M., Zorn, B., Tulić, C., Maver, A., Novaković, I., et al. (2013). Genetic Variation in Circadian Rhythm Genes CLOCK and ARNTL as Risk Factor for Male Infertility. *PLOS ONE* 8, e59220. doi:10.1371/journal.pone.0059220.

Hoffmann, T. J., Choquet, H., Yin, J., Banda, Y., Kvale, M. N., Glymour, M., et al. (2018). A Large Multiethnic Genome-Wide Association Study of Adult Body Mass Index Identifies Novel Loci. *Genetics* 210, 499–515. doi:10.1534/genetics.118.301479.

Holzapfel, C., Siegrist, M., Rank, M., Langhof, H., Grallert, H., Baumert, J., et al. (2011). Association of a MTNR1B gene variant with fasting glucose and HOMA-B in children and adolescents with high BMI-SDS. *Eur. J. Endocrinol.* 164, 205–212. doi:10.1530/EJE-10-0588.

Horikoshi, M., Beaumont, R. N., Day, F. R., Warrington, N. M., Kooijman, M. N., Fernandez-Tajes, J., et al. (2016). Genome-

wide associations for birth weight and correlations with adult disease. *Nature* 538, 248–252. doi:10.1038/nature19806.

Hotta, K., Yoneda, M., Hyogo, H., Ochi, H., Mizusawa, S., Ueno, T., et al. (2010). Association of the rs738409 polymorphism in PNPLA3 with liver damage and the development of nonalcoholic fatty liver disease. *BMC Med. Genet.* 11, 172. doi:10.1186/1471-2350-11-172.

Hou, H., Ma, R., Guo, H., He, J., Hu, Y., Mu, L., et al. (2017). Association between Six CETP Polymorphisms and Metabolic Syndrome in Uyghur Adults from Xinjiang, China. *Int. J. Environ. Res. Public. Health* 14. doi:10.3390/ijerph14060653.

Hsiao, T.-J., and Lin, E. (2015). The Pro12Ala polymorphism in the peroxisome proliferator-activated receptor gamma (PPARG) gene in relation to obesity and metabolic phenotypes in a Taiwanese population. *Endocrine* 48, 786–793. doi:10.1007/s12020-014-0407-7.

Hu, C., Zhang, R., Wang, C., Wang, J., Ma, X., Hou, X., et al. (2010a). Variants from GIPR, TCF7L2, DGKB, MADD, CRY2, GLIS3, PROX1, SLC30A8 and IGF1 Are Associated with Glucose Metabolism in the Chinese. *PLOS ONE* 5, e15542. doi:10.1371/journal.pone.0015542.

Hu, C., Zhang, R., Wang, C., Wang, J., Ma, X., Hou, X., et al. (2010b). Variants from GIPR, TCF7L2, DGKB, MADD, CRY2, GLIS3, PROX1, SLC30A8 and IGF1 Are Associated with Glucose Metabolism in the Chinese. *PLOS ONE* 5, e15542. doi:10.1371/journal.pone.0015542.

Hu, C., Zhang, R., Wang, C., Yu, W., Lu, J., Ma, X., et al. (2010c). Effects of GCK, GCKR, G6PC2 and MTNR1B variants on glucose metabolism and insulin secretion. *PloS One* 5, e11761. doi:10.1371/journal.pone.0011761.

Hu, Y. H., Liu, J. M., Zhang, M., He, J., Yan, Y. Z., Ma, J. L., et al. (2016). [Association between CETP polymorphisms and haplotypes with dyslipidemia in Xinjiang Uygur and Kazak residents]. *Zhonghua Xin Xue Guan Bing Za Zhi* 44, 671–677. doi:10.3760/cma.j.issn.0253-3758.2016.08.007.

Huang, C.-F., Chen, J.-J., Yeh, M.-L., Huang, C.-I., Hsieh, M.-Y., Yang, H.-L., et al. (2015a). PNPLA3 genetic variants determine hepatic steatosis in non-obese chronic hepatitis C patients. *Sci. Rep.* 5, 11901. doi:10.1038/srep11901.

Huang, C.-F., Dai, C.-Y., Yeh, M.-L., Huang, C.-I., Tai, C.-M., Hsieh, M.-H., et al. (2015b). Association of diabetes and PNPLA3 genetic variants with disease severity of patients with chronic hepatitis C virus infection. *J. Hepatol.* 62, 512–518. doi:10.1016/j.jhep.2014.10.011.

Huang, C.-M., Chang, K.-C., Hung, C.-H., Chiu, K.-W., Lu, S.-N., Wang, J.-H., et al. (2017). Impact of PNPLA3 and IFNL3 polymorphisms on hepatic steatosis in Asian patients with chronic hepatitis C. *PloS One* 12, e0182204. doi:10.1371/journal.pone.0182204.

Huang, W.-H., Hwang, L.-C., Chan, H.-L., Lin, H.-Y., and Lin, Y.-H. (2016). Study of seven single-nucleotide polymorphisms identified in East Asians for association with obesity in a Taiwanese population. *BMJ Open* 6, e011713. doi:10.1136/bmjopen-2016-011713.

Hubacek, J. A., Adamkova, V., Lanska, V., and Dlouha, D. (2017). Polygenic hypercholesterolemia: examples of GWAS results and their replication in the Czech-Slavonic population. *Physiol. Res.* 66, S101–S111.

Huopio, H., Cederberg, H., Vangipurapu, J., Hakkarainen, H., Pääkkönen, M., Kuulasmaa, T., et al. (2013). Association of risk variants for type 2 diabetes and hyperglycemia with gestational diabetes. *Eur. J. Endocrinol.* 169, 291–297. doi:10.1530/EJE-13-0286.

Ingelsson, E., Langenberg, C., Hivert, M.-F., Prokopenko, I., Lyssenko, V., Dupuis, J., et al. (2010). Detailed Physiologic Characterization Reveals Diverse Mechanisms for Novel Genetic Loci Regulating Glucose and Insulin Metabolism in Humans. *Diabetes* 59, 1266–1275. doi:10.2337/db09-1568.

Inouye, M., Ripatti, S., Kettunen, J., Lyytikäinen, L.-P., Oksala, N., Laurila, P.-P., et al. (2012). Novel Loci for Metabolic Networks and Multi-Tissue Expression Studies Reveal Genes for Atherosclerosis. *PLOS Genet.* 8, e1002907. doi:10.1371/journal.pgen.1002907.

Jansen, M. D., Knudsen, G. P., Myhre, R., Høiseth, G., Mørland, J., Næss, Ø., et al. (2014). Genetic variants in loci 1p13 and 9p21 and fatal coronary heart disease in a Norwegian case-cohort study. *Mol. Biol. Rep.* 41, 2733–2743. doi:10.1007/s11033-014-3096-7.

Jeemon, P., Pettigrew, K., Sainsbury, C., Prabhakaran, D., and Padmanabhan, S. (2011). Implications of discoveries from genome-wide association studies in current cardiovascular practice. *World J. Cardiol.* 3, 230–247. doi:10.4330/wjc.v3.i7.230.

Jia, J., Ding, H., Yang, K., Mao, L., Zhao, H., Zhan, Y., et al. (2015). Vitamin D Receptor Genetic Polymorphism Is Significantly Associated with Risk of Type 2 Diabetes Mellitus in Chinese Han Population. *Arch. Med. Res.* 46, 572–579. doi:10.1016/j.arcmed.2015.09.006.

Jiménez-Sousa, M. A., Berenguer, J., García-Álvarez, M., Gutierrez-Rivas, M., Aldámiz-Echevarria, T., Tejerina, F., et al. (2016). Impact of patatin-like phospholipase domain-containing 3 gene polymorphism (rs738409) on severity of liver disease in HIV/hepatitis C virus-coinfected patients. *AIDS Lond. Engl.* 30, 465–470. doi:10.1097/QAD.0000000000000908.

Johansson, L. E., Lindblad, U., Larsson, C. A., Råstam, L., and Ridderstråle, M. (2008). Polymorphisms in the adiponutrin gene are associated with increased insulin secretion and obesity. *Eur. J. Endocrinol.* 159, 577–583. doi:10.1530/EJE-08-0426.

Kanai, M., Akiyama, M., Takahashi, A., Matoba, N., Momozawa, Y., Ikeda, M., et al. (2018). Genetic analysis of quantitative traits in the Japanese population links cell types to complex human diseases. *Nat. Genet.* 50, 390. doi:10.1038/s41588-018-0047-6.

Kantartzis, K., Peter, A., Machicao, F., Machann, J., Wagner, S., Königsrainer, I., et al. (2009). Dissociation between fatty liver and insulin resistance in humans carrying a variant of the patatin-like phospholipase 3 gene. *Diabetes* 58, 2616–2623. doi:10.2337/db09-0279.

Kanth, V. V. R., Sasikala, M., Sharma, M., Rao, P. N., and Reddy, D. N. (2016). Genetics of non-alcoholic fatty liver disease: From susceptibility and nutrient interactions to management. *World J. Hepatol.* 8, 827–837. doi:10.4254/wjh.v8.i20.827.

Kasim, N. B., Huri, H. Z., Vethakkan, S. R., Ibrahim, L., and Abdullah, B. M. (2016). Genetic polymorphisms associated with overweight and obesity in uncontrolled Type 2 diabetes mellitus. *Biomark. Med.* 10, 403–415. doi:10.2217/bmm-2015-0037.

Kathiresan, S., Manning, A. K., Demissie, S., D'Agostino, R. B., Surti, A., Guiducci, C., et al. (2007). A genome-wide association study for blood lipid phenotypes in the Framingham Heart Study. *BMC Med. Genet.* 8, S17. doi:10.1186/1471-2350-8-S1-S17.

Kathiresan, S., Melander, O., Guiducci, C., Surti, A., Burtt, N. P., Rieder, M. J., et al. (2008). Six new loci associated with blood low-density lipoprotein cholesterol, high-density lipoprotein cholesterol or triglycerides in humans. *Nat. Genet.* 40, 189–197. doi:10.1038/ng.75.

Kathiresan, S., Willer, C. J., Peloso, G. M., Demissie, S., Musunuru, K., Schadt, E. E., et al. (2009). Common variants at 30 loci contribute to polygenic dyslipidemia. *Nat. Genet.* 41, 56–65. doi:10.1038/ng.291.

Kawaguchi, T., Sumida, Y., Umemura, A., Matsuo, K., Takahashi, M., Takamura, T., et al. (2012). Genetic polymorphisms of the human PNPLA3 gene are strongly associated with severity of non-alcoholic fatty liver disease in Japanese. *PloS One* 7, e38322. doi:10.1371/journal.pone.0038322.

Keaton, J. M., Gao, C., Guan, M., Hellwege, J. N., Palmer, N. D., Pankow, J. S., et al. (2018). Genome-wide interaction with the insulin secretion locus MTNR1B reveals CMIP as a novel type 2 diabetes susceptibility gene in African Americans. *Genet. Epidemiol.* 42, 559–570. doi:10.1002/gepi.22126.

Kedenko, L., Lamina, C., Kedenko, I., Kollerits, B., Kiesslich, T., Iglseder, B., et al. (2014). Genetic polymorphisms at SIRT1 and FOXO1 are associated with carotid atherosclerosis in the SAPHIR cohort. *BMC Med. Genet.* 15, 112. doi:10.1186/s12881-014-0112-7.

Keebler, M. E., Deo, R. C., Surti, A., Konieczkowski, D., Guiducci, C., Burtt, N., et al. (2010). Fine-Mapping in African Americans of 8 Recently Discovered Genetic Loci for Plasma Lipids. *Circ. Genomic Precis. Med.* Available at: https://www.ahajournals.org/doi/full/10.1161/CIRCGENETICS.109.914267 [Accessed October 5, 2018].

Keebler Mary E., Sanders Christopher L., Surti Aarti, Guiducci Candace, Burtt Noel P., and Kathiresan Sekar (2009). Association of Blood Lipids With Common DNA Sequence Variants at 19 Genetic Loci in the Multiethnic United States National Health and Nutrition Examination Survey III. *Circ. Cardiovasc. Genet.* 2, 238–243. doi:10.1161/CIRCGENETICS.108.829473.

Keller, M., Schleinitz, D., Förster, J., Tönjes, A., Böttcher, Y., Fischer-Rosinsky, A., et al. (2013). THOC5: a novel gene involved in HDL-cholesterol metabolism. *J. Lipid Res.* 54, 3170–3176. doi:10.1194/jlr.M039420.

Kelly, M. A., Rees, S. D., Hydrie, M. Z. I., Shera, A. S., Bellary, S., O'Hare, J. P., et al. (2012). Circadian Gene Variants and Susceptibility to Type 2 Diabetes: A Pilot Study. *PLOS ONE* 7, e32670. doi:10.1371/journal.pone.0032670.

Kenny, E. E., Kim, M., Gusev, A., Lowe, J. K., Salit, J., Smith, J. G., et al. (2011). Increased power of mixed models facilitates association mapping of 10 loci for metabolic traits in an isolated population. *Hum. Mol. Genet.* 20, 827–839. doi:10.1093/hmg/ddq510.

Kettunen, J., Demirkan, A., Würtz, P., Draisma, H. H. M., Haller, T., Rawal, R., et al. (2016). Genome-wide study for circulating metabolites identifies 62 loci and reveals novel systemic effects of *LPA*. *Nat. Commun.* 7, 11122. doi:10.1038/ncomms11122.

Kettunen, J., Tukiainen, T., Sarin, A.-P., Ortega-Alonso, A., Tikkanen, E., Lyytikäinen, L.-P., et al. (2012). Genome-wide association study identifies multiple loci influencing human serum metabolite levels. *Nat. Genet.* 44, 269–276. doi:10.1038/ng.1073.

Khetarpal, S. A., Edmondson, A. C., Raghavan, A., Neeli, H., Jin, W., Badellino, K. O., et al. (2011). Mining the LIPG Allelic

Spectrum Reveals the Contribution of Rare and Common Regulatory Variants to HDL Cholesterol. *PLOS Genet.* 7, e1002393. doi:10.1371/journal.pgen.1002393.

Kilic, U., Gok, O., Bacaksiz, A., Izmirli, M., Elibol-Can, B., and Uysal, O. (2014). SIRT1 gene polymorphisms affect the protein expression in cardiovascular diseases. *PloS One* 9, e90428. doi:10.1371/journal.pone.0090428.

Kilpeläinen, T. O., Lakka, T. A., Laaksonen, D. E., Lindström, J., Eriksson, J. G., Valle, T. T., et al. (2008). SNPs in PPARG associate with type 2 diabetes and interact with physical activity. *Med. Sci. Sports Exerc.* 40, 25–33. doi:10.1249/mss.0b013e318159d1cd.

Kim, D. S., Jackson, A. U., Li, Y. K., Stringham, H. M., FinMetSeq Investigators, Kuusisto, J., et al. (2017a). Novel association of TM6SF2 rs58542926 genotype with increased serum tyrosine levels and decreased apoB-100 particles in Finns. *J. Lipid Res.* 58, 1471–1481. doi:10.1194/jlr.P076034.

Kim, H., Lee, K.-W., Lee, K., Seo, S., Park, M.-Y., Ahn, S. W., et al. (2018). Effect of PNPLA3 I148M polymorphism on histologically proven non-alcoholic fatty liver disease in liver transplant recipients. *Hepatol. Res. Off. J. Jpn. Soc. Hepatol.* 48, E162–E171. doi:10.1111/hepr.12940.

Kim, T., Park, A. Y., Baek, Y., and Cha, S. (2017b). Genome-Wide Association Study Reveals Four Loci for Lipid Ratios in the Korean Population and the Constitutional Subgroup. *PLOS ONE* 12, e0168137. doi:10.1371/journal.pone.0168137.

Kim, Y. J., Go, M. J., Hu, C., Hong, C. B., Kim, Y. K., Lee, J. Y., et al. (2011). Large-scale genome-wide association studies in East Asians identify new genetic loci influencing metabolic traits. *Nat. Genet.* 43, 990–995. doi:10.1038/ng.939.

Kitamoto, T., Kitamoto, A., Ogawa, Y., Honda, Y., Imajo, K., Saito, S., et al. (2015). Targeted-bisulfite sequence analysis of the methylation of CpG islands in genes encoding PNPLA3, SAMM50, and PARVB of patients with non-alcoholic fatty liver disease. *J. Hepatol.* 63, 494–502. doi:10.1016/j.jhep.2015.02.049.

Klarin, D., Damrauer, S. M., Cho, K., Sun, Y. V., Teslovich, T. M., Honerlaw, J., et al. (2018). Genetics of blood lipids among ~300,000 multi-ethnic participants of the Million Veteran Program. *Nat. Genet.* 50, 1514. doi:10.1038/s41588-018-0222-9.

Kleber, M. E., Renner, W., Grammer, T. B., Linsel-Nitschke, P., Boehm, B. O., Winkelmann, B. R., et al. (2010). Association of the single nucleotide polymorphism rs599839 in the vicinity of the sortilin 1 gene with LDL and triglyceride metabolism, coronary heart disease and myocardial infarction. The Ludwigshafen Risk and Cardiovascular Health Study. *Atherosclerosis* 209, 492–497. doi:10.1016/j.atherosclerosis.2009.09.068.

Komurcu-Bayrak, E., Onat, A., Yuzbasiogullari, B., Mononen, N., Laaksonen, R., Kähönen, M., et al. (2011). The APOE -219G/T and +113G/C polymorphisms affect insulin resistance among Turks. *Metabolism.* 60, 655–663. doi:10.1016/j.metabol.2010.06.016.

Kong, X., Zhang, X., Xing, X., Zhang, B., Hong, J., and Yang, W. (2015). The Association of Type 2 Diabetes Loci Identified in Genome-Wide Association Studies with Metabolic Syndrome and Its Components in a Chinese Population with Type 2 Diabetes. *PloS One* 10, e0143607. doi:10.1371/journal.pone.0143607.

Koroglu, O. A., Onay, H., Cakmak, B., Bilgin, B., Yalaz, M., Tunc, S., et al. (2014). Association of vitamin D receptor gene

polymorphisms and bronchopulmonary dysplasia. *Pediatr. Res.* 76, 171. doi:10.1038/pr.2014.63.

Kotronen, A., Johansson, L. E., Johansson, L. M., Roos, C., Westerbacka, J., Hamsten, A., et al. (2009). A common variant in PNPLA3, which encodes adiponutrin, is associated with liver fat content in humans. *Diabetologia* 52, 1056–1060. doi:10.1007/s00125-009-1285-z.

Kovac, U., and Rozman, D. (2015). "Genetics of Non-alcoholic Fatty Liver Disease," in, 1–8. doi:10.1002/9780470015902.a0025335.

Kovanen, L., Saarikoski, S. T., Aromaa, A., Lönnqvist, J., and Partonen, T. (2010). ARNTL (BMAL1) and NPAS2 Gene Variants Contribute to Fertility and Seasonality. *PLOS ONE* 5, e10007. doi:10.1371/journal.pone.0010007.

Krawczyk, M., Grünhage, F., Zimmer, V., and Lammert, F. (2011). Variant adiponutrin (PNPLA3) represents a common fibrosis risk gene: non-invasive elastography-based study in chronic liver disease. *J. Hepatol.* 55, 299–306. doi:10.1016/j.jhep.2010.10.042.

Krawczyk, M., Rau, M., Schattenberg, J. M., Bantel, H., Pathil, A., Demir, M., et al. (2017). Combined effects of the PNPLA3 rs738409, TM6SF2 rs58542926, and MBOAT7 rs641738 variants on NAFLD severity: a multicenter biopsy-based study. *J. Lipid Res.* 58, 247–255. doi:10.1194/jlr.P067454.

Krishnan, M., Shelling, A. N., Wall, C. R., Mitchell, E. A., Murphy, R., McCowan, L. M. E., et al. (2017). Gene-by-environment interactions of the CLOCK, PEMT, and GHRELIN loci with average sleep duration in relation to obesity traits using a cohort of 643 New Zealand European children. *Sleep Med.* 37, 19–26. doi:10.1016/j.sleep.2017.05.017.

Kupcinskas, J., Valantiene, I., Varkalaitė, G., Steponaitiene, R., Skieceviciene, J., Sumskiene, J., et al. (2017). PNPLA3 and RNF7 Gene Variants are Associated with the Risk of Developing Liver Fibrosis and Cirrhosis in an Eastern European Population. *J. Gastrointest. Liver Dis. JGLD* 26, 37–43. doi:10.15403/jgld.2014.1121.261.pnp.

Kurano, M., Tsukamoto, K., Kamitsuji, S., Kamatani, N., Hara, M., Ishikawa, T., et al. (2016). Genome-wide association study of serum lipids confirms previously reported associations as well as new associations of common SNPs within PCSK7 gene with triglyceride. *J. Hum. Genet.* 61, 427–433. doi:10.1038/jhg.2015.170.

Kwak, S. H., Kim, S.-H., Cho, Y. M., Go, M. J., Cho, Y. S., Choi, S. H., et al. (2012). A Genome-Wide Association Study of Gestational Diabetes Mellitus in Korean Women. *Diabetes* 61, 531–541. doi:10.2337/db11-1034.

Lane, J. M., Chang, A.-M., Bjonnes, A. C., Aeschbach, D., Anderson, C., Cade, B. E., et al. (2016). Impact of Common Diabetes Risk Variant in MTNR1B on Sleep, Circadian, and Melatonin Physiology. *Diabetes* 65, 1741–1751. doi:10.2337/db15-0999.

Langenberg, C., Pascoe, L., Mari, A., Tura, A., Laakso, M., Frayling, T. M., et al. (2009). Common genetic variation in the melatonin receptor 1B gene (MTNR1B) is associated with decreased early-phase insulin response. *Diabetologia* 52, 1537. doi:10.1007/s00125-009-1392-x.

Langlois, C., Abadi, A., Peralta-Romero, J., Alyass, A., Suarez, F., Gomez-Zamudio, J., et al. (2016). Evaluating the transferability of 15 European-derived fasting plasma glucose SNPs in Mexican children and adolescents. *Sci. Rep.* 6, 36202. doi:10.1038/srep36202.

Lanktree, M. B., Anand, S. S., Yusuf, S., Hegele, R. A., and Investigators, the S. (2009). Replication of genetic associations with plasma lipoprotein traits in a multiethnic sample. *J. Lipid Res.* 50, 1487–1496. doi:10.1194/jlr.P900008-JLR200.

Larifla, L., Beaney, K. E., Foucan, L., Bangou, J., Michel, C. T., Martino, J., et al. (2016). Influence of Genetic Risk Factors on Coronary Heart Disease Occurrence in Afro-Caribbeans. *Can. J. Cardiol.* 32, 978–985. doi:10.1016/j.cjca.2016.01.004.

Lee, S. S., Byoun, Y.-S., Jeong, S.-H., Woo, B. H., Jang, E. S., Kim, J.-W., et al. (2014). Role of the PNPLA3 I148M polymorphism in nonalcoholic fatty liver disease and fibrosis in Korea. *Dig. Dis. Sci.* 59, 2967–2974. doi:10.1007/s10620-014-3279-z.

Legry, V., Bokor, S., Beghin, L., Galfo, M., Gonzalez-Gross, M., Molnar, D., et al. (2011). Associations between common genetic polymorphisms in the liver X receptor alpha and its target genes with the serum HDL-cholesterol concentration in adolescents of the HELENA Study. *Atherosclerosis* 216, 166–169. doi:10.1016/j.atherosclerosis.2011.01.031.

Lettre, G., Palmer, C. D., Young, T., Ejebe, K. G., Allayee, H., Benjamin, E. J., et al. (2011). Genome-Wide Association Study of Coronary Heart Disease and Its Risk Factors in 8,090 African Americans: The NHLBI CARe Project. *PLOS Genet.* 7, e1001300. doi:10.1371/journal.pgen.1001300.

Li, L., Hua, J., Jian-Ping, H., and Yan, L. (2015). Association between the Lipid Levels and Single Nucleotide Polymorphisms of ABCA1, APOE and HMGCR Genes in Subjects with Spontaneous Preterm Delivery. *PLOS ONE* 10, e0135785. doi:10.1371/journal.pone.0135785.

Li, S., Xu, H., Li, S.-C., Qi, X.-Q., and Sun, W.-J. (2014). Vitamin D receptor rs2228570 polymorphism and susceptibly to ovarian cancer: a meta-analysis. *Tumour Biol. J. Int. Soc. Oncodevelopmental Biol. Med.* 35, 1319–1322. doi:10.1007/s13277-013-1175-3.

Li, Y., Huang, Y., Liang, X., Long, B., Chen, S., Lian, J., et al. (2017). Apolipoprotein C-I Polymorphism and Its Association with Serum Lipid Levels and Longevity in the Bama Population. *Int. J. Environ. Res. Public. Health* 14. doi:10.3390/ijerph14050505.

Liao, S., Liu, Y., Tan, Y., Gan, L., Mei, J., Song, W., et al. (2012). Association of Genetic Variants of Melatonin Receptor 1B with Gestational Plasma Glucose Level and Risk of Glucose Intolerance in Pregnant Chinese Women. *PLOS ONE* 7, e40113. doi:10.1371/journal.pone.0040113.

Ligthart, S., Vaez, A., Hsu, Y.-H., Stolk, R., Uitterlinden, A. G., Hofman, A., et al. (2016). Bivariate genome-wide association study identifies novel pleiotropic loci for lipids and inflammation. *BMC Genomics* 17, 443. doi:10.1186/s12864-016-2712-4.

Lin, Y.-C., Chang, P.-F., Chang, M.-H., and Ni, Y.-H. (2013). A common variant in the peroxisome proliferator-activated receptor-γ coactivator-1α gene is associated with nonalcoholic fatty liver disease in obese children. *Am. J. Clin. Nutr.* 97, 326–331. doi:10.3945/ajcn.112.046417.

Lin, Y.-C., Chang, P.-F., Chang, M.-H., and Ni, Y.-H. (2014). Genetic variants in GCKR and PNPLA3 confer susceptibility to nonalcoholic fatty liver disease in obese individuals. *Am. J. Clin. Nutr.* 99, 869–874. doi:10.3945/ajcn.113.079749.

Lin, Y.-C., Chang, P.-F., Hu, F.-C., Yang, W.-S., Chang, M.-H., and Ni, Y.-H. (2011). A common variant in the PNPLA3 gene is a risk factor for non-alcoholic fatty liver disease in obese Taiwanese children. *J. Pediatr.* 158, 740–744.

doi:10.1016/j.jpeds.2010.11.016.

Linsel-Nitschke, P., Heeren, J., Aherrahrou, Z., Bruse, P., Gieger, C., Illig, T., et al. (2010). Genetic variation at chromosome 1p13.3 affects sortilin mRNA expression, cellular LDL-uptake and serum LDL levels which translates to the risk of coronary artery disease. *Atherosclerosis* 208, 183–189. doi:10.1016/j.atherosclerosis.2009.06.034.

Liu, C., Wu, Y., Li, H., Qi, Q., Langenberg, C., Loos, R. J. F., et al. (2010). MTNR1B rs10830963 is associated with fasting plasma glucose, HbA1C and impaired beta-cell function in Chinese Hans from Shanghai. *BMC Med. Genet.* 11, 59. doi:10.1186/1471-2350-11-59.

Liu, Y., Zhou, D., Zhang, Z., Song, Y., Zhang, D., Zhao, T., et al. (2011). Effects of genetic variants on lipid parameters and dyslipidemia in a Chinese population. *J. Lipid Res.* 52, 354–360. doi:10.1194/jlr.P007476.

Liu, Y.-L., Patman, G. L., Leathart, J. B. S., Piguet, A.-C., Burt, A. D., Dufour, J.-F., et al. (2014a). Carriage of the PNPLA3 rs738409 C >G polymorphism confers an increased risk of non-alcoholic fatty liver disease associated hepatocellular carcinoma. *J. Hepatol.* 61, 75–81. doi:10.1016/j.jhep.2014.02.030.

Liu, Y.-L., Reeves, H. L., Burt, A. D., Tiniakos, D., McPherson, S., Leathart, J. B. S., et al. (2014b). TM6SF2 rs58542926 influences hepatic fibrosis progression in patients with non-alcoholic fatty liver disease. *Nat. Commun.* 5, 4309. doi:10.1038/ncomms5309.

Loomis, S. J., Li, M., Maruthur, N. M., Baldridge, A. S., North, K. E., Mei, H., et al. (2018). Genome-Wide Association Study of Serum Fructosamine and Glycated Albumin in Adults Without Diagnosed Diabetes: Results From the Atherosclerosis Risk in Communities Study. *Diabetes* 67, 1684–1696. doi:10.2337/db17-1362.

Loria-Kohen, V., Espinosa-Salinas, I., Marcos-Pasero, H., Lourenço-Nogueira, T., Herranz, J., Molina, S., et al. (2016). Polymorphism in the CLOCK gene may influence the effect of fat intake reduction on weight loss. *Nutrition* 32, 453–460. doi:10.1016/j.nut.2015.10.013.

Lu, X., Huang, J., Mo, Z., He, J., Wang, L., Yang, X., et al. (2016a). Genetic Susceptibility to Lipid Levels and Lipid Change Over Time and Risk of Incident Hyperlipidemia in Chinese Populations. *Circ. Cardiovasc. Genet.* 9, 37–44. doi:10.1161/CIRCGENETICS.115.001096.

Lu, X.-L., Yao, X.-Y., Liu, X.-L., Xin, Y., Zhao, L.-L., Wang, Z., et al. (2017). Melatonin receptor 1B gene polymorphism rs10830963 and gestational diabetes mellitus among a Chinese population - a meta-analysis of association studies. *Endokrynol. Pol.* 68, 550–560. doi:10.5603/EP.a2017.0045.

Lu, Y., Day, F. R., Gustafsson, S., Buchkovich, M. L., Na, J., Bataille, V., et al. (2016b). New loci for body fat percentage reveal link between adiposity and cardiometabolic disease risk. *Nat. Commun.* 7, 10495. doi:10.1038/ncomms10495.

Lu, Y., Dollé, M. E. T., Imholz, S., van ’t Slot, R., Verschuren, W. M. M., Wijmenga, C., et al. (2008). Multiple genetic variants along candidate pathways influence plasma high-density lipoprotein cholesterol concentrations. *J. Lipid Res.* 49, 2582–2589. doi:10.1194/jlr.M800232-JLR200.

Lu, Y., Feskens, E. J. M., Boer, J. M. A., Imholz, S., Verschuren, W. M. M., Wijmenga, C., et al. (2010). Exploring genetic

determinants of plasma total cholesterol levels and their predictive value in a longitudinal study. *Atherosclerosis* 213, 200–205. doi:10.1016/j.atherosclerosis.2010.08.053.

Ma, L., Yang, J., Runesha, H. B., Tanaka, T., Ferrucci, L., Bandinelli, S., et al. (2010). Genome-wide association analysis of total cholesterol and high-density lipoprotein cholesterol levels using the Framingham heart study data. *BMC Med. Genet.* 11, 55. doi:10.1186/1471-2350-11-55.

Mahmoudi, T., Farahani, H., Nobakht, H., Dabiri, R., and Zali, M. R. (2016). Genetic Variations in Leptin and Leptin Receptor and Susceptibility to Colorectal Cancer and Obesity. *Iran. J. Cancer Prev.* 9, e7013. doi:10.17795/ijcp-7013.

Mancina, R. M., Spagnuolo, R., Milano, M., Brogneri, S., Morrone, A., Cosco, C., et al. (2016). PNPLA3 148M Carriers with Inflammatory Bowel Diseases Have Higher Susceptibility to Hepatic Steatosis and Higher Liver Enzymes. *Inflamm. Bowel Dis.* 22, 134–140. doi:10.1097/MIB.0000000000000569.

Mangge, H., Baumgartner, B. G., Zelzer, S., Prüller, F., Schnedl, W. J., Reininghaus, E. Z., et al. (2015). Patatin-like phospholipase 3 (rs738409) gene polymorphism is associated with increased liver enzymes in obese adolescents and metabolic syndrome in all ages. *Aliment. Pharmacol. Ther.* 42, 99–105. doi:10.1111/apt.13232.

Manning, A. K., Hivert, M.-F., Scott, R. A., Grimsby, J. L., Bouatia-Naji, N., Chen, H., et al. (2012). A genome-wide approach accounting for body mass index identifies genetic variants influencing fasting glycemic traits and insulin resistance. *Nat. Genet.* 44, 659–669. doi:10.1038/ng.2274.

Manriquez, V., Aviles, J., Salazar, L., Saavedra, N., Seron, P., Lanas, F., et al. (2018). Polymorphisms in Genes Involved in the Leptin-Melanocortin Pathway are Associated with Obesity-Related Cardiometabolic Alterations in a Southern Chilean Population. *Mol. Diagn. Ther.* 22, 101–113. doi:10.1007/s40291-017-0306-8.

Mao, H., Li, Q., and Gao, S. (2012). Meta-analysis of the relationship between common type 2 diabetes risk gene variants with gestational diabetes mellitus. *PloS One* 7, e45882. doi:10.1371/journal.pone.0045882.

Mazzotti, D. R., Singulane, C. C., Ota, V. K., Rodrigues, T. P., Furuya, T. K., de Souza, F. J., et al. (2014). Association of APOE, GCPII and MMP9 polymorphisms with common diseases and lipid levels in an older adult/elderly cohort. *Gene* 535, 370–375. doi:10.1016/j.gene.2013.11.040.

Middelberg, R. P., Ferreira, M. A., Henders, A. K., Heath, A. C., Madden, P. A., Montgomery, G. W., et al. (2011). Genetic variants in LPL, OASL and TOMM40/APOE-C1-C2-C4 genes are associated with multiple cardiovascular-related traits. *BMC Med. Genet.* 12, 123. doi:10.1186/1471-2350-12-123.

Mirzaei, K., Xu, M., Qi, Q., de Jonge, L., Bray, G. A., Sacks, F., et al. (2014). Variants in glucose- and circadian rhythm-related genes affect the response of energy expenditure to weight-loss diets: the POUNDS LOST Trial. *Am. J. Clin. Nutr.* 99, 392–399. doi:10.3945/ajcn.113.072066.

Miyaaki, H., Miuma, S., Taura, N., Shibata, H., Soyama, A., Hidaka, M., et al. (2018). PNPLA3 as a liver steatosis risk factor following living-donor liver transplantation for hepatitis C. *Hepatol. Res. Off. J. Jpn. Soc. Hepatol.* 48, E335–E339. doi:10.1111/hepr.12920.

Mohlke, K. L., Boehnke, M., and Abecasis, G. R. (2008). Metabolic and cardiovascular traits: an abundance of recently identified common genetic variants. *Hum. Mol. Genet.* 17, R102–R108. doi:10.1093/hmg/ddn275.

Mondul, A., Mancina, R. M., Merlo, A., Dongiovanni, P., Rametta, R., Montalcini, T., et al. (2015). PNPLA3 I148M Variant Influences Circulating Retinol in Adults with Nonalcoholic Fatty Liver Disease or Obesity. *J. Nutr.* 145, 1687–1691. doi:10.3945/jn.115.210633.

Moritou, Y., Ikeda, F., Iwasaki, Y., Baba, N., Takaguchi, K., Senoh, T., et al. (2013). Predictive impact of polymorphism of PNPLA3 on HCC development after interferon therapy in Japanese patients with chronic hepatitis C. *SpringerPlus* 2, 251. doi:10.1186/2193-1801-2-251.

Morris, A. P., Voight, B. F., Teslovich, T. M., Ferreira, T., Segrè, A. V., Steinthorsdottir, V., et al. (2012). Large-scale association analysis provides insights into the genetic architecture and pathophysiology of type 2 diabetes. *Nat. Genet.* 44, 981–990. doi:10.1038/ng.2383.

Muendlein, A., Geller-Rhomberg, S., Saely, C. H., Winder, T., Sonderegger, G., Rein, P., et al. (2009). Significant impact of chromosomal locus 1p13.3 on serum LDL cholesterol and on angiographically characterized coronary atherosclerosis. *Atherosclerosis* 206, 494–499. doi:10.1016/j.atherosclerosis.2009.02.040.

Muller, Y. L., Hanson, R. L., Wiessner, G., Nieboer, L., Kobes, S., Piaggi, P., et al. (2015). Assessing FOXO1A as a potential susceptibility locus for type 2 diabetes and obesity in American Indians. *Obes. Silver Spring Md* 23, 1960–1965. doi:10.1002/oby.21236.

Murray, A., Cluett, C., Bandinelli, S., Corsi, A. M., Ferrucci, L., Guralnik, J., et al. (2009). Common lipid-altering gene variants are associated with therapeutic intervention thresholds of lipid levels in older people. *Eur. Heart J.* 30, 1711–1719. doi:10.1093/eurheartj/ehp161.

Müssig, K., Staiger, H., Machicao, F., Stancáková, A., Kuusisto, J., Laakso, M., et al. (2009). Association of common genetic variation in the FOXO1 gene with beta-cell dysfunction, impaired glucose tolerance, and type 2 diabetes. *J. Clin. Endocrinol. Metab.* 94, 1353–1360. doi:10.1210/jc.2008-1048.

Nagy, R., Boutin, T. S., Marten, J., Huffman, J. E., Kerr, S. M., Campbell, A., et al. (2017). Exploration of haplotype research consortium imputation for genome-wide association studies in 20,032 Generation Scotland participants. *Genome Med.* 9, 23. doi:10.1186/s13073-017-0414-4.

Namvaran, F., Azarpira, N., Rahimi-Moghaddam, P., and Dabbaghmanesh, M. H. (2011). Polymorphism of peroxisome proliferator-activated receptor γ (PPARγ) Pro12Ala in the Iranian population: relation with insulin resistance and response to treatment with pioglitazone in type 2 diabetes. *Eur. J. Pharmacol.* 671, 1–6. doi:10.1016/j.ejphar.2011.09.158.

Nelson, C. P., Goel, A., Butterworth, A. S., Kanoni, S., Webb, T. R., Marouli, E., et al. (2017). Association analyses based on false discovery rate implicate new loci for coronary artery disease. *Nat. Genet.* 49, 1385–1391. doi:10.1038/ng.3913.

Nettleton, J. A., Steffen, L. M., Ballantyne, C. M., Boerwinkle, E., and Folsom, A. R. (2007). Associations between HDL-cholesterol and polymorphisms in hepatic lipase and lipoprotein lipase genes are modified by dietary fat intake in African American

and White adults. *Atherosclerosis* 194, e131–e140. doi:10.1016/j.atherosclerosis.2006.11.025.

Nie, M., Wang, Y., Li, W., Ping, F., Liu, J., Wu, X., et al. (2017). The association between six genetic variants and blood lipid levels in pregnant Chinese Han women. *J. Clin. Lipidol.* 11, 938–944. doi:10.1016/j.jacl.2017.06.006.

Nikpay, M., Goel, A., Won, H.-H., Hall, L. M., Willenborg, C., Kanoni, S., et al. (2015). A comprehensive 1,000 Genomes-based genome-wide association meta-analysis of coronary artery disease. *Nat. Genet.* 47, 1121–1130. doi:10.1038/ng.3396.

Niu, C., Luo, Z., Yu, L., Yang, Y., Chen, Y., Luo, X., et al. (2017). Associations of the APOB rs693 and rs17240441 polymorphisms with plasma APOB and lipid levels: a meta-analysis. *Lipids Health Dis.* 16, 166. doi:10.1186/s12944-017-0558-7.

Ogawa, N., Imai, Y., Morita, H., and Nagai, R. (2010). Genome-Wide Association Study of Coronary Artery Disease. *Int. J. Hypertens.* doi:10.4061/2010/790539.

Ohshige, T., Iwata, M., Omori, S., Tanaka, Y., Hirose, H., Kaku, K., et al. (2011). Association of New Loci Identified in European Genome-Wide Association Studies with Susceptibility to Type 2 Diabetes in the Japanese. *PLOS ONE* 6, e26911. doi:10.1371/journal.pone.0026911.

Okada, Y., Takahashi, A., Ohmiya, H., Kumasaka, N., Kamatani, Y., Hosono, N., et al. (2011). Genome-wide association study for C-reactive protein levels identified pleiotropic associations in the IL6 locus. *Hum. Mol. Genet.* 20, 1224–1231. doi:10.1093/hmg/ddq551.

Ollila, H. M., Utge, S., Kronholm, E., Aho, V., Van Leeuwen, W., Silander, K., et al. (2012). TRIB1 constitutes a molecular link between regulation of sleep and lipid metabolism in humans. *Transl. Psychiatry* 2, e97. doi:10.1038/tp.2012.20.

Oniki, K., Saruwatari, J., Izuka, T., Kajiwara, A., Morita, K., Sakata, M., et al. (2015). Influence of the PNPLA3 rs738409 Polymorphism on Non-Alcoholic Fatty Liver Disease and Renal Function among Normal Weight Subjects. *PloS One* 10, e0132640. doi:10.1371/journal.pone.0132640.

Palmer, N. D., Goodarzi, M. O., Langefeld, C. D., Wang, N., Guo, X., Taylor, K. D., et al. (2015). Genetic Variants Associated With Quantitative Glucose Homeostasis Traits Translate to Type 2 Diabetes in Mexican Americans: The GUARDIAN (Genetics Underlying Diabetes in Hispanics) Consortium. *Diabetes* 64, 1853–1866. doi:10.2337/db14-0732.

Pan, Q., Zhang, R.-N., Wang, Y.-Q., Zheng, R.-D., Mi, Y.-Q., Liu, W.-B., et al. (2015). Linked PNPLA3 polymorphisms confer susceptibility to nonalcoholic steatohepatitis and decreased viral load in chronic hepatitis B. *World J. Gastroenterol.* 21, 8605–8614. doi:10.3748/wjg.v21.i28.8605.

Pappa, K. I., Gazouli, M., Anastasiou, E., Iliodromiti, Z., Antsaklis, A., and Anagnou, N. P. (2013). The major circadian pacemaker ARNT-like protein-1 (BMAL1) is associated with susceptibility to gestational diabetes mellitus. *Diabetes Res. Clin. Pract.* 99, 151–157. doi:10.1016/j.diabres.2012.10.015.

Park, M.-H., Kim, N., Lee, J.-Y., and Park, H.-Y. (2011). Genetic loci associated with lipid concentrations and cardiovascular risk factors in the Korean population. *J. Med. Genet.* 48, 10–15. doi:10.1136/jmg.2010.081000.

Petta, S., Grimaudo, S., Cammà, C., Cabibi, D., Di Marco, V., Licata, G., et al. (2012). IL28B and PNPLA3 polymorphisms affect histological liver damage in patients with non-alcoholic fatty liver disease. *J. Hepatol.* 56, 1356–1362.

doi:10.1016/j.jhep.2012.01.007.

Petta, S., Maida, M., Grimaudo, S., Pipitone, R. M., Macaluso, F. S., Cabibi, D., et al. (2016). TM6SF2 rs58542926 is not associated with steatosis and fibrosis in large cohort of patients with genotype 1 chronic hepatitis C. *Liver Int. Off. J. Int. Assoc. Study Liver* 36, 198–204. doi:10.1111/liv.12918.

Phani, N. M., Vohra, M., Rajesh, S., Adhikari, P., Nagri, S. K., D'Souza, S. C., et al. (2016). Implications of critical PPARγ2, ADIPOQ and FTO gene polymorphisms in type 2 diabetes and obesity-mediated susceptibility to type 2 diabetes in an Indian population. *Mol. Genet. Genomics MGG* 291, 193–204. doi:10.1007/s00438-015-1097-4.

Pirazzi, C., Adiels, M., Burza, M. A., Mancina, R. M., Levin, M., Ståhlman, M., et al. (2012). Patatin-like phospholipase domain-containing 3 (PNPLA3) I148M (rs738409) affects hepatic VLDL secretion in humans and in vitro. *J. Hepatol.* 57, 1276–1282. doi:10.1016/j.jhep.2012.07.030.

Pirim, D., Wang, X., Radwan, Z. H., Niemsiri, V., Bunker, C. H., Barmada, M. M., et al. (2015). Resequencing of *LPL* in African Blacks and associations with lipoprotein–lipid levels. *Eur. J. Hum. Genet.* 23, 1244–1253. doi:10.1038/ejhg.2014.268.

Pirola, C. J., and Sookoian, S. (2015). The dual and opposite role of the TM6SF2-rs58542926 variant in protecting against cardiovascular disease and conferring risk for nonalcoholic fatty liver: A meta-analysis. *Hepatol. Baltim. Md* 62, 1742–1756. doi:10.1002/hep.28142.

Pontoriero, A. C., Trinks, J., Hulaniuk, M. L., Caputo, M., Fortuny, L., Pratx, L. B., et al. (2015). Influence of ethnicity on the distribution of genetic polymorphisms associated with risk of chronic liver disease in South American populations. *BMC Genet.* 16, 93. doi:10.1186/s12863-015-0255-3.

Pott, J., Schlegel, V., Teren, A., Horn, K., Kirsten, H., Bluecher, C., et al. (2018). Genetic Regulation of PCSK9 (Proprotein Convertase Subtilisin/Kexin Type 9) Plasma Levels and Its Impact on Atherosclerotic Vascular Disease Phenotypes. *Circ. Genomic Precis. Med.* 11, e001992. doi:10.1161/CIRCGEN.117.001992.

Povel, C. M., Feskens, E. J. M., Imholz, S., Blaak, E. E., Boer, J. M. A., and Dollé, M. E. T. (2010). Glucose levels and genetic variants across transcriptional pathways: interaction effects with BMI. *Int. J. Obes. 2005* 34, 840–845. doi:10.1038/ijo.2009.302.

Poynter, J. N., Jacobs, E. T., Figueiredo, J. C., Lee, W. H., Conti, D. V., Campbell, P. T., et al. (2010). Genetic Variation in the Vitamin D Receptor (VDR) and the Vitamin D–Binding Protein (GC) and Risk for Colorectal Cancer: Results from the Colon Cancer Family Registry. *Cancer Epidemiol. Prev. Biomark.* 19, 525–536. doi:10.1158/1055-9965.EPI-09-0662.

Prokopenko, I., Langenberg, C., Florez, J. C., Saxena, R., Soranzo, N., Thorleifsson, G., et al. (2009). Variants in MTNR1B influence fasting glucose levels. *Nat. Genet.* 41, 77–81. doi:10.1038/ng.290.

Prokopenko, I., Poon, W., Mägi, R., B, R. P., Salehi, S. A., Almgren, P., et al. (2014). A Central Role for GRB10 in Regulation of Islet Function in Man. *PLOS Genet.* 10, e1004235. doi:10.1371/journal.pgen.1004235.

Qi, L., Ma, J., Qi, Q., Hartiala, J., Allayee, H., and Campos, H. (2011a). Genetic risk score and risk of myocardial infarction in Hispanics. *Circulation* 123, 374–380. doi:10.1161/CIRCULATIONAHA.110.976613.

Qi, L., Parast, L., Cai, T., Powers, C., Gervino, E. V., Hauser, T. H., et al. (2011b). Genetic susceptibility to coronary heart

disease in type 2 diabetes: 3 independent studies. *J. Am. Coll. Cardiol.* 58, 2675–2682. doi:10.1016/j.jacc.2011.08.054.

Qi, Q., Stilp, A. M., Sofer, T., Moon, J.-Y., Hidalgo, B., Szpiro, A. A., et al. (2017). Genetics of Type 2 Diabetes in U.S. Hispanic/Latino Individuals: Results From the Hispanic Community Health Study/Study of Latinos (HCHS/SOL). *Diabetes* 66, 1419–1425. doi:10.2337/db16-1150.

Qiu, C., Zeng, P., Li, X., Zhang, Z., Pan, B., Peng, Z. Y. F., et al. (2017). What is the impact of PCSK9 rs505151 and rs11591147 polymorphisms on serum lipids level and cardiovascular risk: a meta-analysis. *Lipids Health Dis.* 16, 111. doi:10.1186/s12944-017-0506-6.

Queiroz, E. M., Cândido, A. P. C., Castro, I. M., Bastos, A. Q. A., Machado-Coelho, G. L. L., and Freitas, R. N. (2015). IGF2, LEPR, POMC, PPARG, and PPARGC1 gene variants are associated with obesity-related risk phenotypes in Brazilian children and adolescents. *Braz. J. Med. Biol. Res.* 48, 595–602. doi:10.1590/1414-431X20154155.

Rafiq, S., Venkata, K. K. M., Gupta, V., Vinay, D., Spurgeon, C. J., Parameshwaran, S., et al. (2012). Evaluation of seven common lipid associated loci in a large Indian sib pair study. *Lipids Health Dis.* 11, 155. doi:10.1186/1476-511X-11-155.

Rasmussen-Torvik, L. J., Alonso, A., Li, M., Kao, W., Köttgen, A., Yan, Y., et al. (2010). Impact of repeated measures and sample selection on genome-wide association studies of fasting glucose. *Genet. Epidemiol.* 34, 665–673. doi:10.1002/gepi.20525.

Rasmussen-Torvik, L. J., Pacheco, J. A., Wilke, R. A., Thompson, W. K., Ritchie, M. D., Kho, A. N., et al. (2012). High Density GWAS for LDL Cholesterol in African Americans Using Electronic Medical Records Reveals a Strong Protective Variant in APOE. *Clin. Transl. Sci.* 5, 394–399. doi:10.1111/j.1752-8062.2012.00446.x.

Rausch, V., Peccerella, T., Lackner, C., Yagmur, E., Seitz, H.-K., Longerich, T., et al. (2016). Primary liver injury and delayed resolution of liver stiffness after alcohol detoxification in heavy drinkers with the PNPLA3 variant I148M. *World J. Hepatol.* 8, 1547–1556. doi:10.4254/wjh.v8.i35.1547.

Regieli, J. J., Jukema, J. W., Doevendans, P. A., Zwinderman, A. H., van der Graaf, Y., Kastelein, J. J., et al. (2009). PPAR gamma variant influences angiographic outcome and 10-year cardiovascular risk in male symptomatic coronary artery disease patients. *Diabetes Care* 32, 839–844. doi:10.2337/dc08-1819.

Reilly, M. P., Li, M., He, J., Ferguson, J. F., Stylianou, I. M., Mehta, N. N., et al. (2011). Identification of ADAMTS7 as a novel locus for coronary atherosclerosis and association of ABO with myocardial infarction in the presence of coronary atherosclerosis: two genome-wide association studies. *The Lancet* 377, 383–392. doi:10.1016/S0140-6736(10)61996-4.

Reinehr, T., Scherag, A., Wang, H.-J., Roth, C. L., Kleber, M., Scherag, S., et al. (2011). Relationship between MTNR1B (melatonin receptor 1B gene) polymorphism rs10830963 and glucose levels in overweight children and adolescents. *Pediatr. Diabetes* 12, 435–441. doi:10.1111/j.1399-5448.2010.00738.x.

Ren, J., Xiang, A. H., Trigo, E., Takayanagi, M., Beale, E., Lawrence, J. M., et al. (2014). Genetic variation in MTNR1B is associated with gestational diabetes mellitus and contributes only to the absolute level of beta cell compensation in Mexican Americans. *Diabetologia* 57, 1391–1399. doi:10.1007/s00125-014-3239-3.

Renström, F., Koivula, R. W., Varga, T. V., Hallmans, G., Mulder, H., Florez, J. C., et al. (2015). Season-dependent

associations of circadian rhythm-regulating loci (CRY1, CRY2 and MTNR1B) and glucose homeostasis: the GLACIER Study. *Diabetologia* 58, 997–1005. doi:10.1007/s00125-015-3533-8.

Renström, F., Shungin, D., Johansson, I., MAGIC Investigators, Florez, J. C., Hallmans, G., et al. (2011). Genetic predisposition to long-term nondiabetic deteriorations in glucose homeostasis: Ten-year follow-up of the GLACIER study. *Diabetes* 60, 345–354. doi:10.2337/db10-0933.

Ridker, P. M., Paré, G., Parker, A. N., Zee, R. Y. L., Miletich, J. P., and Chasman, D. I. (2009). Polymorphism in the CETP gene region, HDL cholesterol, and risk of future myocardial infarction: Genomewide analysis among 18 245 initially healthy women from the Women's Genome Health Study. *Circ. Cardiovasc. Genet.* 2, 26–33. doi:10.1161/CIRCGENETICS.108.817304.

Ríos-González, B. E., Ibarra-Cortés, B., Ramírez-López, G., Sánchez-Corona, J., and Magaña-Torres, M. T. (2014). Association of polymorphisms of genes involved in lipid metabolism with blood pressure and lipid values in mexican hypertensive individuals. *Dis. Markers* 2014, 150358. doi:10.1155/2014/150358.

Ripatti, P., Rämö, J. T., Söderlund, S., Surakka, I., Matikainen, N., Pirinen, M., et al. (2016). The Contribution of GWAS Loci in Familial Dyslipidemias. *PLOS Genet.* 12, e1006078. doi:10.1371/journal.pgen.1006078.

Rodrigues, A. C., Sobrino, B., Genvigir, F. D. V., Willrich, M. a. V., Arazi, S. S., Dorea, E. L., et al. (2013). Genetic variants in genes related to lipid metabolism and atherosclerosis, dyslipidemia and atorvastatin response. *Clin. Chim. Acta Int. J. Clin. Chem.* 417, 8–11. doi:10.1016/j.cca.2012.11.028.

Rojano-Rodriguez, M. E., Beristain-Hernandez, J. L., Zavaleta-Villa, B., Maravilla, P., Romero-Valdovinos, M., and Olivo-Diaz, A. (2016). Leptin receptor gene polymorphisms and morbid obesity in Mexican patients. *Hereditas* 153, 2. doi:10.1186/s41065-016-0006-0.

Romeo, S., Kozlitina, J., Xing, C., Pertsemlidis, A., Cox, D., Pennacchio, L. A., et al. (2008). Genetic variation in PNPLA3 confers susceptibility to nonalcoholic fatty liver disease. *Nat. Genet.* 40, 1461–1465. doi:10.1038/ng.257.

Romeo, S., Sentinelli, F., Cambuli, V. M., Incani, M., Congiu, T., Matta, V., et al. (2010a). The 148M allele of the PNPLA3 gene is associated with indices of liver damage early in life. *J. Hepatol.* 53, 335–338. doi:10.1016/j.jhep.2010.02.034.

Romeo, S., Sentinelli, F., Dash, S., Yeo, G. S. H., Savage, D. B., Leonetti, F., et al. (2010b). Morbid obesity exposes the association between PNPLA3 I148M (rs738409) and indices of hepatic injury in individuals of European descent. *Int. J. Obes. 2005* 34, 190–194. doi:10.1038/ijo.2009.216.

Romero, R., Friel, L. A., Edwards, D. R. V., Kusanovic, J. P., Hassan, S. S., Mazaki-Tovi, S., et al. (2010). A genetic association study of maternal and fetal candidate genes that predispose to preterm prelabor rupture of membranes (PROM). *Am. J. Obstet. Gynecol.* 203, 361.e1-361.e30. doi:10.1016/j.ajog.2010.05.026.

Ronald, J., Rajagopalan, R., Ranchalis, J. E., Marshall, J. K., Hatsukami, T. S., Heagerty, P. J., et al. (2009). Analysis of recently identified dyslipidemia alleles reveals two loci that contribute to risk for carotid artery disease. *Lipids Health Dis.* 8, 52. doi:10.1186/1476-511X-8-52.

Rönn, T., Wen, J., Yang, Z., Lu, B., Du, Y., Groop, L., et al. (2009). A common variant in MTNR1B, encoding melatonin

receptor 1B, is associated with type 2 diabetes and fasting plasma glucose in Han Chinese individuals. *Diabetologia* 52, 830–833. doi:10.1007/s00125-009-1297-8.

Roslin, N. M., Hamid, J. S., Paterson, A. D., and Beyene, J. (2009). Genome-wide association analysis of cardiovascular-related quantitative traits in the Framingham Heart Study. *BMC Proc.* 3, S117.

Rudkowska, I., Dewailly, E., Hegele, R. A., Boiteau, V., Dubé-Linteau, A., Abdous, B., et al. (2013). Gene-diet interactions on plasma lipid levels in the Inuit population. *Br. J. Nutr.* 109, 953–961. doi:10.1017/S0007114512002231.

Rüeger, S., Bochud, P.-Y., Dufour, J.-F., Müllhaupt, B., Semela, D., Heim, M. H., et al. (2015). Impact of common risk factors of fibrosis progression in chronic hepatitis C. *Gut* 64, 1605–1615. doi:10.1136/gutjnl-2014-306997.

Sabatti, C., Service, S. K., Hartikainen, A.-L., Pouta, A., Ripatti, S., Brodsky, J., et al. (2009). Genome-wide association analysis of metabolic traits in a birth cohort from a founder population. *Nat. Genet.* 41, 35–46. doi:10.1038/ng.271.

Safar, H. A., Chehadeh, S. E. H., Abdel-Wareth, L., Haq, A., Jelinek, H. F., ElGhazali, G., et al. (2018). Vitamin D receptor gene polymorphisms among Emirati patients with type 2 diabetes mellitus. *J. Steroid Biochem. Mol. Biol.* 175, 119–124. doi:10.1016/j.jsbmb.2017.03.012.

Sagoo, G. S., Tatt, I., Salanti, G., Butterworth, A. S., Sarwar, N., van Maarle, M., et al. (2008). Seven lipoprotein lipase gene polymorphisms, lipid fractions, and coronary disease: a HuGE association review and meta-analysis. *Am. J. Epidemiol.* 168, 1233–1246. doi:10.1093/aje/kwn235.

Salameh, H., Masadeh, M., Al Hanayneh, M., Petros, V., Maslonka, M., Nanda, A., et al. (2016). PNPLA3 polymorphism increases risk for and severity of chronic hepatitis C liver disease. *World J. Hepatol.* 8, 1584–1592. doi:10.4254/wjh.v8.i35.1584.

Saleheen, D., Soranzo, N., Rasheed, A., Scharnagl, H., Gwilliam, R., Alexander, M., et al. (2010). Genetic determinants of major blood lipids in Pakistanis compared with Europeans. *Circ. Cardiovasc. Genet.* 3, 348–357. doi:10.1161/CIRCGENETICS.109.906180.

Salem, H., Rosenfeld, T., Altarescu, G., Grisaru-Granovsky, S., and Birk, R. (2016). Maternal and neonatal leptin and leptin receptor polymorphisms associated with preterm birth. *Gene* 591, 209–213. doi:10.1016/j.gene.2016.07.014.

Salman, M., Dasgupta, S., Cholendra, A., Venugopal, P. N., Lakshmi, G. L., Xaviour, D., et al. (2015). MTNR1B gene polymorphisms and susceptibility to Type 2 Diabetes: A pilot study in South Indians. *Gene* 566, 189–193. doi:10.1016/j.gene.2015.04.064.

Samani, N. J., Braund, P. S., Erdmann, J., Götz, A., Tomaszewski, M., Linsel-Nitschke, P., et al. (2008). The novel genetic variant predisposing to coronary artery disease in the region of the PSRC1 and CELSR2 genes on chromosome 1 associates with serum cholesterol. *J. Mol. Med. Berl. Ger.* 86, 1233–1241. doi:10.1007/s00109-008-0387-2.

Sandhu, M. S., Waterworth, D. M., Debenham, S. L., Wheeler, E., Papadakis, K., Zhao, J. H., et al. (2008). LDL-cholesterol concentrations: a genome-wide association study. *The Lancet* 371, 483–491. doi:10.1016/S0140-6736(08)60208-1.

Sanghera, D. K., Demirci, F. Y., Been, L., Ortega, L., Ralhan, S., Wander, G. S., et al. (2010). PPARG and ADIPOQ gene polymorphisms increase type 2 diabetes mellitus risk in Asian Indian Sikhs: Pro12Ala still remains as the strongest predictor.

Metabolism. 59, 492–501. doi:10.1016/j.metabol.2009.07.043.

Sanghera, D. K., Ortega, L., Han, S., Singh, J., Ralhan, S. K., Wander, G. S., et al. (2008). Impact of nine common type 2 diabetes risk polymorphisms in Asian Indian Sikhs: PPARG2 (Pro12Ala), IGF2BP2, TCF7L2 and FTOvariants confer a significant risk. *BMC Med. Genet.* 9, 59. doi:10.1186/1471-2350-9-59.

Santoro, N., Kursawe, R., D'Adamo, E., Dykas, D. J., Zhang, C. K., Bale, A. E., et al. (2010). A common variant in the patatin-like phospholipase 3 gene (PNPLA3) is associated with fatty liver disease in obese children and adolescents. *Hepatol. Baltim. Md* 52, 1281–1290. doi:10.1002/hep.23832.

Sato, M., Kato, N., Tateishi, R., Muroyama, R., Kowatari, N., Li, W., et al. (2014). Impact of PNPLA3 polymorphisms on the development of hepatocellular carcinoma in patients with chronic hepatitis C virus infection. *Hepatol. Res. Off. J. Jpn. Soc. Hepatol.* 44, E137-144. doi:10.1111/hepr.12258.

Saxena, R., Voight, B. F., Lyssenko, V., Burtt, N. P., Bakker, P. I. W. de, Chen, H., et al. (2007). Genome-Wide Association Analysis Identifies Loci for Type 2 Diabetes and Triglyceride Levels. *Science* 316, 1331–1336. doi:10.1126/science.1142358.

Scheiner, B., Mandorfer, M., Schwabl, P., Payer, B. A., Bucsics, T., Bota, S., et al. (2015). The Impact of PNPLA3 rs738409 SNP on Liver Fibrosis Progression, Portal Hypertension and Hepatic Steatosis in HIV/HCV Coinfection. *PloS One* 10, e0143429. doi:10.1371/journal.pone.0143429.

Schmidt, A. F., Swerdlow, D. I., Holmes, M. V., Patel, R. S., Fairhurst-Hunter, Z., Lyall, D. M., et al. (2017). PCSK9 genetic variants and risk of type 2 diabetes: a mendelian randomisation study. *Lancet Diabetes Endocrinol.* 5, 97–105. doi:10.1016/S2213-8587(16)30396-5.

Schunkert, H., König, I. R., Kathiresan, S., Reilly, M. P., Assimes, T. L., Holm, H., et al. (2011). Large-scale association analysis identifies 13 new susceptibility loci for coronary artery disease. *Nat. Genet.* 43, 333.

Scott, E. M., Carter, A. M., and Grant, P. J. (2008). Association between polymorphisms in the Clock gene, obesity and the metabolic syndrome in man. *Int. J. Obes. 2005* 32, 658–662. doi:10.1038/sj.ijo.0803778.

Scott, L. J., Mohlke, K. L., Bonnycastle, L. L., Willer, C. J., Li, Y., Duren, W. L., et al. (2007). A genome-wide association study of type 2 diabetes in Finns detects multiple susceptibility variants. *Science* 316, 1341–1345. doi:10.1126/science.1142382.

Seko, Y., Yamaguchi, K., Mizuno, N., Okuda, K., Takemura, M., Taketani, H., et al. (2018). Combination of PNPLA3 and TLL1 polymorphism can predict advanced fibrosis in Japanese patients with nonalcoholic fatty liver disease. *J. Gastroenterol.* 53, 438–448. doi:10.1007/s00535-017-1372-8.

Sevastianova, K., Kotronen, A., Gastaldelli, A., Perttilä, J., Hakkarainen, A., Lundbom, J., et al. (2011). Genetic variation in PNPLA3 (adiponutrin) confers sensitivity to weight loss-induced decrease in liver fat in humans. *Am. J. Clin. Nutr.* 94, 104–111. doi:10.3945/ajcn.111.012369.

Shahid, S. U., Shabana, N. A., Cooper, J. A., Rehman, A., and Humphries, S. E. (2017). Common variants in the genes of triglyceride and HDL-C metabolism lack association with coronary artery disease in the Pakistani subjects. *Lipids Health Dis.* 16, 24. doi:10.1186/s12944-017-0419-4.

Shang, X.-R., Song, J.-Y., Liu, F.-H., Ma, J., and Wang, H.-J. (2015). GWAS-Identified Common Variants With Nonalcoholic Fatty Liver Disease in Chinese Children. *J. Pediatr. Gastroenterol. Nutr.* 60, 669–674. doi:10.1097/MPG.0000000000000662.

Shen, J., Wong, G. L.-H., Chan, H. L.-Y., Chan, H.-Y., Yeung, D. K.-W., Chan, R. S.-M., et al. (2014). PNPLA3 gene polymorphism accounts for fatty liver in community subjects without metabolic syndrome. *Aliment. Pharmacol. Ther.* 39, 532–539. doi:10.1111/apt.12609.

Shen, J.-H., Li, Y.-L., Li, D., Wang, N.-N., Jing, L., and Huang, Y.-H. (2015a). The rs738409 (I148M) variant of the PNPLA3 gene and cirrhosis: a meta-analysis. *J. Lipid Res.* 56, 167–175. doi:10.1194/jlr.M048777.

Shen, O., Ding, X., Nie, J., Xia, Y., Wang, X., Tong, J., et al. (2015b). Variants of the CLOCK gene affect the risk of idiopathic male infertility in the Han-Chinese population. *Chronobiol. Int.* 32, 959–965. doi:10.3109/07420528.2015.1056305.

Shetty Priya B., Tang Hua, Feng Tao, Tayo Bamidele, Morrison Alanna C., Kardia Sharon L.R., et al. (2015). Variants for HDL-C, LDL-C, and Triglycerides Identified from Admixture Mapping and Fine-Mapping Analysis in African American Families. *Circ. Cardiovasc. Genet.* 8, 106–113. doi:10.1161/CIRCGENETICS.114.000481.

Shi, H., Lu, Y., Du, J., Du, W., Ye, X., Yu, X., et al. (2012). Application of back propagation artificial neural network on genetic variants in adiponectin ADIPOQ, peroxisome proliferator-activated receptor-γ, and retinoid X receptor-α genes and type 2 diabetes risk in a Chinese Han population. *Diabetes Technol. Ther.* 14, 293–300. doi:10.1089/dia.2011.0071.

Shimoyama, Y., Mitsuda, Y., Tsuruta, Y., Suzuki, K., Hamajima, N., and Niwa, T. (2012). SIRTUIN 1 gene polymorphisms are associated with cholesterol metabolism and coronary artery calcification in Japanese hemodialysis patients. *J. Ren. Nutr. Off. J. Counc. Ren. Nutr. Natl. Kidney Found.* 22, 114–119. doi:10.1053/j.jrn.2011.10.025.

Shimoyama, Y., Suzuki, K., Hamajima, N., and Niwa, T. (2011). Sirtuin 1 gene polymorphisms are associated with body fat and blood pressure in Japanese. *Transl. Res. J. Lab. Clin. Med.* 157, 339–347. doi:10.1016/j.trsl.2011.02.004.

Shin, S.-Y., Fauman, E. B., Petersen, A.-K., Krumsiek, J., Santos, R., Huang, J., et al. (2014). An atlas of genetic influences on human blood metabolites. *Nat. Genet.* 46, 543–550. doi:10.1038/ng.2982.

Shirali, M., Pong-Wong, R., Navarro, P., Knott, S., Hayward, C., Vitart, V., et al. (2016). Regional heritability mapping method helps explain missing heritability of blood lipid traits in isolated populations. *Heredity* 116, 333–338. doi:10.1038/hdy.2015.107.

Shirts, B. H., Hasstedt, S. J., Hopkins, P. N., and Hunt, S. C. (2011). Evaluation of the gene-age interactions in HDL cholesterol, LDL cholesterol, and triglyceride levels: the impact of the SORT1 polymorphism on LDL cholesterol levels is age dependent. *Atherosclerosis* 217, 139–141. doi:10.1016/j.atherosclerosis.2011.03.008.

Simonis-Bik, A. M., Nijpels, G., Haeften, T. W. van, Houwing-Duistermaat, J. J., Boomsma, D. I., Reiling, E., et al. (2010). Gene Variants in the Novel Type 2 Diabetes Loci CDC123/CAMK1D, THADA, ADAMTS9, BCL11A, and MTNR1B Affect Different Aspects of Pancreatic β-Cell Function. *Diabetes* 59, 293–301. doi:10.2337/db09-1048.

Singal, A. G., Manjunath, H., Yopp, A. C., Beg, M. S., Marrero, J. A., Gopal, P., et al. (2014). The effect of PNPLA3 on fibrosis progression and development of hepatocellular carcinoma: a meta-analysis. *Am. J. Gastroenterol.* 109, 325–334.

doi:10.1038/ajg.2013.476.

Smagris, E., BasuRay, S., Li, J., Huang, Y., Lai, K. V., Gromada, J., et al. (2015). Pnpla3I148M knockin mice accumulate PNPLA3 on lipid droplets and develop hepatic steatosis. *Hepatol. Baltim. Md* 61, 108–118. doi:10.1002/hep.27242.

Smith, E. N., Chen, W., Kähönen, M., Kettunen, J., Lehtimäki, T., Peltonen, L., et al. (2010). Longitudinal genome-wide association of cardiovascular disease risk factors in the Bogalusa heart study. *PLoS Genet.* 6, e1001094. doi:10.1371/journal.pgen.1001094.

Smolková, B., Bonassi, S., Buociková, V., Dušinská, M., Horská, A., Kuba, D., et al. (2015). Genetic determinants of quantitative traits associated with cardiovascular disease risk. *Mutat. Res.* 778, 18–25. doi:10.1016/j.mrfmmm.2015.05.005.

Sookoian, S., Castaño, G., Gemma, C., Gianotti, T.-F., and Pirola, C.-J. (2007). Common genetic variations in CLOCK transcription factor are associated with nonalcoholic fatty liver disease. *World J. Gastroenterol.* 13, 4242–4248.

Sookoian, S., Castaño, G. O., Burgueño, A. L., Gianotti, T. F., Rosselli, M. S., and Pirola, C. J. (2009a). A nonsynonymous gene variant in the adiponutrin gene is associated with nonalcoholic fatty liver disease severity. *J. Lipid Res.* 50, 2111–2116. doi:10.1194/jlr.P900013-JLR200.

Sookoian, S., Castaño, G. O., Scian, R., Mallardi, P., Fernández Gianotti, T., Burgueño, A. L., et al. (2015). Genetic variation in transmembrane 6 superfamily member 2 and the risk of nonalcoholic fatty liver disease and histological disease severity. *Hepatol. Baltim. Md* 61, 515–525. doi:10.1002/hep.27556.

Sookoian, S., Gemma, C., Gianotti, T. F., Burgueño, A., Castaño, G., and Pirola, C. J. (2008). Genetic variants of Clock transcription factor are associated with individual susceptibility to obesity. *Am. J. Clin. Nutr.* 87, 1606–1615.

Sookoian, S., Gianotti, T. F., Burgueño, A., and Pirola, C. J. (2010). Gene-Gene Interaction Between Serotonin Transporter (slc6a4) and Clock Modulates the Risk of Metabolic Syndrome in Rotating Shiftworkers. *Chronobiol. Int.* 27, 1202–1218. doi:10.3109/07420528.2010.496913.

Sookoian, S., Gianotti, T. F., Schuman, M., and Pirola, C. J. (2009b). Gene prioritization based on biological plausibility over genome wide association studies renders new loci associated with type 2 diabetes. *Genet. Med. Off. J. Am. Coll. Med. Genet.* 11, 338–343. doi:10.1097/GIM.0b013e31819995ca.

Sookoian, S., and Pirola, C. J. (2016). Meta-analysis of the influence of *TM6SF2* E167K variant on Plasma Concentration of Aminotransferases across different Populations and Diverse Liver Phenotypes. *Sci. Rep.* 6, 27718. doi:10.1038/srep27718.

Southam, L., Gilly, A., Süveges, D., Farmaki, A.-E., Schwartzentruber, J., Tachmazidou, I., et al. (2017). Whole genome sequencing and imputation in isolated populations identify genetic associations with medically-relevant complex traits. *Nat. Commun.* 8, 15606. doi:10.1038/ncomms15606.

Sparsø, T., Bonnefond, A., Andersson, E., Bouatia-Naji, N., Holmkvist, J., Wegner, L., et al. (2009). G-allele of intronic rs10830963 in MTNR1B confers increased risk of impaired fasting glycemia and type 2 diabetes through an impaired glucose-stimulated insulin release: studies involving 19,605 Europeans. *Diabetes* 58, 1450–1456. doi:10.2337/db08-1660.

Speliotes, E. K., Butler, J. L., Palmer, C. D., Voight, B. F., GIANT Consortium, MIGen Consortium, et al. (2010). PNPLA3

variants specifically confer increased risk for histologic nonalcoholic fatty liver disease but not metabolic disease. *Hepatol. Baltim. Md* 52, 904–912. doi:10.1002/hep.23768.

Speliotes, E. K., Yerges-Armstrong, L. M., Wu, J., Hernaez, R., Kim, L. J., Palmer, C. D., et al. (2011). Genome-Wide Association Analysis Identifies Variants Associated with Nonalcoholic Fatty Liver Disease That Have Distinct Effects on Metabolic Traits. *PLOS Genet.* 7, e1001324. doi:10.1371/journal.pgen.1001324.

Spracklen, C. N., Chen, P., Kim, Y. J., Wang, X., Cai, H., Li, S., et al. (2017). Association analyses of East Asian individuals and trans-ancestry analyses with European individuals reveal new loci associated with cholesterol and triglyceride levels. *Hum. Mol. Genet.* 26, 1770–1784. doi:10.1093/hmg/ddx062.

Staiger, H., Machicao, F., Schäfer, S. A., Kirchhoff, K., Kantartzis, K., Guthoff, M., et al. (2008). Polymorphisms within the novel type 2 diabetes risk locus MTNR1B determine beta-cell function. *PloS One* 3, e3962. doi:10.1371/journal.pone.0003962.

Stancáková, A., Kuulasmaa, T., Paananen, J., Jackson, A. U., Bonnycastle, L. L., Collins, F. S., et al. (2009). Association of 18 confirmed susceptibility loci for type 2 diabetes with indices of insulin release, proinsulin conversion, and insulin sensitivity in 5,327 nondiabetic Finnish men. *Diabetes* 58, 2129–2136. doi:10.2337/db09-0117.

Steffen, K., Cooper, M., Shi, M., Caprau, D., Simhan, H., Dagle, J., et al. (2007). Maternal and fetal variation in genes of cholesterol metabolism is associated with preterm delivery. *J. Perinatol. Off. J. Calif. Perinat. Assoc.* 27, 672–680. doi:10.1038/sj.jp.7211806.

Stickel, F., Buch, S., Lau, K., Meyer zu Schwabedissen, H., Berg, T., Ridinger, M., et al. (2011). Genetic variation in the PNPLA3 gene is associated with alcoholic liver injury in caucasians. *Hepatol. Baltim. Md* 53, 86–95. doi:10.1002/hep.24017.

Stojkovic, I. A., Ericson, U., Rukh, G., Riddestråle, M., Romeo, S., and Orho-Melander, M. (2014). The PNPLA3 Ile148Met interacts with overweight and dietary intakes on fasting triglyceride levels. *Genes Nutr.* 9, 388. doi:10.1007/s12263-014-0388-4.

Stuebe, A. M., Wise, A., Nguyen, T., Herring, A., North, K. E., and Siega-Riz, A. M. (2014). Maternal genotype and gestational diabetes. *Am. J. Perinatol.* 31, 69–76. doi:10.1055/s-0033-1334451.

Suhre, K., Arnold, M., Bhagwat, A. M., Cotton, R. J., Engelke, R., Raffler, J., et al. (2017). Connecting genetic risk to disease end points through the human blood plasma proteome. *Nat. Commun.* 8, 14357. doi:10.1038/ncomms14357.

Sun, B. B., Maranville, J. C., Peters, J. E., Stacey, D., Staley, J. R., Blackshaw, J., et al. (2018). Genomic atlas of the human plasma proteome. *Nature* 558, 73–79. doi:10.1038/s41586-018-0175-2.

Surakka, I., Horikoshi, M., Mägi, R., Sarin, A.-P., Mahajan, A., Lagou, V., et al. (2015). The impact of low-frequency and rare variants on lipid levels. *Nat. Genet.* 47, 589–597. doi:10.1038/ng.3300.

Tabassum, R., Mahendran, Y., Dwivedi, O. P., Chauhan, G., Ghosh, S., Marwaha, R. K., et al. (2012). Common variants of IL6, LEPR, and PBEF1 are associated with obesity in Indian children. *Diabetes* 61, 626–631. doi:10.2337/db11-1501.

Taherzadeh-Fard, E., Saft, C., Akkad, D. A., Wieczorek, S., Haghikia, A., Chan, A., et al. (2011). PGC-1alpha downstream transcription factors NRF-1 and TFAM are genetic modifiers of Huntington disease. *Mol. Neurodegener.* 6, 32. doi:10.1186/1750-1326-6-32.

Tai, C.-M., Huang, C.-K., Tu, H.-P., Hwang, J.-C., Chang, C.-Y., and Yu, M.-L. (2015). PNPLA3 genotype increases susceptibility of nonalcoholic steatohepatitis among obese patients with nonalcoholic fatty liver disease. *Surg. Obes. Relat. Dis. Off. J. Am. Soc. Bariatr. Surg.* 11, 888–894. doi:10.1016/j.soard.2014.07.016.

Tai, C.-M., Huang, C.-K., Tu, H.-P., Hwang, J.-C., Yeh, M.-L., Huang, C.-F., et al. (2016). Interactions of a PPARGC1A Variant and a PNPLA3 Variant Affect Nonalcoholic Steatohepatitis in Severely Obese Taiwanese Patients. *Medicine (Baltimore)* 95, e3120. doi:10.1097/MD.0000000000003120.

Takeuchi, F., Isono, M., Katsuya, T., Yokota, M., Yamamoto, K., Nabika, T., et al. (2012). Association of Genetic Variants Influencing Lipid Levels with Coronary Artery Disease in Japanese Individuals. *PLOS ONE* 7, e46385. doi:10.1371/journal.pone.0046385.

Takeuchi, Y., Ikeda, F., Moritou, Y., Hagihara, H., Yasunaka, T., Kuwaki, K., et al. (2013). The impact of patatin-like phospholipase domain-containing protein 3 polymorphism on hepatocellular carcinoma prognosis. *J. Gastroenterol.* 48, 405–412. doi:10.1007/s00535-012-0647-3.

Tam, C. H. T., Ho, J. S. K., Wang, Y., Lee, H. M., Lam, V. K. L., Germer, S., et al. (2010). Common polymorphisms in MTNR1B, G6PC2 and GCK are associated with increased fasting plasma glucose and impaired beta-cell function in Chinese subjects. *PloS One* 5, e11428. doi:10.1371/journal.pone.0011428.

Tan, A., Sun, J., Xia, N., Qin, X., Hu, Y., Zhang, S., et al. (2012). A genome-wide association and gene-environment interaction study for serum triglycerides levels in a healthy Chinese male population. *Hum. Mol. Genet.* 21, 1658–1664. doi:10.1093/hmg/ddr587.

Tan, L.-J., Zhu, H., He, H., Wu, K.-H., Li, J., Chen, X.-D., et al. (2014). Replication of 6 Obesity Genes in a Meta-Analysis of Genome-Wide Association Studies from Diverse Ancestries. *PLOS ONE* 9, e96149. doi:10.1371/journal.pone.0096149.

Tang, C. S., Zhang, H., Cheung, C. Y. Y., Xu, M., Ho, J. C. Y., Zhou, W., et al. (2015). Exome-wide association analysis reveals novel coding sequence variants associated with lipid traits in Chinese. *Nat. Commun.* 6, 10206. doi:10.1038/ncomms10206.

Tang, W., Apostol, G., Schreiner, P. J., Jacobs, D. R., Boerwinkle, E., and Fornage, M. (2010). Associations of lipoprotein lipase gene polymorphisms with longitudinal plasma lipid trends in young adults: The Coronary Artery Risk Development in Young Adults (CARDIA) study. *Circ. Cardiovasc. Genet.* 3, 179–186. doi:10.1161/CIRCGENETICS.109.913426.

Tarnowski, M., Malinowski, D., Safranow, K., Dziedziejko, V., and Pawlik, A. (2017). MTNR1A and MTNR1B gene polymorphisms in women with gestational diabetes. *Gynecol. Endocrinol. Off. J. Int. Soc. Gynecol. Endocrinol.* 33, 395–398. doi:10.1080/09513590.2016.1276556.

Tejedor, M. T., Garcia-Sobreviela, M. P., Ledesma, M., and Arbones-Mainar, J. M. (2014). The Apolipoprotein E Polymorphism rs7412 Associates with Body Fatness Independently of Plasma Lipids in Middle Aged Men. *PLoS ONE* 9. doi:10.1371/journal.pone.0108605.

Tellechea, M. L., Aranguren, F., Pérez, M. S., Cerrone, G. E., Frechtel, G. D., and Taverna, M. J. (2009). Pro12Ala Polymorphism of the Peroxisome ProliferatorActivated Receptor-γ Gene is Associated With Metabolic Syndrome and Surrogate

Measures of Insulin Resistance in Healthy Men. *Circ. J.* 73, 2118–2124. doi:10.1253/circj.CJ-09-0320.

Teslovich, T. M., Musunuru, K., Smith, A. V., Edmondson, A. C., Stylianou, I. M., Koseki, M., et al. (2010). Biological, Clinical, and Population Relevance of 95 Loci for Blood Lipids. *Nature* 466, 707–713. doi:10.1038/nature09270.

Trépo, E., Gustot, T., Degré, D., Lemmers, A., Verset, L., Demetter, P., et al. (2011a). Common polymorphism in the PNPLA3/adiponutrin gene confers higher risk of cirrhosis and liver damage in alcoholic liver disease. *J. Hepatol.* 55, 906–912. doi:10.1016/j.jhep.2011.01.028.

Trepo, E., Guyot, E., Ganne-Carrie, N., Degre, D., Gustot, T., Franchimont, D., et al. (2012). PNPLA3 (rs738409 C>G) is a common risk variant associated with hepatocellular carcinoma in alcoholic cirrhosis. *Hepatol. Baltim. Md* 55, 1307–1308. doi:10.1002/hep.25518.

Trépo, E., Nahon, P., Bontempi, G., Valenti, L., Falleti, E., Nischalke, H.-D., et al. (2014). Association between the PNPLA3 (rs738409 C>G) variant and hepatocellular carcinoma: Evidence from a meta-analysis of individual participant data. *Hepatol. Baltim. Md* 59, 2170–2177. doi:10.1002/hep.26767.

Trépo, E., Pradat, P., Potthoff, A., Momozawa, Y., Quertinmont, E., Gustot, T., et al. (2011b). Impact of patatin-like phospholipase-3 (rs738409 C>G) polymorphism on fibrosis progression and steatosis in chronic hepatitis C. *Hepatol. Baltim. Md* 54, 60–69. doi:10.1002/hep.24350.

Trombetta, M., Bonetti, S., Boselli, M. L., Miccoli, R., Trabetti, E., Malerba, G., et al. (2013). PPARG2 Pro12Ala and ADAMTS9 rs4607103 as "insulin resistance loci" and "insulin secretion loci" in Italian individuals. The GENFIEV study and the Verona Newly Diagnosed Type 2 Diabetes Study (VNDS) 4. *Acta Diabetol.* 50, 401–408. doi:10.1007/s00592-012-0443-9.

Tsai, C.-W., North, K. E., Tin, A., Haack, K., Franceschini, N., Saroja Voruganti, V., et al. (2015). Both rare and common variants in PCSK9 influence plasma low-density lipoprotein cholesterol level in American Indians. *J. Clin. Endocrinol. Metab.* 100, E345-349. doi:10.1210/jc.2014-3340.

Tsuzaki, K., Kotani, K., Sano, Y., Fujiwara, S., Takahashi, K., and Sakane, N. (2010). The association of the Clock 3111 T/C SNP with lipids and lipoproteins including small dense low-density lipoprotein: results from the Mima study. *BMC Med. Genet.* 11, 150. doi:10.1186/1471-2350-11-150.

Uemura H., Katsuura-Kamano S., Yamaguchi M., Arisawa K., Hamajima N., Hishida A., et al. (2016). Variant of the clock circadian regulator (CLOCK) gene and related haplotypes are associated with the prevalence of type 2 diabetes in the Japanese population. *J. Diabetes* 8, 667–676. doi:10.1111/1753-0407.12344.

Ueyama, M., Nishida, N., Korenaga, M., Korenaga, K., Kumagai, E., Yanai, H., et al. (2016). The impact of PNPLA3 and JAZF1 on hepatocellular carcinoma in non-viral hepatitis patients with type 2 diabetes mellitus. *J. Gastroenterol.* 51, 370–379. doi:10.1007/s00535-015-1116-6.

Urbanek, M., Hayes, M. G., Lee, H., Freathy, R. M., Lowe, L. P., Ackerman, C., et al. (2012). The Role of Inflammatory Pathway Genetic Variation on Maternal Metabolic Phenotypes during Pregnancy. *PLOS ONE* 7, e32958. doi:10.1371/journal.pone.0032958.

Uygun, A., Ozturk, K., Demirci, H., Oztuna, A., Eren, F., Kozan, S., et al. (2017). The association of nonalcoholic fatty liver disease with genetic polymorphisms: a multicenter study. *Eur. J. Gastroenterol. Hepatol.* 29, 441–447. doi:10.1097/MEG.0000000000000813.

Valenti, L., Alisi, A., Galmozzi, E., Bartuli, A., Del Menico, B., Alterio, A., et al. (2010a). I148M patatin-like phospholipase domain-containing 3 gene variant and severity of pediatric nonalcoholic fatty liver disease. *Hepatol. Baltim. Md* 52, 1274–1280. doi:10.1002/hep.23823.

Valenti, L., Al-Serri, A., Daly, A. K., Galmozzi, E., Rametta, R., Dongiovanni, P., et al. (2010b). Homozygosity for the patatin-like phospholipase-3/adiponutrin I148M polymorphism influences liver fibrosis in patients with nonalcoholic fatty liver disease. *Hepatol. Baltim. Md* 51, 1209–1217. doi:10.1002/hep.23622.

Valenti, L., Rametta, R., Ruscica, M., Dongiovanni, P., Steffani, L., Motta, B. M., et al. (2012). The I148M PNPLA3 polymorphism influences serum adiponectin in patients with fatty liver and healthy controls. *BMC Gastroenterol.* 12, 111. doi:10.1186/1471-230X-12-111.

Valladares, M., Obregón, A. M., and Chaput, J.-P. (2015). Association between genetic variants of the clock gene and obesity and sleep duration. *J. Physiol. Biochem.* 71, 855–860. doi:10.1007/s13105-015-0447-3.

van der Harst, P., and Verweij, N. (2018). Identification of 64 Novel Genetic Loci Provides an Expanded View on the Genetic Architecture of Coronary Artery Disease. *Circ. Res.* 122, 433–443. doi:10.1161/CIRCRESAHA.117.312086.

Varga, T. V., Sonestedt, E., Shungin, D., Koivula, R. W., Hallmans, G., Escher, S. A., et al. (2014). Genetic determinants of long-term changes in blood lipid concentrations: 10-year follow-up of the GLACIER study. *PLoS Genet.* 10, e1004388. doi:10.1371/journal.pgen.1004388.

Verrijken, A., Beckers, S., Francque, S., Hilden, H., Caron, S., Zegers, D., et al. (2013). A gene variant of PNPLA3, but not of APOC3, is associated with histological parameters of NAFLD in an obese population. *Obes. Silver Spring Md* 21, 2138–2145. doi:10.1002/oby.20366.

Vespasiani-Gentilucci, U., Gallo, P., Porcari, A., Carotti, S., Galati, G., Piccioni, L., et al. (2016). The PNPLA3 rs738409 C > G polymorphism is associated with the risk of progression to cirrhosis in NAFLD patients. *Scand. J. Gastroenterol.* 51, 967–973. doi:10.3109/00365521.2016.1161066.

Viganò, M., Valenti, L., Lampertico, P., Facchetti, F., Motta, B. M., D'Ambrosio, R., et al. (2013). Patatin-like phospholipase domain-containing 3 I148M affects liver steatosis in patients with chronic hepatitis B. *Hepatol. Baltim. Md* 58, 1245–1252. doi:10.1002/hep.26445.

Viitasalo, A., Pihlajamaki, J., Lindi, V., Atalay, M., Kaminska, D., Joro, R., et al. (2015). Associations of I148M variant in PNPLA3 gene with plasma ALT levels during 2-year follow-up in normal weight and overweight children: the PANIC Study. *Pediatr. Obes.* 10, 84–90. doi:10.1111/ijpo.234.

Villard, E. F., EI Khoury, P., Frisdal, E., Bruckert, E., Clement, K., Bonnefont-Rousselot, D., et al. (2013). Genetic determination of plasma cholesterol efflux capacity is gender-specific and independent of HDL-cholesterol levels. *Arterioscler.*

Thromb. Vasc. Biol. 33, 822–828. doi:10.1161/ATVBAHA.112.300979.

Villegas, R., Williams, S. M., Gao, Y.-T., Long, J., Shi, J., Cai, H., et al. (2014). Genetic variation in the peroxisome proliferator-activated receptor (PPAR) and peroxisome proliferator-activated receptor gamma co-activator 1 (PGC1) gene families and type 2 diabetes. *Ann. Hum. Genet.* 78, 23–32. doi:10.1111/ahg.12044.

Wagenknecht, L. E., Palmer, N. D., Bowden, D. W., Rotter, J. I., Norris, J. M., Ziegler, J., et al. (2011). Association of PNPLA3 with non-alcoholic fatty liver disease in a minority cohort: the Insulin Resistance Atherosclerosis Family Study. *Liver Int. Off. J. Int. Assoc. Study Liver* 31, 412–416. doi:10.1111/j.1478-3231.2010.02444.x.

Wakil, S. M., Ram, R., Muiya, N. P., Andres, E., Mazhar, N., Hagos, S., et al. (2016). A common variant association study reveals novel susceptibility loci for low HDL-cholesterol levels in ethnic Arabs. *Clin. Genet.* 90, 518–525. doi:10.1111/cge.12761.

Walia, G. K., Gupta, V., Aggarwal, A., Asghar, M., Dudbridge, F., Timpson, N., et al. (2014). Association of common genetic variants with lipid traits in the Indian population. *PloS One* 9, e101688. doi:10.1371/journal.pone.0101688.

Walker, C. G., Loos, R. J. F., Olson, A. D., Frost, G. S., Griffin, B. A., Lovegrove, J. A., et al. (2011). Genetic predisposition influences plasma lipids of participants on habitual diet, but not the response to reductions in dietary intake of saturated fatty acids. *Atherosclerosis* 215, 421–427. doi:10.1016/j.atherosclerosis.2010.12.039.

Wallace, C., Newhouse, S. J., Braund, P., Zhang, F., Tobin, M., Falchi, M., et al. (2008). Genome-wide association study identifies genes for biomarkers of cardiovascular disease: serum urate and dyslipidemia. *Am. J. Hum. Genet.* 82, 139–149. doi:10.1016/j.ajhg.2007.11.001.

Wang, A. Z., Li, L., Zhang, B., Shen, G.-Q., and Wang, Q. K. (2011a). Association of SNP rs17465637 on chromosome 1q41 and rs599839 on 1p13.3 with myocardial infarction in an American caucasian population. *Ann. Hum. Genet.* 75, 475–482. doi:10.1111/j.1469-1809.2011.00646.x.

Wang, J., Ban, M. R., Zou, G. Y., Cao, H., Lin, T., Kennedy, B. A., et al. (2008). Polygenic determinants of severe hypertriglyceridemia. *Hum. Mol. Genet.* 17, 2894–2899. doi:10.1093/hmg/ddn188.

Wang, L., Jing, J., Fu, Q., Tang, X., Su, L., Wu, S., et al. (2015a). Association study of genetic variants at newly identified lipid gene TRIB1 with coronary heart disease in Chinese Han population. *Lipids Health Dis.* 14, 46. doi:10.1186/s12944-015-0043-0.

Wang, P., Wang, Q., Yin, Y., Yang, Z., Li, W., Liang, D., et al. (2015b). Association between Peroxisome Proliferator-activated Receptor Gamma Gene Polymorphisms and Atherosclerotic Diseases: A Meta-analysis of Case-control Studies. *J. Atheroscler. Thromb.* 22, 912–925. doi:10.5551/jat.26138.

Wang, X., Liu, Z., Wang, K., Wang, Z., Sun, X., Zhong, L., et al. (2016). Additive Effects of the Risk Alleles of PNPLA3 and TM6SF2 on Non-alcoholic Fatty Liver Disease (NAFLD) in a Chinese Population. *Front. Genet.* 7. doi:10.3389/fgene.2016.00140.

Wang, Y., and Liu, C. (2012). Quantitative evaluation of common polymorphism (rs1801282) in the PPARγ2 gene and hypertension susceptibility. *Gene* 502, 159–162. doi:10.1016/j.gene.2012.04.035.

Wang, Y., Nie, M., Li, W., Ping, F., Hu, Y., Ma, L., et al. (2011b). Association of six single nucleotide polymorphisms with gestational diabetes mellitus in a Chinese population. *PloS One* 6, e26953. doi:10.1371/journal.pone.0026953.

Waterworth, D. M., Ricketts, S. L., Song, K., Chen, L., Zhao, J. H., Ripatti, S., et al. (2010). Genetic variants influencing circulating lipid levels and risk of coronary artery disease. *Arterioscler. Thromb. Vasc. Biol.* 30, 2264–2276. doi:10.1161/ATVBAHA.109.201020.

Webb, T. R., Erdmann, J., Stirrups, K. E., Stitziel, N. O., Masca, N. G. D., Jansen, H., et al. (2017). Systematic Evaluation of Pleiotropy Identifies 6 Further Loci Associated With Coronary Artery Disease. *J. Am. Coll. Cardiol.* 69, 823–836. doi:10.1016/j.jacc.2016.11.056.

Webster, R. J., Warrington, N. M., Weedon, M. N., Hattersley, A. T., McCaskie, P. A., Beilby, J. P., et al. (2009). The association of common genetic variants in the APOA5, LPL and GCK genes with longitudinal changes in metabolic and cardiovascular traits. *Diabetologia* 52, 106–114. doi:10.1007/s00125-008-1175-9.

Wheeler, E., Leong, A., Liu, C.-T., Hivert, M.-F., Strawbridge, R. J., Podmore, C., et al. (2017). Impact of common genetic determinants of Hemoglobin A1c on type 2 diabetes risk and diagnosis in ancestrally diverse populations: A transethnic genome-wide meta-analysis. *PLoS Med.* 14, e1002383. doi:10.1371/journal.pmed.1002383.

White, M. J., Eren, F., Ağırbaşlı, D., Chen, J., Hu, T., Moore, J. H., et al. (2015). A systems genetics approach to dyslipidemia in children and adolescents. *Omics J. Integr. Biol.* 19, 248–259. doi:10.1089/omi.2014.0140.

Willer, C. J., Sanna, S., Jackson, A. U., Scuteri, A., Bonnycastle, L. L., Clarke, R., et al. (2008). Newly identified loci that influence lipid concentrations and risk of coronary artery disease. *Nat. Genet.* 40, 161–169. doi:10.1038/ng.76.

Willer, C. J., Schmidt, E. M., Sengupta, S., Peloso, G. M., Gustafsson, S., Kanoni, S., et al. (2013). Discovery and refinement of loci associated with lipid levels. *Nat. Genet.* 45, 1274–1283. doi:10.1038/ng.2797.

Winkler, T. W., Justice, A. E., Graff, M., Barata, L., Feitosa, M. F., Chu, S., et al. (2015). The Influence of Age and Sex on Genetic Associations with Adult Body Size and Shape: A Large-Scale Genome-Wide Interaction Study. *PLOS Genet.* 11, e1005378. doi:10.1371/journal.pgen.1005378.

Wood, A. R., Jonsson, A., Jackson, A. U., Wang, N., van Leewen, N., Palmer, N. D., et al. (2017). A Genome-Wide Association Study of IVGTT-Based Measures of First-Phase Insulin Secretion Refines the Underlying Physiology of Type 2 Diabetes Variants. *Diabetes* 66, 2296–2309. doi:10.2337/db16-1452.

Woon, P. Y., Kaisaki, P. J., Bragança, J., Bihoreau, M.-T., Levy, J. C., Farrall, M., et al. (2007a). Aryl hydrocarbon receptor nuclear translocator-like (BMAL1) is associated with susceptibility to hypertension and type 2 diabetes. *Proc. Natl. Acad. Sci. U. S. A.* 104, 14412–14417. doi:10.1073/pnas.0703247104.

Workalemahu, T., Enquobahrie, D. A., Moore, A., Sanchez, S. E., Ananth, C. V., Pacora, P. N., et al. (2013). Genome-wide and candidate gene association studies of placental abruption. *Int. J. Mol. Epidemiol. Genet.* 4, 128–139.

Wu, B., and Pankow, J. S. (2018). Fast and Accurate Genome-Wide Association Test of Multiple Quantitative Traits. *Comput. Math. Methods Med.* 2018. doi:10.1155/2018/2564531.

Wu, L., Cui, L., Tam, W. H., Ma, R. C. W., and Wang, C. C. (2016). Genetic variants associated with gestational diabetes mellitus: a meta-analysis and subgroup analysis. *Sci. Rep.* 6, 30539. doi:10.1038/srep30539.

Wu, L., and Sun, D. (2017). Leptin Receptor Gene Polymorphism and the Risk of Cardiovascular Disease: A Systemic Review and Meta-Analysis. *Int. J. Environ. Res. Public. Health* 14. doi:10.3390/ijerph14040375.

Wu, Y., Marvelle, A. F., Li, J., Croteau-Chonka, D. C., Feranil, A. B., Kuzawa, C. W., et al. (2013). Genetic association with lipids in CLHNS: waist circumference modifies an APOA5 effect on triglyceride levels. *J. Lipid Res.*, jlr.P042077. doi:10.1194/jlr.P042077.

Xia, M.-F., Ling, Y., Bian, H., Lin, H.-D., Yan, H.-M., Chang, X.-X., et al. (2016). I148M variant of PNPLA3 increases the susceptibility to non-alcoholic fatty liver disease caused by obesity and metabolic disorders. *Aliment. Pharmacol. Ther.* 43, 631–642. doi:10.1111/apt.13521.

Xiao, R., Sun, S., Zhang, J., Ouyang, Y., Zhang, N., Yang, M., et al. (2017). Association analysis of APO gene polymorphisms with ischemic stroke risk: a case-control study in a Chinese Han population. *Oncotarget* 8, 60496–60503. doi:10.18632/oncotarget.15549.

Xu, R., Tao, A., Zhang, S., Deng, Y., and Chen, G. (2015). Association between patatin-like phospholipase domain containing 3 gene (PNPLA3) polymorphisms and nonalcoholic fatty liver disease: a HuGE review and meta-analysis. *Sci. Rep.* 5, 9284. doi:10.1038/srep09284.

Xue, A., Wu, Y., Zhu, Z., Zhang, F., Kemper, K. E., Zheng, Z., et al. (2018). Genome-wide association analyses identify 143 risk variants and putative regulatory mechanisms for type 2 diabetes. *Nat. Commun.* 9, 2941. doi:10.1038/s41467-018-04951-w.

Yang, M. M., Wang, J., Fan, J. J., Ng, T. K., Sun, D. J., Guo, X., et al. (2016). Variations in the Obesity Gene "LEPR" Contribute to Risk of Type 2 Diabetes Mellitus: Evidence from a Meta-Analysis. *J. Diabetes Res.* 2016, 5412084. doi:10.1155/2016/5412084.

Yang, R., Li, L., Seidelmann, S. B., Shen, G.-Q., Sharma, S., Rao, S., et al. (2010). A genome-wide linkage scan identifies multiple quantitative trait loci for HDL-cholesterol levels in families with premature CAD and MI. *J. Lipid Res.* 51, 1442–1451. doi:10.1194/jlr.M004325.

Yao, M., Guo, H., He, J., Yan, Y., Ma, R., Ding, Y., et al. (2016a). Interactions of Six SNPs in ABCA1gene and Obesity in Low HDL-C Disease in Kazakh of China. *Int. J. Environ. Res. Public. Health* 13, 176. doi:10.3390/ijerph13020176.

Yao, M.-H., He, J., Ma, R.-L., Ding, Y.-S., Guo, H., Yan, Y.-Z., et al. (2016b). Association between Polymorphisms and Haplotype in the ABCA1 Gene and Overweight/Obesity Patients in the Uyghur Population of China. *Int. J. Environ. Res. Public. Health* 13, 220. doi:10.3390/ijerph13020220.

Yasui, K., Kawaguchi, T., Shima, T., Mitsuyoshi, H., Seki, K., Sendo, R., et al. (2015). Effect of PNPLA3 rs738409 variant (I148 M) on hepatic steatosis, necroinflammation, and fibrosis in Japanese patients with chronic hepatitis C. *J. Gastroenterol.* 50, 887–893. doi:10.1007/s00535-014-1018-z.

Ye, D., Cai, S., Jiang, X., Ding, Y., Chen, K., Fan, C., et al. (2016). Associations of polymorphisms in circadian genes with abdominal obesity in Chinese adult population. *Obes. Res. Clin. Pract.* 10, S133–S141. doi:10.1016/j.orcp.2016.02.002.

Ye, H., Zhao, Q., Huang, Y., Wang, L., Liu, H., Wang, C., et al. (2014). Meta-Analysis of Low Density Lipoprotein Receptor

(LDLR) rs2228671 Polymorphism and Coronary Heart Disease. *BioMed Res. Int.* doi:10.1155/2014/564940.

Yeo, A., Li, L., Warren, L., Aponte, J., Fraser, D., King, K., et al. (2017). Pharmacogenetic meta-analysis of baseline risk factors, pharmacodynamic, efficacy and tolerability endpoints from two large global cardiovascular outcomes trials for darapladib. *PLOS ONE* 12, e0182115. doi:10.1371/journal.pone.0182115.

Yuan, P., Wang, S., Zhou, F., Wan, S., Yang, Y., Huang, X., et al. (2014). Functional polymorphisms in the NPAS2 gene are associated with overall survival in transcatheter arterial chemoembolization-treated hepatocellular carcinoma patients. *Cancer Sci.* 105, 825–832. doi:10.1111/cas.12428.

Yue, Y.-H., Liu, L.-Y., Hu, L., Li, Y.-M., Mao, J.-P., Yang, X.-Y., et al. (2017). The association of lipid metabolism relative gene polymorphisms and ischemic stroke in Han and Uighur population of Xinjiang. *Lipids Health Dis.* 16, 120. doi:10.1186/s12944-017-0491-9.

Zain, S. M., Mohamed, R., Mahadeva, S., Cheah, P. L., Rampal, S., Basu, R. C., et al. (2012). A multi-ethnic study of a PNPLA3 gene variant and its association with disease severity in non-alcoholic fatty liver disease. *Hum. Genet.* 131, 1145–1152. doi:10.1007/s00439-012-1141-y.

Zayani, N., Omezzine, A., Boumaiza, I., Achour, O., Rebhi, L., Rejeb, J., et al. (2017). Association of ADIPOQ, leptin, LEPR, and resistin polymorphisms with obesity parameters in Hammam Sousse Sahloul Heart Study. *J. Clin. Lab. Anal.* 31. doi:10.1002/jcla.22148.

Zeggini, E., Weedon, M. N., Lindgren, C. M., Frayling, T. M., Elliott, K. S., Lango, H., et al. (2007). Replication of genome-wide association signals in UK samples reveals risk loci for type 2 diabetes. *Science* 316, 1336–1341. doi:10.1126/science.1142364.

Zhang, L., You, W., Zhang, H., Peng, R., Zhu, Q., Yao, A., et al. (2015). PNPLA3 polymorphisms (rs738409) and non-alcoholic fatty liver disease risk and related phenotypes: a meta-analysis. *J. Gastroenterol. Hepatol.* 30, 821–829. doi:10.1111/jgh.12889.

Zhang, Y., Cai, W., Song, J., Miao, L., Zhang, B., Xu, Q., et al. (2014a). Association between the PNPLA3 I148M polymorphism and non-alcoholic fatty liver disease in the Uygur and Han ethnic groups of northwestern China. *PloS One* 9, e108381. doi:10.1371/journal.pone.0108381.

Zhang, Y., Sun, C.-M., Hu, X.-Q., and Zhao, Y. (2014b). Relationship between melatonin receptor 1B and insulin receptor substrate 1 polymorphisms with gestational diabetes mellitus: a systematic review and meta-analysis. *Sci. Rep.* 4, 6113. doi:10.1038/srep06113.

Zhang, Z., Ma, F., Zhou, F., Chen, Y., Wang, X., Zhang, H., et al. (2014c). Functional polymorphisms of circadian negative feedback regulation genes are associated with clinical outcome in hepatocellular carcinoma patients receiving radical resection. *Med. Oncol. Northwood Lond. Engl.* 31, 179. doi:10.1007/s12032-014-0179-1.

Zhao, B., Lu, J., Yin, J., Liu, H., Guo, X., Yang, Y., et al. (2012). A functional polymorphism in PER3 gene is associated with prognosis in hepatocellular carcinoma. *Liver Int. Off. J. Int. Assoc. Study Liver* 32, 1451–1459. doi:10.1111/j.1478-3231.2012.02849.x.

Zhao, Q., Xiao, J., He, J., Zhang, X., Hong, J., Kong, X., et al. (2014). Cross-Sectional and Longitudinal Replication Analyses of Genome-Wide Association Loci of Type 2 Diabetes in Han Chinese. *PLOS ONE* 9, e91790. doi:10.1371/journal.pone.0091790.

Zhao, W., Rasheed, A., Tikkanen, E., Lee, J.-J., Butterworth, A. S., Howson, J. M. M., et al. (2017). Identification of new susceptibility loci for type 2 diabetes and shared etiological pathways with coronary heart disease. *Nat. Genet.* 49, 1450–1457. doi:10.1038/ng.3943.

Zheng, C., Dalla Man, C., Cobelli, C., Groop, L., Zhao, H., Bale, A. E., et al. (2015). A common variant in the MTNR1b gene is associated with increased risk of impaired fasting glucose (IFG) in youth with obesity. *Obes. Silver Spring Md* 23, 1022–1029. doi:10.1002/oby.21030.

Zhong, X., Du, Y., Lei, Y., Liu, N., Guo, Y., and Pan, T. (2015). Effects of vitamin D receptor gene polymorphism and clinical characteristics on risk of diabetic retinopathy in Han Chinese type 2 diabetes patients. *Gene* 566, 212–216. doi:10.1016/j.gene.2015.04.045.

Zhou, L., Ding, H., Zhang, X., He, M., Huang, S., Xu, Y., et al. (2011). Genetic variants at newly identified lipid loci are associated with coronary heart disease in a Chinese Han population. *PloS One* 6, e27481. doi:10.1371/journal.pone.0027481.

Zhou, L., He, M., Mo, Z., Wu, C., Yang, H., Yu, D., et al. (2013). A Genome Wide Association Study Identifies Common Variants Associated with Lipid Levels in the Chinese Population. *PLOS ONE* 8, e82420. doi:10.1371/journal.pone.0082420.

Zhou, Y.-J., Hong, S.-C., Yang, Q., Yin, R.-X., Cao, X.-L., and Chen, W.-X. (2015). Association of variants in CELSR2-PSRC1-SORT1 with risk of serum lipid traits, coronary artery disease and ischemic stroke. *Int. J. Clin. Exp. Pathol.* 8, 9543–9551.

Zhu, L., Huang, Q., Xie, Z., Kang, M., Ding, H., Chen, B., et al. (2017a). PPARGC1A rs3736265 G>A polymorphism is associated with decreased risk of type 2 diabetes mellitus and fasting plasma glucose level. *Oncotarget* 8, 37308–37320. doi:10.18632/oncotarget.16307.

Zhu, Y., Zhang, D., Zhou, D., Li, Z., Li, Z., Fang, L., et al. (2017b). Susceptibility loci for metabolic syndrome and metabolic components identified in Han Chinese: a multi-stage genome-wide association study. *J. Cell. Mol. Med.* 21, 1106–1116. doi:10.1111/jcmm.13042.
